# Comparative Effectiveness of ICA and PCA in Extraction of Fetal ECG From Abdominal Signals: Toward Non-invasive Fetal Monitoring

**DOI:** 10.3389/fphys.2018.00648

**Published:** 2018-05-30

**Authors:** Radek Martinek, Radana Kahankova, Janusz Jezewski, Rene Jaros, Jitka Mohylova, Marcel Fajkus, Jan Nedoma, Petr Janku, Homer Nazeran

**Affiliations:** ^1^Department of Cybernetics and Biomedical Engineering, Faculty of Electrical Engineering and Computer Science, VSB-Technical University of Ostrava, Ostrava, Czechia; ^2^Institute of Medical Technology and Equipment ITAM, Zabrze, Poland; ^3^Department of General Electrical Engineering, Faculty of Electrical Engineering and Computer Science, VSB-Technical University of Ostrava, Ostrava, Czechia; ^4^Department of Telecommunications, Faculty of Electrical Engineering and Computer Science, VSB-Technical University of Ostrava, Ostrava, Czechia; ^5^Department of Obstetrics and Gynecology, Masaryk University and University Hospital Brno, Brno, Czechia; ^6^Department of Electrical and Computer Engineering, University of Texas El Paso, El Paso, TX, United States

**Keywords:** electronic fetal monitoring (EFM), fetal electrocardiogram (fECG), non-invasive fetal heart rate (NI-fHR) estimation, non-invasive fetal ECG (NI-fECG), nonadaptive methods, independent component analysis (ICA), principal component analysis (PCA), non-invasive ST analysis (NI-STAN)

## Abstract

Non-adaptive signal processing methods have been successfully applied to extract fetal electrocardiograms (fECGs) from maternal abdominal electrocardiograms (aECGs); and initial tests to evaluate the efficacy of these methods have been carried out by using synthetic data. Nevertheless, performance evaluation of such methods using real data is a much more challenging task and has neither been fully undertaken nor reported in the literature. Therefore, in this investigation, we aimed to compare the effectiveness of two popular non-adaptive methods (the ICA and PCA) to explore the non-invasive (NI) extraction (separation) of fECGs, also known as NI-fECGs from aECGs. The performance of these well-known methods was enhanced by an adaptive algorithm, compensating amplitude difference and time shift between the estimated components. We used real signals compiled in 12 recordings (real01–real12). Five of the recordings were from the publicly available database (PhysioNet-Abdominal and Direct Fetal Electrocardiogram Database), which included data recorded by multiple abdominal electrodes. Seven more recordings were acquired by measurements performed at the Institute of Medical Technology and Equipment, Zabrze, Poland. Therefore, in total we used 60 min of data (i.e., around 88,000 R waves) for our experiments. This dataset covers different gestational ages, fetal positions, fetal positions, maternal body mass indices (BMI), etc. Such a unique heterogeneous dataset of sufficient length combining continuous Fetal Scalp Electrode (FSE) acquired and abdominal ECG recordings allows for robust testing of the applied ICA and PCA methods. The performance of these signal separation methods was then comprehensively evaluated by comparing the fetal Heart Rate (fHR) values determined from the extracted fECGs with those calculated from the fECG signals recorded directly by means of a reference FSE. Additionally, we tested the possibility of non-invasive ST analysis (NI-STAN) by determining the T/QRS ratio. Our results demonstrated that even though these advanced signal processing methods are suitable for the non-invasive estimation and monitoring of the fHR information from maternal aECG signals, their utility for further morphological analysis of the extracted fECG signals remains questionable and warrants further work.

## 1. Introduction

Electronic fetal monitoring (EFM) is a routine monitoring modality during labor and delivery in developed countries. Currently, Doppler ultrasound and fetal electrocardiography (both invasive and non-invasive) are recognized as reliable and proven techniques for monitoring the Fetal Heart Rate (fHR) (Jezewski et al., 2017). The monitoring of fHR using Doppler ultrasound, also called Cardiotocography (CTG), is well established as it is considered effective and is therefore widely used in clinics. However, the fetal Electrocardiogram-based (fECG-based) EFM seems to offer a more promising approach Jezewski et al. (2017) and Hasan et al. (2009b), as it significantly outperforms the Doppler-based CTG, especially during the early stages of labor (Reinhard et al., 2012). Moreover, Reinhard et al. (2013) have concluded that the intrapartum fHR monitoring using the CTG has the disadvantage of more maternal and fetal HR ambiguity compared to the ECG, which additionally provides the maternal Heart Rate (mHR) information.

The non-invasive variants of fECG signals are sensed by abdominal electrodes, amplified and filtered by proper analog signal processing circuitry with adequate gain and bandwidth. Nevertheless, besides the desired fECG signal, there are many other unwanted components originating from biological sources (maternal and fetal muscles, stomach, uterus) as well as technical noise from the powerlines and their surrounding electrical devices that contaminate the recorded abdominal ECG (aECG) signals. Most of these unwanted signals can be eliminated by conventional signal processing techniques (linear filtering) because their frequency ranges are different from the spectrum of the desired (fECG) signals (Sameni and Clifford, 2010). The main challenge in fECG signal processing is suppressing the maternal component, i.e., the maternal electrocardiogram (mECG). Since both signals overlap in the time and frequency domains, advanced signal processing methods must be used to extract the fECG signals.

There are several methods that can be used for extracting fECG component from aECG signals. Generally, they can be divided into two main groups: adaptive and non-adaptive. In our previous research reported elsewhere (Martinek and Zidek, 2012; Martinek et al., 2016a, 2017a,b; Fajkus et al., 2017) we used the adaptive methods and achieved good results in suppressing the mECG signals present in the aECG signals. In spite of producing good outcomes, these methods have the disadvantage of requiring additional thoracic electrodes to provide reference mECG signals. This fact affects the patient's comfort, and complicates its usage in clinical practice. Furthermore, the efficiency of the adaptive methods is closely connected with the adaptive filter's settings. The optimal settings vary with gestational age, fetal position in the uterus, etc. (Martinek et al., 2017a).

From a practical clinical point of view, non-adaptive methods offer the advantage of using ECG signals acquired from the abdominal electrodes alone. In addition, there is a trend toward using SMART technologies in medicine. Thus a multisource system, where the electrodes are embedded within a flexible garment (one electrode grid/strip for the abdominal area) seems to be very promising for the future of continuous fetal monitoring. In such a system, the ICA and PCA methods presented in this article could prove to be the most suitable for implementation. Consequently, to complement our previous work and compare the effectiveness of non-adaptive signal processing methods in suppressing the mECG signals, we carried out the current investigation focusing on the Independent Component Analysis (ICA) and Principal Component Analysis (PCA). The results of our initial experiments applying the popular PCA and ICA methods to synthetic data are presented and reported elsewhere (Kahankova et al., 2017a). The next logical step was to expand our previous work and perform our experiments using real data. The main difference between using synthetic versus real data is in the evaluation of the results. In case of synthetic data, we can benefit from the fact that the ideal fECG signal is available to be used as the reference for objective quality assessment based on metrics such as: Signal-to-Noise Ratio (SNRin, SNRout), Percentage Root Difference (PRD), Root Mean Square Error (RMSE), etc. However, for the experiments with real data, the efficacy of the applied methods can only be evaluated based on the reference signal recorded by the FSE, which is considered as a “gold standard” for fHR determination.

In this investigation, we evaluated the comparative efficacy of the ICA and PCA methods by using real data from a publicly available database (Jezewski et al., 2012) as well as those acquired at the Institute of Medical Technology and Equipment, Zabrze, Poland. Our results show that these non-adaptive methods are suitable for the extraction of fetal heart rate (fHR) information from fECG signals. However, their utility for further morphological analysis of these signals is questionable.

Nevertheless, the results of morphological analysis showed that it is possible to extract relevant information such as the T/QRS ratio from the estimated fECG signals contingent upon ensuring the acquisition of high quality data (elimination of the motion artifacts, correct electrode placement, suffient electrode-skin contact, etc.). In other words, reliable morphological analysis of high quality fECG signals offers the possibility of introducing the non-invasive STAN (NI-STAN) to clinical practice, which would in turn lead to a significant reduction in unnecessary C-sections due to misrepresented EFM results.

## 2. State of the art

The fetal heart rate undergoes dynamic adjustments as it responds to the fetal environment and other stimuli. The changes in fHR can reflect both physiological and pathological influences. Physiological changes are associated with fetal movements and also with maternal contractions during labor (the so-called accelerations and decelerations Williams and Arulkumaran, 2004). A decrease or an increase in fHR which is not a response to physiological events may be a sign of pathology such as fetal hypoxia, i.e., the inadequate supply of oxygen to the fetus (Chandraharan and Arulkumaran, 2007). Therefore, it is essential to monitor fHR, fetal movements and uterine contractions simultaneously. In other words, Electronic Fetal Monitoring (EFM) is a method for observing and controlling a variety of underlying physiological measures at the same time and therefore it enables the detection of any unusual changes in fHR.

### 2.1. Electronic fetal monitoring instrumentation

Electronic fetal monitoring (EFM) using Cardiotocography (CTG) is the most frequently used tool to assess fetal well-being during labor and delivery. The fHR and uterine contractions are detected by two external transducers placed on the maternal abdomen. One of the transducers, placed above the fetal heart, uses Doppler ultrasound to detect fetal heart motion. The second transducer, placed at the fundus of the uterus, measures the frequency of the uterine contractions (Sweha et al., 1999).

The disadvantage of CTG is that it tends to produce false-positive (FP) results. These in turn lead to increased rates of unnecessary caesarian sections, thereby increasing labor and delivery costs Vintzileos et al. (1995). One of the reasons for the production of FP results is the problematic interpretation of CTG, which suffers from large inter-observer disagreement (see Bernardes et al., 1997; Blix et al., 2003; Vayssière et al., 2010; Blackwell et al., 2011; Hruban et al., 2015). This is clearly noticeable when we compare the clinical expert interpretation of the fetal Heart Rate Variability (fHRV) with those generated by computerized systems (Jezewski et al., 2002). The accuracy of the CTG method can be increased by using an internal probe that measures the fetal heart rate directly from the fetal scalp (Amer-Wåhlin et al., 2001; Jezewski et al., 2012). This is, however, inconvenient for the mother and the fetus. Some authors have suggested that fetal heart rate signal interpretation can be improved by applying advanced signal processing techniques (Wróbel et al., 2013; Wrobel et al., 2015; Jezewski et al., 2016).

In addition to CTG, fHR can be obtained from fECG signals. Recent studies show that this method is the most promising one (Jezewski et al., 2017). The monitoring of fECG is performed by internal or external means. The internal monitoring of fHR is performed by attaching a screw-type Fetal Scalp Electrode (FSE) on the fetus forehead. At the same time, uterine contractions are recorded by using an Intrauterine Pressure Catheter (IUPC) placed in the uterus through the cervix. This approach ensures accuracy; however, it poses a risk of infection for the mother and the fetus (Peters et al., 2001; Neilson, 2006). Moreover, its utilization is limited by several factors. The fetal membranes have to be ruptured and the cervix must be at least partially dilated before the FSE can be placed on the fetal scalp.

External fetal monitoring is performed by using surface electrodes that are placed on the maternal abdomen. The internal monitoring of fHR is performed by attaching a screw-type Fetal Scalp Electrode (FSE) on the examiner (Burattini et al., 2015). However, the placement of the electrodes significantly influences the quality of the signal as well as the demands on the system that processes it Martinek et al. (2017b). Compared to internal monitoring, this approach is less stressful and dangerous for the mother and her fetus (Neilson, 2006). Moreover, it can be theoretically used from quite early stages of pregnancy (Kahankova et al., 2017c). Nevertheless, the drawback of this method is that the desired signal (fECG) is contaminated by a large amount of noise. The main source of the noise is of course the maternal body. These biological signals include breathing and muscular activity, motion artifacts and maternal ECG (mECG). Most of these unwanted signals may be reduced by classical filtering methods because frequency ranges of these contaminating signals are different from those of the fECG signal. However, as the mECG signals overlap with fECG signals in the time and frequency domains, they cannot be filtered out by using conventional methods.

### 2.2. Non-invasive fetal electrocardiogarm (NI-fECG) signal extraction methods

Fetal ECG signals can be extracted from either single-channel or multi-channel sources. These signals can be processed by means of adaptive or non-adaptive methods. Even though many different techniques have been implemented to extract the fECG signals (Viunytskyi and Shulgin, 2017), researchers still strive for more accurate and improved non-invasive fetal ECG (NI-fECG) extraction (separation) methods.

Adaptive methods use filters that are able to automatically self-adjust their coefficients (control parameters) according to the information in the filtered signal. These methods are often utilized for noise suppression in a variety of applications, where the source of the signal is known and measurable, such as Channel Equalization (Martinek and Zidek, 2014; Martinek et al., 2015c), Speech Noise Removal (Martinek et al., 2015a), and others. In NI-fECG signal extraction, the maternal component is considered to be the noise in the composite aECG signal, which is comprised of fECG as well as the mECG in addition to other unwanted biological and technical contaminating components. Therefore, adaptive methods can be used for the mECG signal suppression, where the aECG is the primary output and the mECG, recorded by means of the maternal thoracic leads, is the reference input.

#### 2.2.1. Adaptive methods

Adaptive methods can be divided into two groups: Linear and Nonlinear. Linear adaptive methods that have been applied to the NI-fECG signal extraction problem include algorithms based on Kalman Filtering (KF), (Niknazar et al., 2013), Adaptive Voltera Filtering (Shadaydeh et al., 2008), Comb Filtering (Wei et al., 2013), Stochastic Gradient Adaptation, i.e., the Least Mean Squares (LMS) method (Poularikas and Zayed, 2006; Swarnalatha and Prasad, 2010; Kahankova et al., 2018), and algorithms based on optimal recursive adaptation, i.e., the Recursive Least Squares (RLS) method (Poularikas and Zayed, 2006; Swarnalatha and Prasad, 2010; Martinek et al., 2016a; Kahankova et al., 2017d). Adaptive Linear Neuron or Adaptive Linear Element (ADALINE) (Reaz and Wei, 2004; Jia et al., 2010; Amin et al., 2011), and so on.

Nonlinear methods are based on Artificial Intelligence (AI) and those that have been applied to extract the NI-fECG signals include: Adaptive Neural Networks (ANN), Hasan et al. (2009a), Hybrid Neural Networks (HNN), Assaleh (2007), Genetic Algorithms and Bayesian Adaptive Filtering Frameworks Talha et al. (2010), Kam and Cohen (1999), as well as techniques utilizing Adaptive Neuro-Fuzzy Interference System (ANFIS), Assaleh (2007), Al-Zaben and Al-Smadi (2006), Martinek et al. (2016c), and Martinek and Zidek (2012). In addition, Artificial Neural Networks based on Logical Interpretation of fuzzy if-then Rules (ANBLIR) has been introduced as an approach to classify fetal cardiotocograms Czabanski et al. (2008). The main difference between the Linear and Nonlinear approaches is that the latter methods capture the underlying nonlinear characteristics of the body and thus are theoretically more suitable for NI-fECG extraction.

#### 2.2.2. Non-adaptive methods

Non-adaptive filtering methods eliminate the undesired signals to yield the fECG signal without filter adaptation. More specifically, in some of these methods, filter weights are determined by using some initial training data and remain constant. These methods can use either a single-channel or multi-channel signal source. Techniques utilizing a multi-channel signal source include multiple and single-source methods. Single-channel signal source methods are based on for example Wavelet Transform (WT), Karvounis et al. (2004), Datian and Xuemei (1996), Hassanpour and Parsaei (2007), Bsoul (2015), Ivanushkina et al. (2014), Abburi and Chandrasekhara Sastry (2012), Bensafia et al. (2017), Castillo et al. (2013), Correlation Techniques De Araujo et al. (2005), Averaging Techniques (AT) Hon and Lee (1963), Hon and Lee (1964), Template Subtraction Tsui et al. (2017), Singular Value Decomposition (SVD) Kanjilal et al. (1997), Adaptive Noise Canceler (ANC) Zhang et al. (2017), and so on.

The multi-source methods are based on Subspace Denoising Fatemi and Sameni (2017) or Blind Source Separation (BSS), namely: Independent Component Analysis (ICA), Martín-Clemente et al. (2011), Vrins et al. (2004), Najafabadi et al. (2006), Mochimaru et al. (2004), Sameni et al. (2007), De Lathauwer et al. (2000), Marossero et al. (2003), Gurve et al. (2017), Billeci and Maurizio (2017); Principal Component Analysis (PCA), Gurve et al. (2017), Kahankova et al. (2017b), and so on. BSS is a frequently used method for fECG signal filtering. It assumes the statistical independence of the two processed signals: fECG and mECG. It can be applied in the case of multi-channel abdominal recording with the assumption that the signals from different leads are a linear combination of independent signal sources generated by the maternal and fetal hearts (Sameni et al., 2007). The challenge, however, is that the relationship between the mECG recorded on the maternal chest and the mECG in the abdominal signal is rather nonlinear in nature. It is important to emphasize that the greater the number of channels, the better the quality of the extracted fECG signal. However, a large number of electrodes is clinically difficult to use and, moreover, they are unpleasant for the patient (Burattini et al., 2015).

De Lathauwer et al. applied the ICA method to fECG signal processing and explained that it is a rather demanding approach (De Lathauwer et al., 2000). They focused on the direct reconstruction of various statistically independent bioelectric signal sources while considering both the maternal and fetal hearts as important sources of diagnostic information, and paid special attention to the propagation characteristics of these signals toward the recording electrodes. Their solution is nonparametric and it is not based on sample averaging, which may be a problem while detecting and analyzing atypical changes in heart rate.

Marrosero et al. compared three ICA-based methods: the Mermaid algorithm, Infomax, and Fast Independent Component Analysis (FastICA), Marossero et al. (2003). By detecting fHR from both real and synthetic records, they showed that the Mermaid method outperforms the other two algorithms. It also appears that this method is more efficient in batch and on-line operating modes, which is vital for real-time implementation.

Sameni et al. applied the ICA method to extract fECG signals, while taking into account the dimensionality of these signals and the theory of the heart dipole (Sameni et al., 2007). In their work, the interpretation of the independent components obtained from multiple leads was closely related to the representation of vectorcardiograms of individual signals. The subspaces of the fetal and maternal ECG signals are not completely different. By using dynamic filters, it should be possible to use a dynamic model that is time-synchronous with mECG and thereby to remove the maternal component while preserving the fetal one.

## 3. Methods

**Figure 1:**
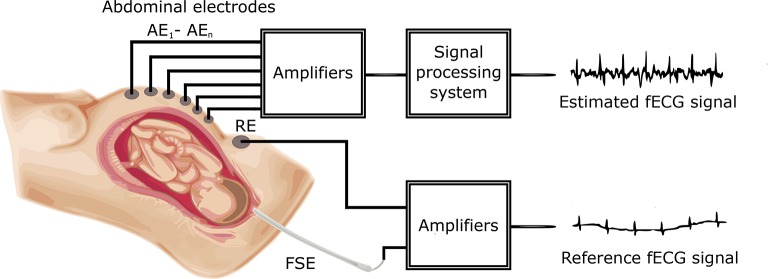
Block diagram of the experimental setup for fECG signal extraction and quality assessment.

Figure 1 shows a block diagram of the experimental setup used for our investigation. The main aim of this investigation was to process aECG signals in order to extract fECG signals from them by applying and comparing two popular non-adapative signal processing (ICA and PCA) methods and then successfully determine the fetal Heart Rate fHR, which is one of the main indicators used to detect and diagnose fetal hypoxia (Hyvarinen and Oja, 2000).

The aECG signals were first pre-processed by FIR bandpass filtering to remove baseline wander, and subsequently applied to the ICA and PCA signal processing blocks to extract the fECG signals. The filtered fECG signals were then compared with the direct (reference) fECG signals recorded by means of the FSE using the fHR values determined from the extracted as well as direct fECG signals. The FSE recordings were quality assured by clinical experts resulting in a set of reference markers precisely indicating the time of occurrence of the R-waves. Thus these transvaginal records could be considered as a “gold standard” for fHR determination and a relevant reference for validating the outcomes of our experiments. Additionally, the results were statistically evaluated by means of the Bland-Altman method. We also provide the graphical interpretation of the results to facilitate the visual evaluation of the fECG signal separation process.

In addition to fHR determination, this article also focuses on testing possible morphological analysis (MA) of the fECG signals, namely non-invasive ST analysis (NI-STAN). We analyzed the estimated as well as the reference fECG signals and also carried out the evaluation of the MA as well.

Non-adaptive methods have been applied to the fECG signal extraction problem (Kahankova et al., 2017b), and initial investigations have been carried out by using synthetic data from a novel signal generator (Martinek et al., 2015b, 2016b). Nevertheless, performance verification of the applied methods using real data is a more complex task than testing them with synthetic signals.

In this research, we used abdominal ECG signals from a publicly available database as well as data acquired in Poland (for details please see section 5). Each recording in the Polish data included four aECG signals. One recording could be tested by:(1) using 11 possible combinations of electrodes; (2) 6 possible combinations when utilizing 2 signal channels; (3) 4 possible combinations when using 3 channels; and (4) 1 combination when using all of the channels simultaneously.

### 3.1. Independent component analysis (ICA)

**Figure 2:**
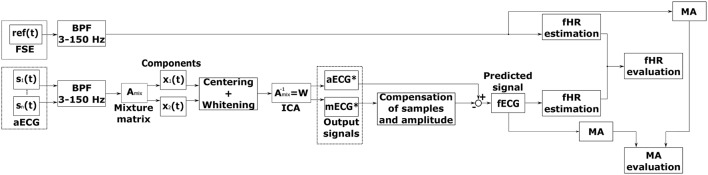
Block diagram of fECG signal extraction using the ICA method.

Figure 2 shows the block diagram for the optimized ICA method. The pre-processing block includes a bandpass FIR filter (with a bandwidth from 3 to 150 Hz) for isoline drift (baseline wander) correction. The pre-processed signals were subsequently applied to the ICA signal processing block, which produced the estimated aECG^*^ signals (with increased amplitude and enhanced fetal and mECG^*^ components, respectively). There was a time shift between the estimated aECG^*^ and mECG^*^ components which needed to be centered. Moreover, it was necessary to equalize the amplitudes of both components in order to ensure that the maternal components had the same amplitude. For this task, we developed an adaptive algorithm that was able to correct the amplitude and phase shifts (see block called Compensation of samples and amplitude in Figure 2). This led to a significant increase in the fECG extraction performance. Finally, the fECG signals were extracted by subtracting the mECG^*^ from aECG^*^ signals. Beside fHR determination, this article also focuses on testing possible morphological analysis (MA) of fECG, namely non-invasive ST analysis. We analyzed the estimated as well as the reference fECG signal and evaluated results (MA evaluation).

**Figure 3:**
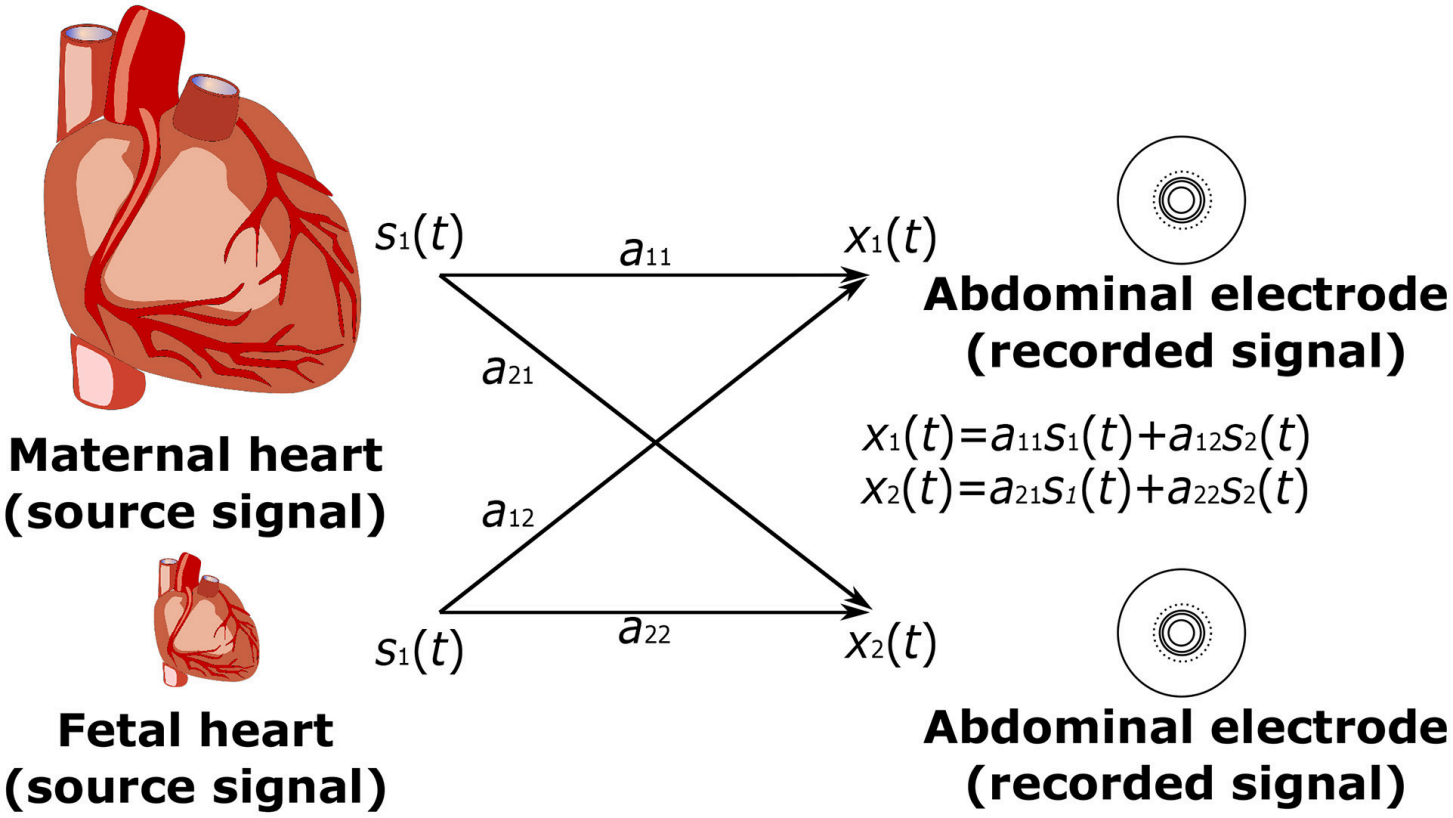
Principle of the ICA method: linear mixture of the unobserved independent source signals - *s*_1_(*t*), *s*_2_(*t*): source signals; and *x*_1_(*t*), *x*_2_(*t*): recorded signals.

Figure 3 shows a depiction of the BSS extraction method based on the ‘Cocktail Party Problem’ adapted for pregnant women as signal sources. There are two signal sources in the maternal body, maternal and fetal heart, producing signals that can be measured by means of electrodes placed on the maternal abdomen. Abdominal electrodes measure both maternal and fetal signals. The signal from the first electrode is marked as *x*_1_(*t*) and the second as *x*_2_(*t*). Each of these recorded signals was generated by weighing the signals *s*_1_(*t*) and *s*_2_(*t*) of the individual signal sources (hearts). The relationship between the signals obtained from the electrodes *x*_*i*_(*t*) and the signals generated by the individual hearts *s*_*i*_(*t*) can be expressed as follows:

(1) $$\begin{array}{r} x_{1}\left(t\right)=a_{11}\left(t\right)s_{1}\left(t\right)+a_{12}\left(t\right)s_{2}\left(t\right), \end{array}$$

(2) $$\begin{array}{r} x_{2}\left(t\right)=a_{21}\left(t\right)s_{1}\left(t\right)+a_{22}\left(t\right)s_{2}\left(t\right). \end{array}$$

#### 3.1.1. ICA data model

Independent Component Analysis (ICA) is a statistical analysis technique used to decompose a multivariable signal into a set of mutually independent, non-Gaussian components, assuming that the measured signals are a combination of independent source signals described mathematically by the ICA model (Karhunen, 1996; Hyvärinen and Oja, 1997; Hyvarinen and Oja, 2000; Černošek et al., 2000; Mohylova et al., 2001):

(3) $$\begin{array}{r} \text{x}=\text{A}\text{s}, \end{array}$$

where again $\text{x}={\left[x_{1},x_{2},\cdots \;,x_{n},\right]}^{\text{T}}$ is the observed multivariate signal, $\text{s}={\left[s_{1},s_{2},\cdots \;,s_{m},\right]}^{\text{T}}$ is the original unknown multivariate source signal, *m* is the number of observed signals, *n* is the number of sources and **A** is the mixing matrix. The values of the signals are considered samples (instantiations) of the random variables, not functions of time. The aim of ICA is to return the linear unmixing matrix **W** in order to acquire the estimated independent components **y** such that:

(4) $$\begin{array}{r} \text{y}=\text{W}\text{x}. \end{array}$$

We assumed here that the number of independent components **s** is equal to the number of observed variables; this is a simplifying assumption that is not completely necessary. If the unknown mixing matrix **A** is square and non-singular, then **W** is the inverse matrix **W** = **A**^−1^. Otherwise, the best unmixing matrix, that separates sources as independent as possible, is given by the generalized inverse Penrose-Moore matrix:

(5) $$\begin{array}{r} \text{W}={\text{A}}^{+}\text{and}||\text{y}-\text{s}||=\text{min}. \end{array}$$

#### 3.1.2. Preprocessing for ICA

The primary reasons for pre-processing are:

- simplification of algorithms,
- reduction of dimensionality of the problem,
- reduction of number of parameters to be estimated,
- highlighting features of the data set not readily explained by the mean and covariance.

There are two main pre-processing strategies in ICA, namely centering and whitening/sphering.

**a) Centering** Centered vectors have zero mean. Centering is a very simple operation and simply refers to subtracting the mean *E*{**x**}:
  (6) $$\begin{array}{r} {\text{x}}_{c}=\text{x}-E\left\{\text{x}\right\}. \end{array}$$
**b) Whitening** Whitened vectors have unit variance. Whitening can be performed by using the eigenvalue decomposition of the covariance matrix:
  (7) $$\begin{array}{r} E\left\{{\text{x}}_{c}{\text{x}}_{c}^{\text{T}}\right\}=\text{V}\text{D}{\text{V}}^{\text{T}}, \end{array}$$
  where **V** is the orthogonal matrix of eigenvectors and **D** is the diagonal matrix of its eigenvalues. A new whitened vector is created as follows:
  (8) $$\begin{array}{r} {\text{x}}_{w}=\text{V}{\text{D}}^{-1/2}{\text{V}}^{\text{T}}{\text{x}}_{c}. \end{array}$$

#### 3.1.3. Limitations of ICA

There are four basic limitations of the ICA method Lee et al. (1998):

1. Only one original independent component can have Gaussian distribution. If multiple Gaussian sources exist, the ICA method is not able to extract these sources (independent components) from the data (*x*).
2. If we have an *n*-dimensional data vector, then we can find a maximum of *n*-independent components using the ICA method.
3. Some (or all) calculated *y* components can be multiplied by -1 with respect to the original components.
4. The order of the original independent components cannot be determined by the ICA method.

### 3.2. Principal component analysis (PCA)

The PCA (Soong and Koles, 1995; Diamantaras and Kung, 1996; Jolliffe, 2002; Lhotská et al., 2009) is a useful statistical technique for finding patterns in data of high dimensionality. It is a way of identifying patterns in data, and expressing the data in such a way as to highlight their similarities and differences. Since patterns can be hard to find in data of high dimension, where the luxury of graphical representation is not available, PCA is a powerful tool for analyzing data.

**Figure 4:**
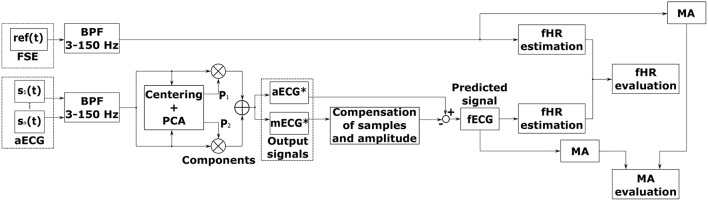
Block diagram for fECG signal extraction using the PCA method.

Figure 4 shows the block diagram for the PCA method. Similarly to the ICA method (see Figure 2), this diagram includes a pre-processing stage, fECG signal estimation as well as the evaluation of the results by comparing it with the data recorded by means of the FSE. The time shift between the estimated aECG^*^ and mECG^*^ components and amplitude difference was compensated using the same adaptive algorithm as in the previous case. Additionally, further morphological analysis (MA) is provided for both reference and estimated data.

The other main advantage of the PCA method is that we can find patterns in the data and then we can compress the data, i.e., by reducing the number of dimensions, without much loss of information. The input data vector is represented by the column vector $\text{x}={\left[x_{1},x_{2},\cdots \;,x_{n}\right]}^{\text{T}}$, with dimension *n*. We have a set of data that consists of *m* input vectors. The entire ensemble is compactly represented by the *n* × *m* data matrix **X** = [*x*_1_, *x*_2_, ⋯ , *x*_*m*_]. The purpose of PCA is to find those *m*(*m* < *n*) components of the elements of **X**, which reduce the dimensionality of the input vector in a mean squared error sense. One of the approaches to this solving problem is the projection of the data along orthogonal basis vectors through eigen decomposition of the covariance estimate of the input.

If we let **x** vector have a zero mean value, *E*{**x**} = 0, then the transformation of vector **x** can be defined as:

(9) $$\begin{array}{r} \text{s}={\text{x}}^{\text{T}}\text{U}={\text{U}}^{\text{T}}\text{x}. \end{array}$$

Or also

(10) $$\begin{array}{r} {\text{s}}_{i}={\text{x}}^{\text{T}}{\text{u}}_{i}={\text{U}}_{i}^{\text{T}}\text{x}, \end{array}$$

where matrix **U** = [*u*_1_, *u*_2_, ⋯ , *u*_*n*_] is the transformation matrix with the condition:

(11) $$\begin{array}{r} ||{\text{u}}_{i}||=\sqrt{{\text{u}}_{i}^{\text{T}}{\text{u}}_{i}}=1. \end{array}$$

That means that the module of the base vector **u**_*i*_ is equal to one. Equation (12) is also called the PCA data model. The value **s**_*i*_ is the projection of the input data **x** to the orthogonal base vectors **u**_*i*_. The vector $\text{s}={\left[s_{1},s_{2},\cdots \;,s_{n}\right]}^{\text{T}}$ is the principal component vector and the values **s**_*i*_ are the principal components.

We can also do the opposite approach. After PCA's main components **s**_*i*_ (i.e., U-matrix coefficients,) have been found, the data vector **x** can be reconstructed as follows:

(12) $$\begin{array}{r} \text{x}=\text{U}\text{s}=\overset{n}{\underset{i\;=\;1}{\sum }}{\text{s}}_{i}{\text{u}}_{i}. \end{array}$$

The aim of the PCA method is to find a linear orthogonal transformation represented by the **U** matrix so that the variance of **s**_*i*_ projection is maximum. The variance can be defined as:

(13) $$\begin{array}{r} \sigma_{s}^{2}=E\left\{{\text{s}}^{2}\right\}=E\left\{\left({\text{u}}^{\text{T}}\text{x}\right)\left({\text{x}}^{\text{T}}\text{u}\right)\right\}={\text{u}}^{\text{T}}\text{C}{\text{u}}_{i}, \end{array}$$

where **C** = *E*{**xx**^T^} = **C**^T^ is a symmetric covariance matrix of random vector **x**. From equation (14) it follows that the variance of the *s* projection is a function of the unit vector **u**_*i*_.

(14) $$\begin{array}{r} \psi \left(\text{u}\right)=\sigma_{s}^{2}={\text{u}}^{\text{T}}\text{C}\text{u}, \end{array}$$

where ψ(**u**) is the variance probe.

The aim of the PCA method is to find the base vectors **u**_*i*_ for which the variation probe ψ(**u**) is maximal under the condition expressed in Equation (12).

### 3.3. Principal components estimation

The problem about estimating the base vectors **u**_*i*_ is based on solving equation (16):

(15) $$\begin{array}{r} \text{C}{\text{u}}_{i}=\lambda_{i}{\text{u}}_{i};i=1,2,\cdots \;,n. \end{array}$$

The solution of the equation is to find the actual numbers λ_*i*_ (the numbers of the covariance matrix **C**) and the vectors of the covariance matrix **C**.

The **C** matrix's eigenvalues are ordered from the largest value to the smallest value λ_1_ > λ_2_ > ⋯ > λ_*n*_; λ_1_ = λ_*max*_ and by the ordered vectors **u**_*i*_ it is possible to create the matrix **U** = [**u**_1_, **u**_2_, ⋯ , **u**_*n*_], then the equation (15) can be expressed as:

(16) $$\begin{array}{r} \text{C}\cdot \text{U}=\text{U}\cdot \Lambda , \end{array}$$

where Λ denotes the diagonal matrix with the eigenvalues λ_1_, ⋯ , λ_*n*_. Respective column vectors *u*_*i*_ satisfy the orthogonality condition:

(17) $${\text{u}}_{i}^{\text{T}}\cdot {\text{u}}_{j}=\{\begin{array}{l} 1,i=j,\\ 0,i\neq j. \end{array}$$

That means that **U** is an identity matrix (15) and can be expressed as:

(18) $$\begin{array}{r} {\text{U}}^{\text{T}}\cdot \text{C}\cdot \text{U}=\text{U}\Lambda . \end{array}$$

By comparing equations (14) and (19) we can assume that:

(19) $$\begin{array}{r} \psi \left({\text{u}}_{i}\right)=\lambda_{i};\;i=1,2,\cdots \;,n. \end{array}$$

This means that the variance probes ψ(**u**_*i*_) seek the maximum variance and the matrix's eigenvalues are identical.

**Figure 5:**
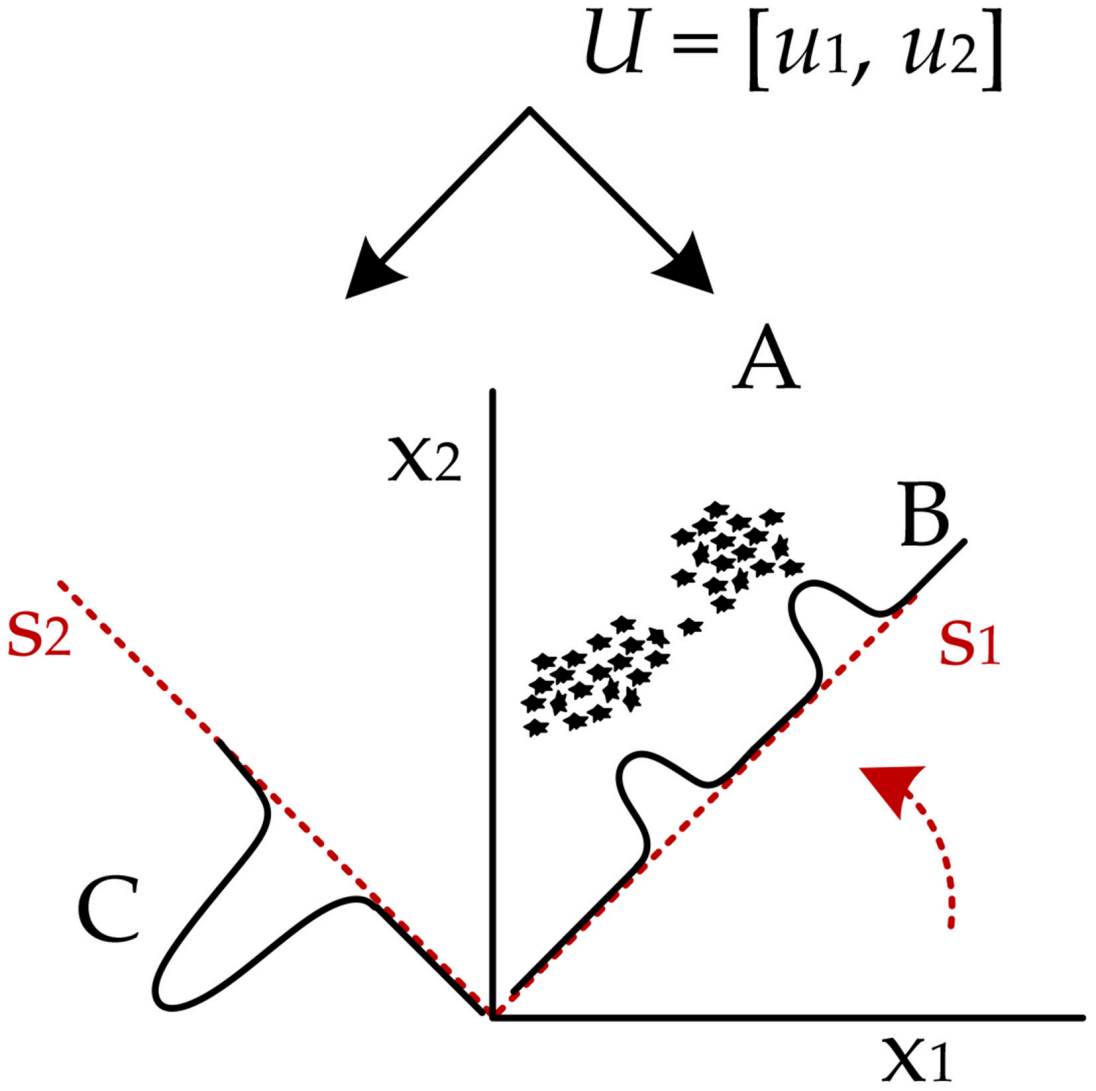
The principle of PCA method.

The PCA principle is illustrated in Figure 5 for the two-dimensional set of input data, depicted as points marked with the letter ‘A’. The data is expressed by the matrix **X**, $\text{X}={\left[x_{1i},x_{2i}\right]}^{\text{T}}$, *i* = 1, 2, ⋯ , *p*; where *p* is the number of points and *x*_*ji*_ is the *i*-th value of the *j*-random variable. On the horizontal axis, the values *x*_1_ are plotted, whereas *x*_2_ values are plotted on the vertical axis. The PCA method caused the rotation of the axes from the original position *x*_1_ and *x*_2_ to a position that is denoted by the letters *s*_1_ and *s*_2_. The axes rotated in the direction of the vectors of the covariance matrix. The rotation is represented by a dotted arrow in Figure 5. By projecting the dataset on an axis that is in the direction of the first vector, we capture the exact data structure that represents 2 clusters in this structure (“B”). The variation of this projection is the maximum of all possible directions (rotations). When projection is made to the *s*_2_ (“C”) axis, the information about 2 clusters is hidden.

## 4. Dataset

The research material consisted of multichannel fetal electrocardiograms obtained from 12 different women at ITAM in Zabrze, Poland during established labors, between 38 and 41 weeks of gestation and saved in 12 different recordings. Five of these recordings constitute the Abdominal and Direct Fetal Electrocardiogram Database (AD FECG) available to the public on the PhysioNet website (Matonia et al., 2006; Jezewski et al., 2012).

**Figure 6:**
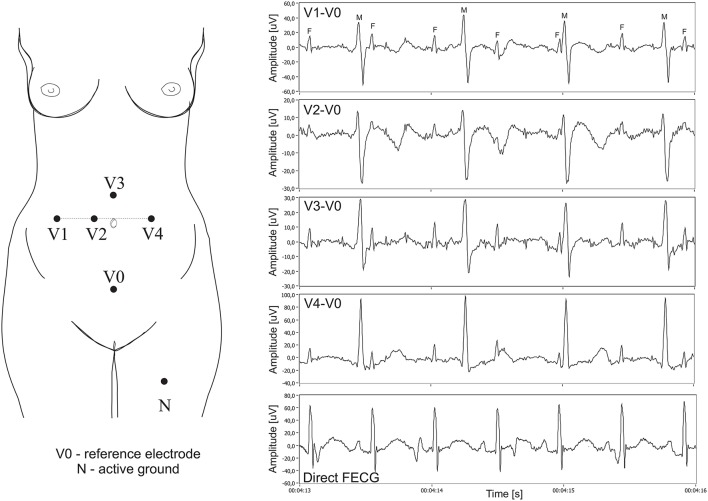
**Left**: Electrode placement system for recording and analysis of bioelectric signals acquired from the maternal abdominal wall, the direct electrode attached to the fetus head (sampling rate of 1 kHz, bandwidth 1 Hz–150 Hz and resolution of 16 bits). **Right:** Plots for 4 differential signals acquired from the maternal abdomen and the reference direct fECG signal (M, maternal QRS complexes; F, fetal QRS complexes).

In all cases the additional scalp electrode was placed for a clinical indication and all patients consented to participate in this study. The signals were recorded using a system for acquisition and analysis of fetal electrocardiogram KOMPOREL (ITAM Institute, Zabrze, Poland), in the Department of Obstetrics at the Medical University of Silesia. Each individual recording was comprised of signals acquired from four differential channels using electrodes placed on a maternal abdomen, and a direct electrocardiogram was registered from a fetal head as a reference signal (please see Figure 6).

The abdominal electrodes configuration was comprised of four electrodes placed around the navel, a reference electrode positioned above the pubic symphysis and a common mode reference electrode (with active-ground signal) placed on the left leg. To reduce the skin impedance, the areas under the Ag-AgCl electrodes (3M Red Dot 2271) were abraded (3M Red Dot Trace Prep 2236). It is important to emphasize that the acquisition of abdominal fECG signals during labor presents additional challenges we had to be aware of. Firstly, during labor the strongest signal was observed when the fetus was fully developed. On the other hand, the quality of abdominal signals acquired during labor was contaminated by considerable muscular activity of the uterus. Moreover, during signal acquisition the scalp electrode very often lost contact with the fetal head, causing temporary signal loss. Some fragments of the direct fECG signals were also distorted by interferences caused by maternal movements. Consequently, we had to select only those short fragments of the recordings for which there were no signal loss periods in the direct (reference) fECG signal. The acquisition of direct fetal electrocardiogram was carried out with a typical spiral electrode, commonly used in the direct fECG channel of popular bedside fetal monitors. The R-wave peaks were then automatically determined from the direct fECG signals by means of an on-line analysis algorithm applied in the KOMPOREL system. The accurate occurrence of these peaks was then verified (off-line) by a group of cardiologists, resulting in a set of reference markers precisely indicating the location of the R-waves. These markers were then stored together with the direct and indirect fECG signals. In 12 5-min recordings, a total of 5,165 maternal QRS complexes as well as 7,863 fetal QRS complexes were detected. Assuming the maximum width of the maternal QRS complex as 100 ms and that of the fetal as 40 ms, the number of complexes without feto-maternal coincidence was 2,415 maternal and 5,142 fetal.

### 4.1. Performance evaluation measures

**Figure 7:**
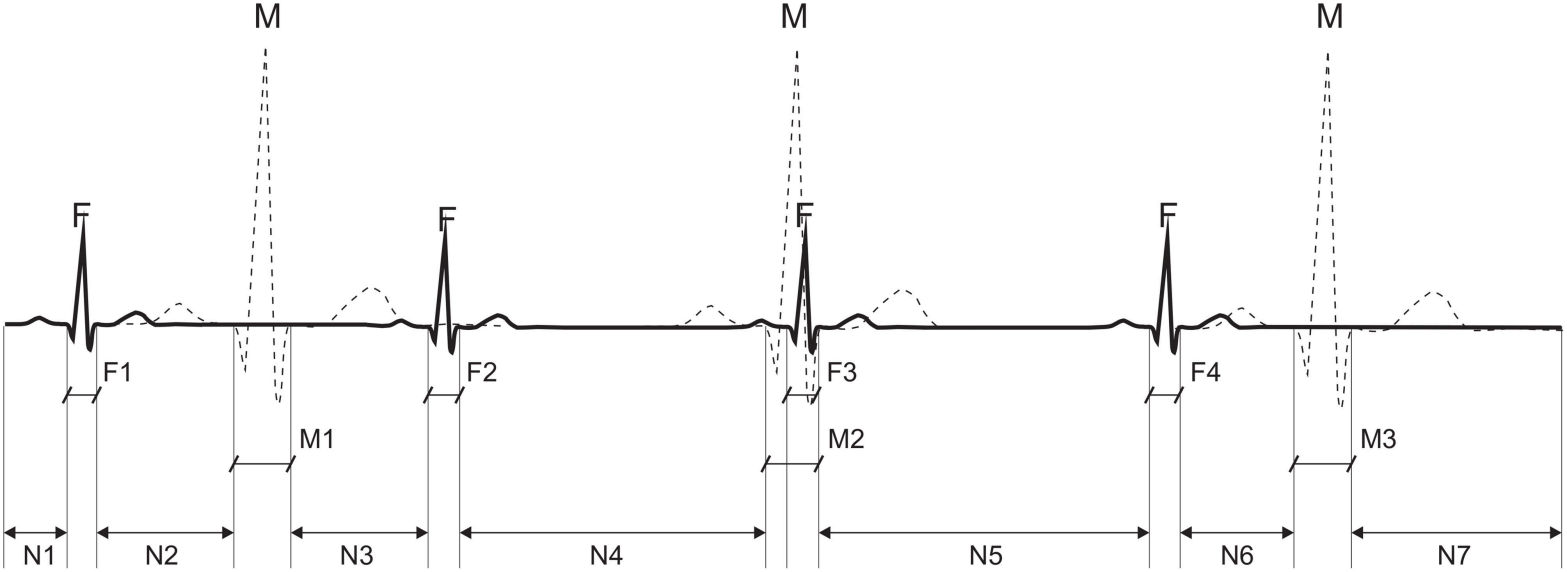
Calculation of an average power for particular components of the abdominal ECG signal: the average power of interference N is calculated from segments: N1 + N2 + N3 + N4 + N5 + N6 + N7, the maternal signal power M from M1 + M3, and fetal from: F1 + F2 + F4.

We used quantitative measures (dedicated evaluation indices) related to the Signal-to-Noise Ratio (SNR) to evaluate the sufficiency and diversity of the research data to clearly distinguish between useful signal components and contaminating interferences in the abdominal signals. These indices allow the evaluation of the mutual amplitude dependencies of maternal and fetal components on each other and their relation to other noise components in the aECG signals (Figure 7).

Assuming that the signal powers of P and T waves in the fetal and maternal complexes are negligible, the average power of interferences P_N_ in the abdominal signal can be presented as an average signal power outside the QRS complex locations given by:

(20) $$\begin{array}{r} {\text{P}}_{\text{N}}=\frac{\overset{L}{\underset{k\;=\;1}{\sum }}x^{2}\left(k\right)}{L}, \end{array}$$

where *L* is the number of samples in the abdominal signal *x*(*k*) that are not associated with any QRS complex.

Additionally, assuming that there is no correlation between the useful signal and the noise component, the average power of the maternal signal P_M_ is given by:

(21) $$\begin{array}{r} {\text{P}}_{\text{M}}=\frac{\overset{J}{\underset{i\;=\;1}{\sum }}\overset{\text{F}{\text{P}}_{\text{M}}\left(i\right)+{\text{S}}_{\text{M}}/2}{\underset{k=\text{F}{\text{P}}_{\text{M}}\left(i\right)-{\text{S}}_{\text{M}}/2}{\sum }}x^{2}\left(k\right)}{J\left({\text{S}}_{\text{M}}+1\right)}-{\text{P}}_{\text{N}}, \end{array}$$

where *J* is the number of detected maternal QRSs that do not overlap the fetal QRSs; S_M_ is the width of the maternal QRS (100 ms); and FP_M_(*i*) is the timing of the *i*-th maternal QRS occurrence.

Similarly, the average power of the fetal signal P_F_ is:

(22) $$\begin{array}{r} {\text{P}}_{\text{F}}=\frac{\overset{J}{\underset{i\;=\;1}{\sum }}\overset{\text{F}{\text{P}}_{\text{F}}\left(i\right)+{\text{S}}_{\text{F}}/2}{\underset{k=\text{F}{\text{P}}_{\text{F}}\left(i\right)-{\text{S}}_{\text{F}}/2}{\sum }}x^{2}\left(k\right)}{I\left({\text{S}}_{\text{F}}+1\right)}-{\text{P}}_{\text{N}}, \end{array}$$

where *I* is the number of detected fetal QRSs that do not overlap the maternal ones, S_F_ is the fetal QRS width (40 ms), and FP_F_(*i*) is the timing of the *i*-th fetal QRS occurrence.

Based on these values, the WMF index relating the maternal and fetal components can be defined as follows:

(23) $$\begin{array}{r} \text{WMF}=10\log\frac{{\text{P}}_{\text{M}}}{{\text{P}}_{\text{F}}}, \end{array}$$

and similarly, the WM and WF indices describing the mECG signal and fECG signal powers in relation to the power of the interfering components, can be respectively defined as follows:

(24) $$\begin{array}{r} \text{WM}=\text{SN}{\text{R}}_{\text{MECG}}=10\log\frac{{\text{P}}_{\text{M}}}{{\text{P}}_{\text{N}}}, \end{array}$$

(25) $$\begin{array}{r} \text{WF}=\text{SN}{\text{R}}_{\text{FECG}}=10\log\frac{{\text{P}}_{\text{F}}}{{\text{P}}_{\text{N}}}. \end{array}$$

From the analysis of these indices based on the results obtained from the four abdominal signal channels, it could clearly be seen that for the research data used, the dominant signal was the mECG, whose amplitude was always more than three times higher than that of the noise component signal (mean WM is 10.9 dB, with a range from 5 to 12.6 dB). Similarly, its relation to the fetal signal, as indicated by the WMF index, exceeded 7 dB on average (range from 1.8 to 11.9 dB). The WF indicator, informing the relationship between fetal QRSs and the noise component, varied very considerably. Its average value was 3.9 dB, but there were cases where the WF oscillated within almost 7 dB (the fetal QRS amplitude was more than twice as high as that of the noise component), and in cases when WF was close to only 0.5 dB the fetal QRS level was comparable to the amplitude of interferences.

## 5. Results

### 5.1. Fetal heart rate determination

In this section, we present our evaluation results of the fECG signals extracted from aECG signals acquired by using 4 abdominal electrodes. The fECG signals acquired by means of the Fetal Scalp Electrode (FSE) were used as reference. This direct signal was used for the verification of the extracted signal by comparing the fHR values determined from each of them.

In the reference and extracted fECG signals, the R waves were detected and the intervals between subsequent peaks (RR intervals) were determined. Fetal HR was then calculated as the number of the RR intervals in 1 min.

**Figure 8:**
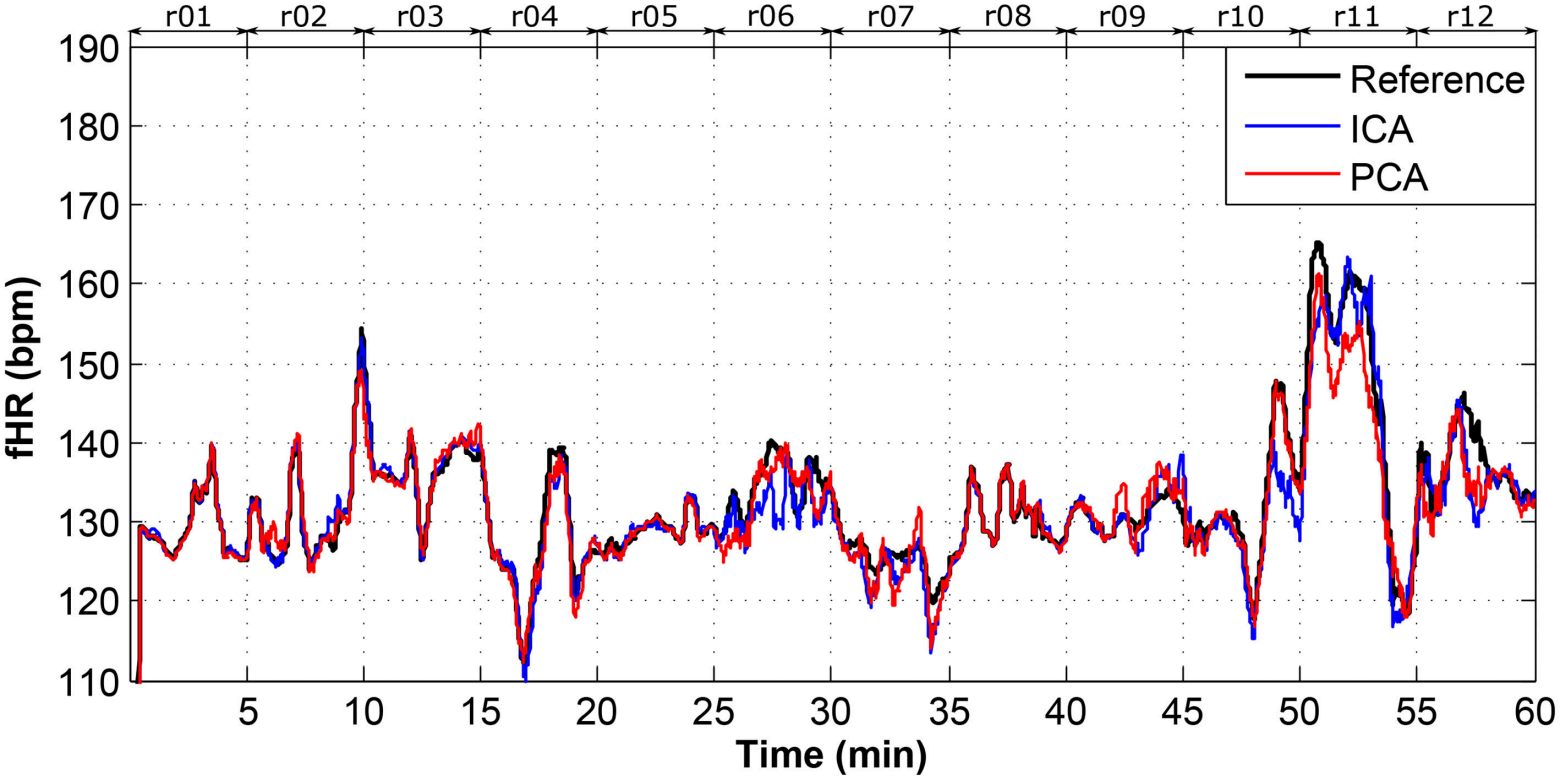
Dataset overview of real01 to real12 fHR determination.

Figure 8 presents an overview of the dataset used in fHR determination. It includes the mean fHR waveform calculated for all of the available data. This figure shows that 60 min of ECG data (i.e., around 8,000 R waves) in total were available for our experiments. This dataset covers different gestation ages, fetal positions, maternal body mass indexes (BMI), etc. Such a unique heterogeneous dataset with sufficient length combining continuous FSE and abdominal ECG recordings allows for robust testing of the applied ICA and PCA methods.

**Figure 9:**
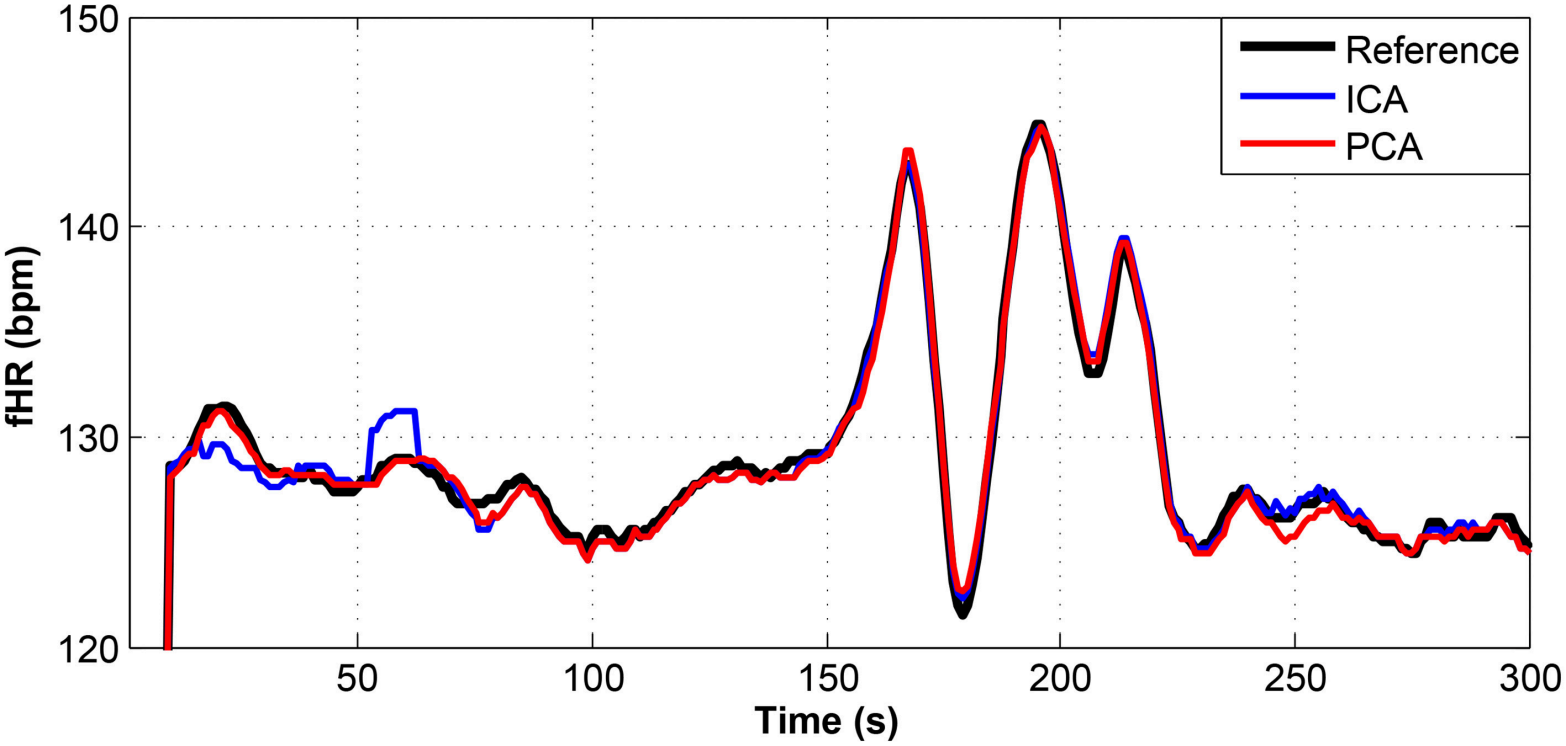
Comparison of the fHR values determined from the fECG signals extracted from recording real01 by using the ICA and PCA methods with the reference fECG signal recorded by means of the FSE.

**Figure 10:**
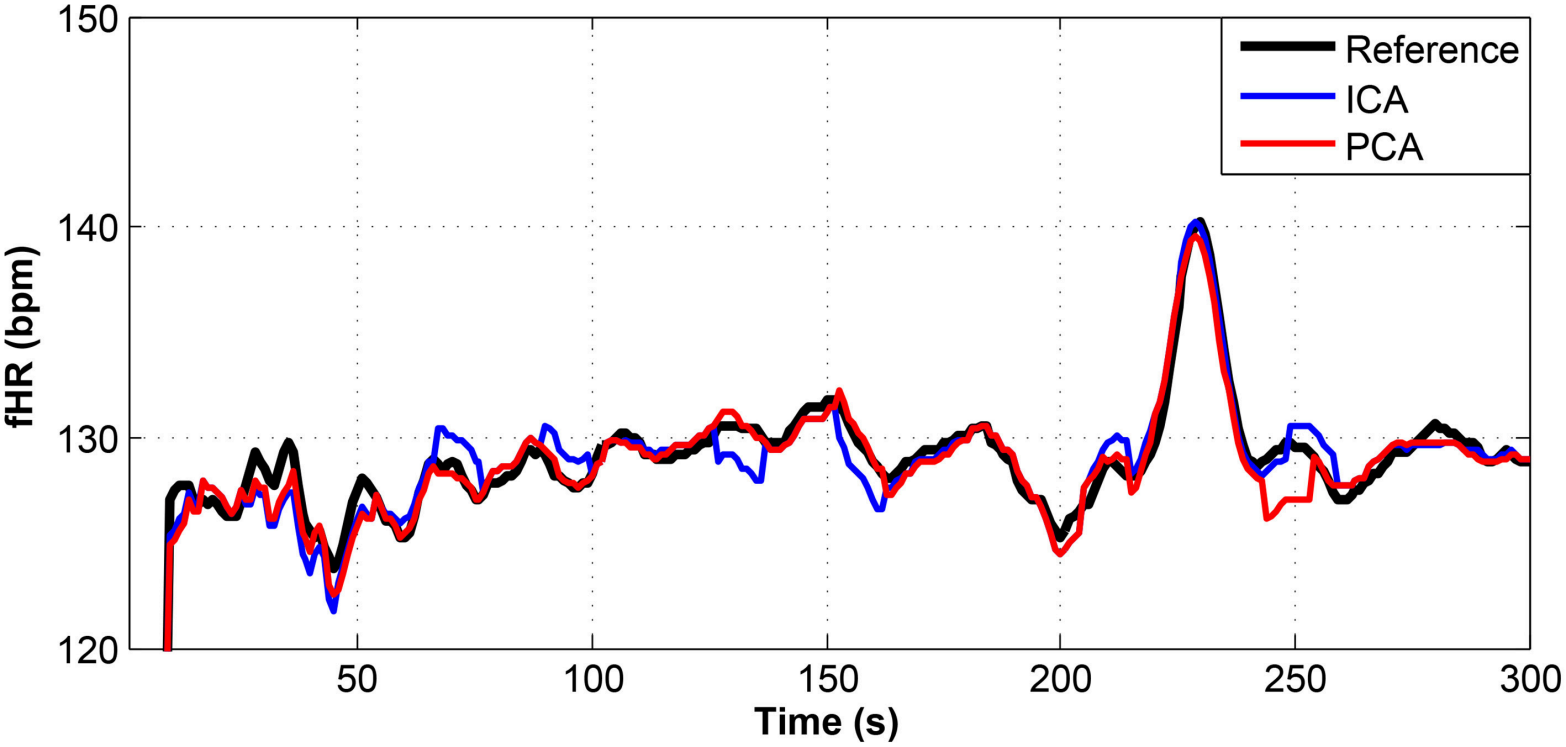
Comparison of the fHR values determined from the fECG signals extracted from recording real05 by using the ICA and PCA methods with the reference fECG signal recorded by means of the FSE.

**Figure 11:**
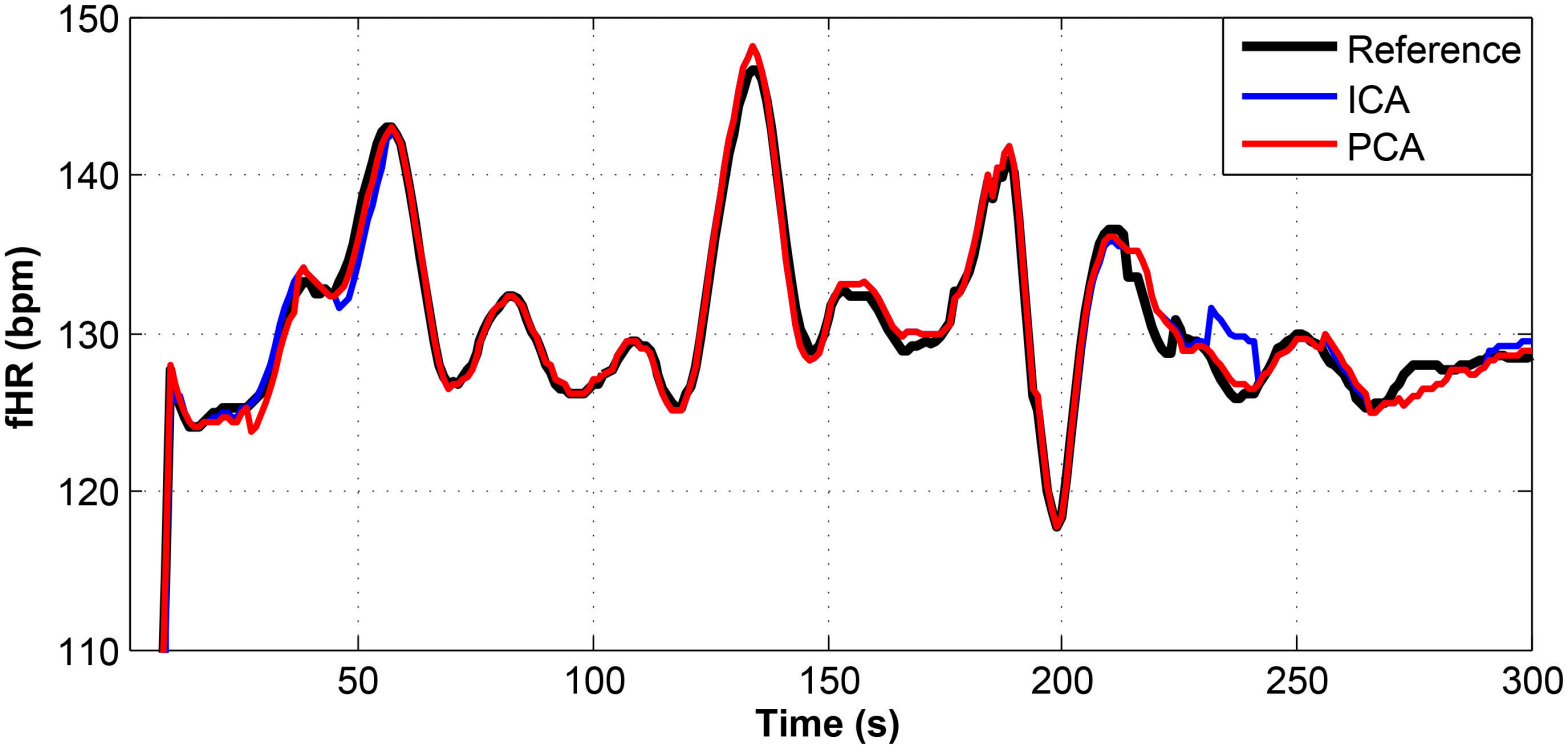
Comparison of the fHR values determined from the fECG signals extracted from recording real08 by using the ICA and PCA methods with the reference fECG signal recorded by means of the FSE.

**Figure 12:**
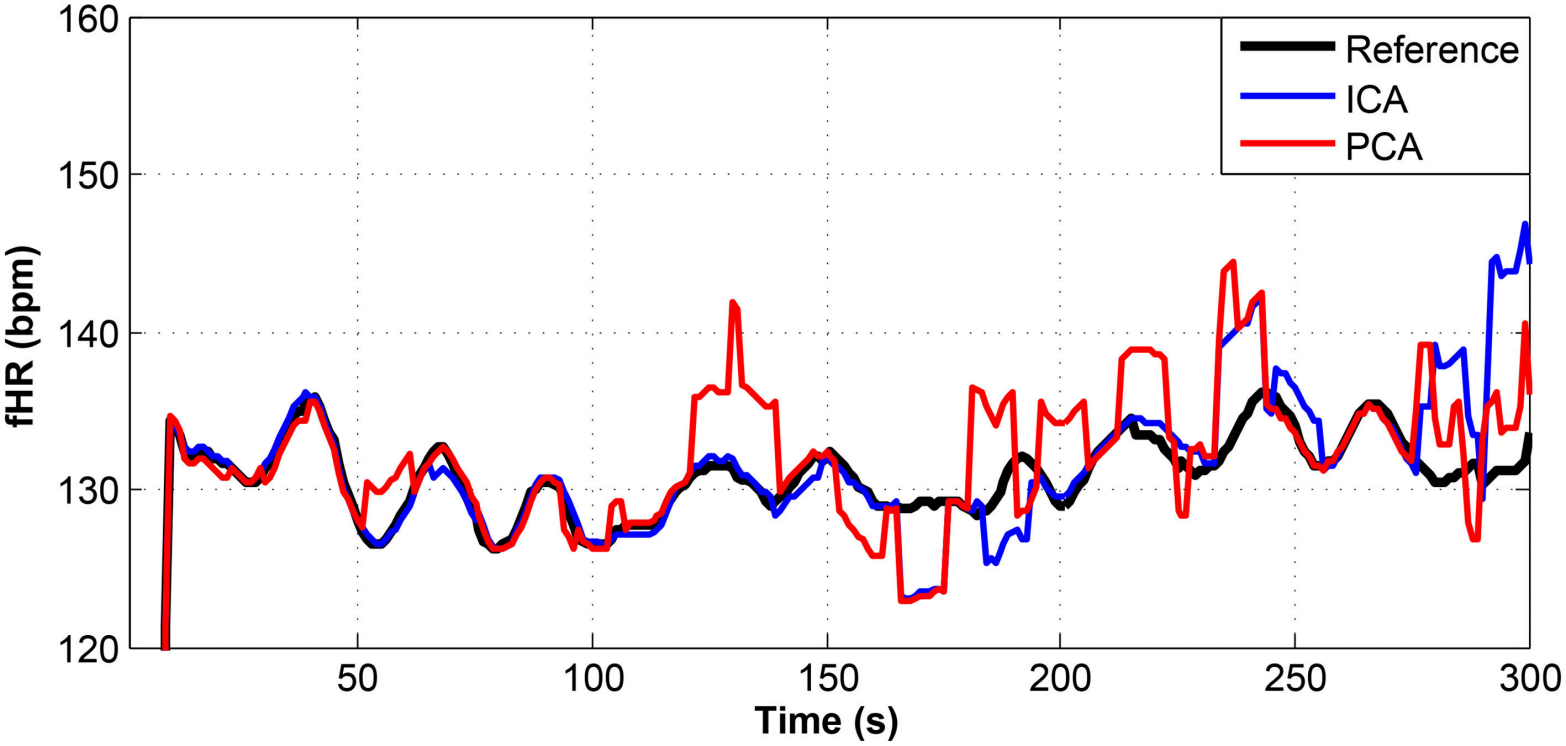
Comparison of the fHR values determined from the fECG signals extracted from recording real09 by using the ICA and PCA methods with the reference fECG signal recorded by means of the FSE.

**Figure 13:**
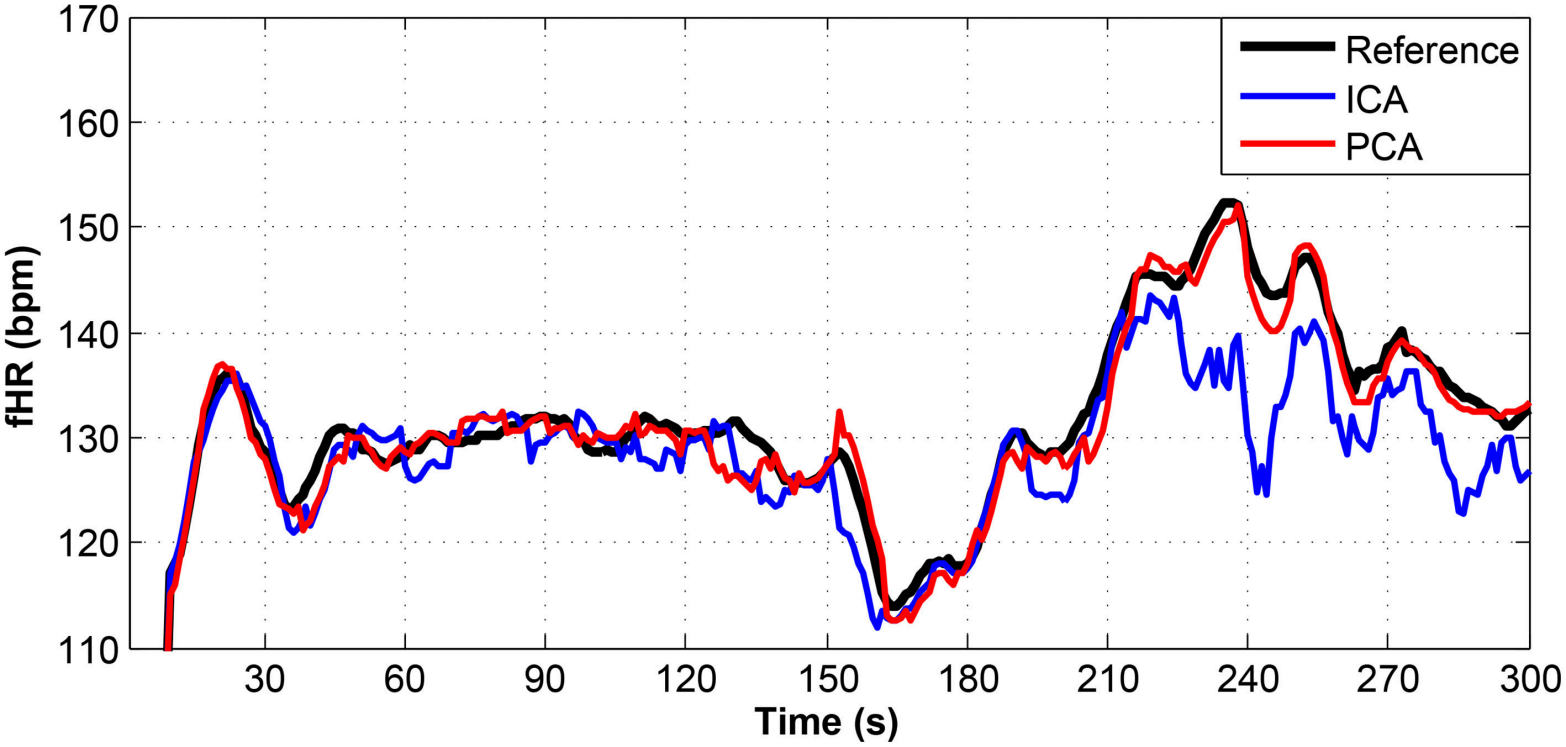
Comparison of the fHR values determined from the fECG signals extracted from recording real10 by using the ICA and PCA methods with the reference fECG signal recorded by means of the FSE.

**Figure 14:**
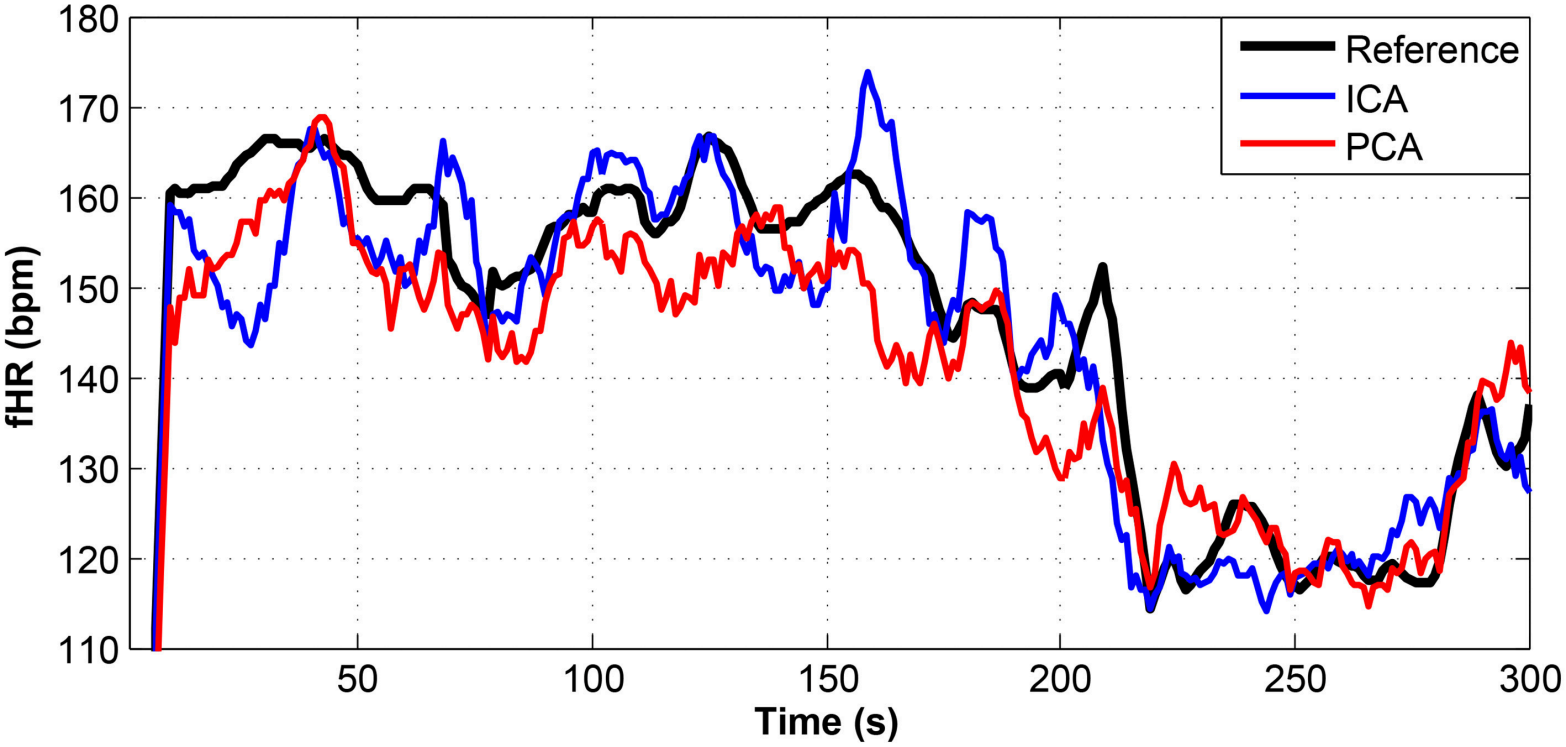
Comparison of the fHR values determined from the fECG signals extracted from recording real11 by using the ICA and PCA methods with the reference fECG signal recorded by means of the FSE.

In this study, we used two types of moving average filters (MAF). For the dataset overview depicted in Figure 8, the most suitable values for the MAF window size was 30 as it produced the best visual results and enhanced the fHR waveform trend. However, for the rest of the determined fHR waveforms, we used a MAF window size of 10 since the data length was significantly lower (5 min). The following figures (Figures 9–**14**) show the details of the fHR waveforms shown in Figures 8, 9–11, which reveal good agreement between the fHR values determined from the reference fECG signals and those calculated by applying the ICA and PCA methods to the aECG signals (recordings real01, real05, real08) to extract fECG signals, whereas Figures 12–14 show fragments with sufficient quality for fHR detection (recording real09, real10, real11), however, these fragments were insufficient for advanced morphological fECG signal analysis.

Figures 9–11 show the comparison of the fHR values (in bpm) determined from the fECG signals extracted from recordings real01 and real05, real08, respectively, as well as those calculated from the direct or reference fECG signals. We can observe that the fHR values determined from the extracted signals (especially those by the PCA method) were comparable to those calculated from the reference signal (acquired by means of the FSE).

For other recordings, however, the results were different. In the case of the real09, real10, and real11 recordings (Figures 12–14, respectively), the fHR values determined from the fECG signals extracted by the ICA method significantly differed from those fHR values calculated by using the reference fECG signals. It was observed that the fHR values determined by the PCA method were significantly more accurate than those determined by the ICA method. This could be caused by several factors that will be discussed in more detail (see section 6.4).

### 5.2. Statistical analysis

In this section, we present the statistical evaluation of the accuracy of the ICA and PCA methods in the extraction of fECG signals compared to the reference fECG signal measured by means of the FSE. We use the following metrics and equations (26–29) to achieve this task: Sensitivity (Se), Positive Predictive Value (PPV), Accuracy (ACC), and F1 (the overall probability that the fQRS complex is correctly detected) (Samuel et al., 2017). In these equations, TP represents True Positive (correct detection of fQRS complexes when they are present in the fECG signals), FN stands for False Negative (incorrect detection of fQRS complexes when they are not present in the fECG signal), FP symbolizes False Positive (incorrectly indicates the presence of fQRS complexes in the fECG signals), and TN (true detection of the absence of fQRS complexes in the fECG signals). Tables 1, 2 show the calculated values of these parameters for the analyzed records. For the detection of the fQRS complexes, we used an extended version of the Pan and Tompkins algorithm (Pan and Tompkins, 1985); and the accuracy of the fECG estimation was evaluated by a beat-to-beat comparison of the extracted fQRS complexes by means of the ICA or PCA methods and the reference fQRS complexes detected in the reference fECG signal acquired by the FSE (Zhang et al., 2017).

(26) $$\begin{array}{r} \text{Se}=\frac{\text{TP}}{\text{TP}+\text{FN}}\cdot 100. \end{array}$$

(27) $$\begin{array}{r} \text{PPV}=\frac{\text{TP}}{\text{TP}+\text{FP}}\cdot 100. \end{array}$$

(28) $$\begin{array}{r} \text{ACC}=\frac{\text{TP}}{\text{TP}+\text{FP}+\text{FN}}\cdot 100. \end{array}$$

(29) $$\begin{array}{r} \text{F}1=2\cdot \frac{\text{PPV}\cdot \text{Se}}{\text{PPV}+\text{Se}}=\frac{2\cdot \text{TP}}{2\cdot \text{TP}+\text{FP}+\text{FN}}\cdot 100. \end{array}$$

**Table 1:** Statistical evaluation of fQRS detection by ICA method.

**Recordings**	**TP**	**FP**	**FN**	**± 1.96 SD**	**Se (%)**	**PPV (%)**	**ACC (%)**	**F1 (%)**
real01	644	2	3	98.33	99.54	99.69	99.23	99.61
real02	663	9	5	95.33	99.25	98.66	97.93	98.96
real03	686	14	7	93.00	98.99	98.00	97.03	98.49
real04	643	11	19	90.00	97.13	98.32	95.54	97.72
real05	645	5	7	96.00	98.93	99.23	98.17	99.08
real06	676	12	15	91.00	97.83	98.26	96.16	98.04
real07	626	6	24	90.00	96.31	99.05	95.43	97.66
real08	651	4	3	97.67	99.54	99.39	98.94	99.47
real09	657	5	4	97.00	99.39	99.24	98.65	99.32
real10	635	3	24	91.00	96.36	99.53	95.92	97.92
real11	719	10	17	91.00	97.69	98.63	96.38	98.16
real12	688	10	15	91.67	97.87	98.57	96.49	98.22
Total	7,933	91	143	93.50	98.23	98.87	97.13	98.55

**Table 2:** Statistical evaluation of fQRS detection by PCA method.

**Recordings**	**TP**	**FP**	**FN**	**± 1.96 SD**	**Se (%)**	**PPV (%)**	**ACC (%)**	**F1 (%)**
real01	644	1	2	99.00	99.69	99.84	99.54	99.77
real02	663	14	13	91.00	98.08	97.93	96.09	98.00
real03	686	14	9	92.33	98.71	98.00	96.76	98.35
real04	643	12	17	90.33	97.42	98.17	95.68	97.79
real05	645	3	8	96.33	98.77	99.54	98.32	99.15
real06	676	9	16	91.67	97.69	98.69	96.43	98.18
real07	626	10	17	91.00	97.36	98.43	95.87	97.89
real08	651	2	2	98.67	99.69	99.69	99.39	99.69
real09	657	13	6	93.67	99.10	98.06	97.19	98.57
real10	635	6	14	93.33	97.84	99.06	96.95	98.45
real11	719	3	21	92.00	97.16	99.58	96.77	98.36
real12	688	5	16	93.00	97.73	99.28	97.04	98.50
Total	7,933	92	141	93.53	98.27	98.86	97.17	98.56

### 5.3. Bland-altman statistical analysis

For further evaluation of the accuracy of the determined fHR values, the Bland-Altman method was utilized. This method is based on calculating the mean difference between two methods of measurement (so-called bias), and 95 % of the limits of agreement as the mean difference (2 SD), or more precisely, (1.96 SD). It is expected that the chosen limit of 95 % contains 95 % of the variances between the two tested methods: the reference and estimated fHR values in our case. Therefore, the fHR values determined from the extracted fECG signals are assumed to be accurate, such that 95% of all of them fall in the range of ± 1.96 SD (Myles and Cui, 2007).

**Figure 15:**
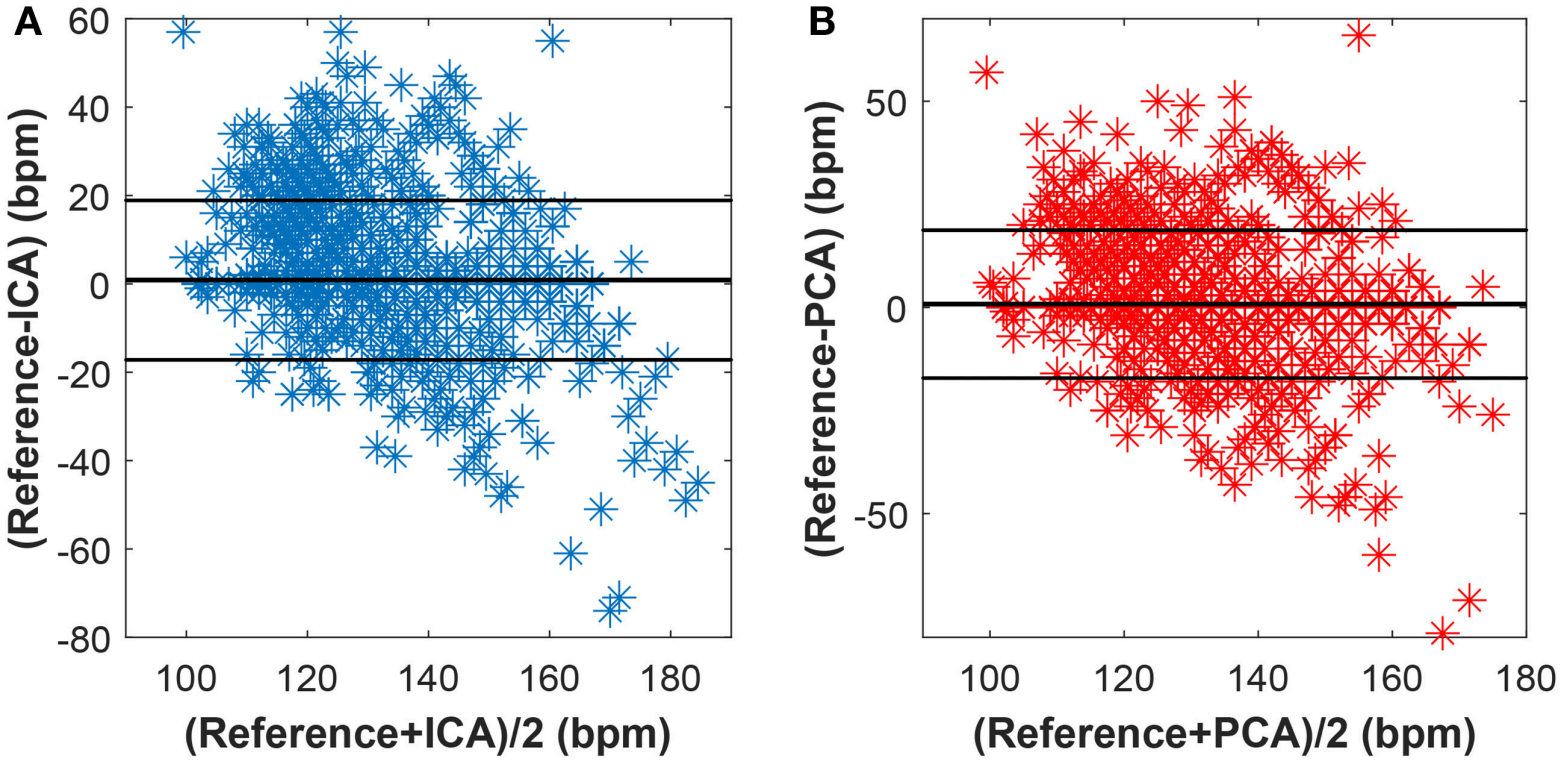
Bland-Altman plot for the entire dataset using **(A)** the ICA and **(B)** the PCA methods.

**Figure 16:**
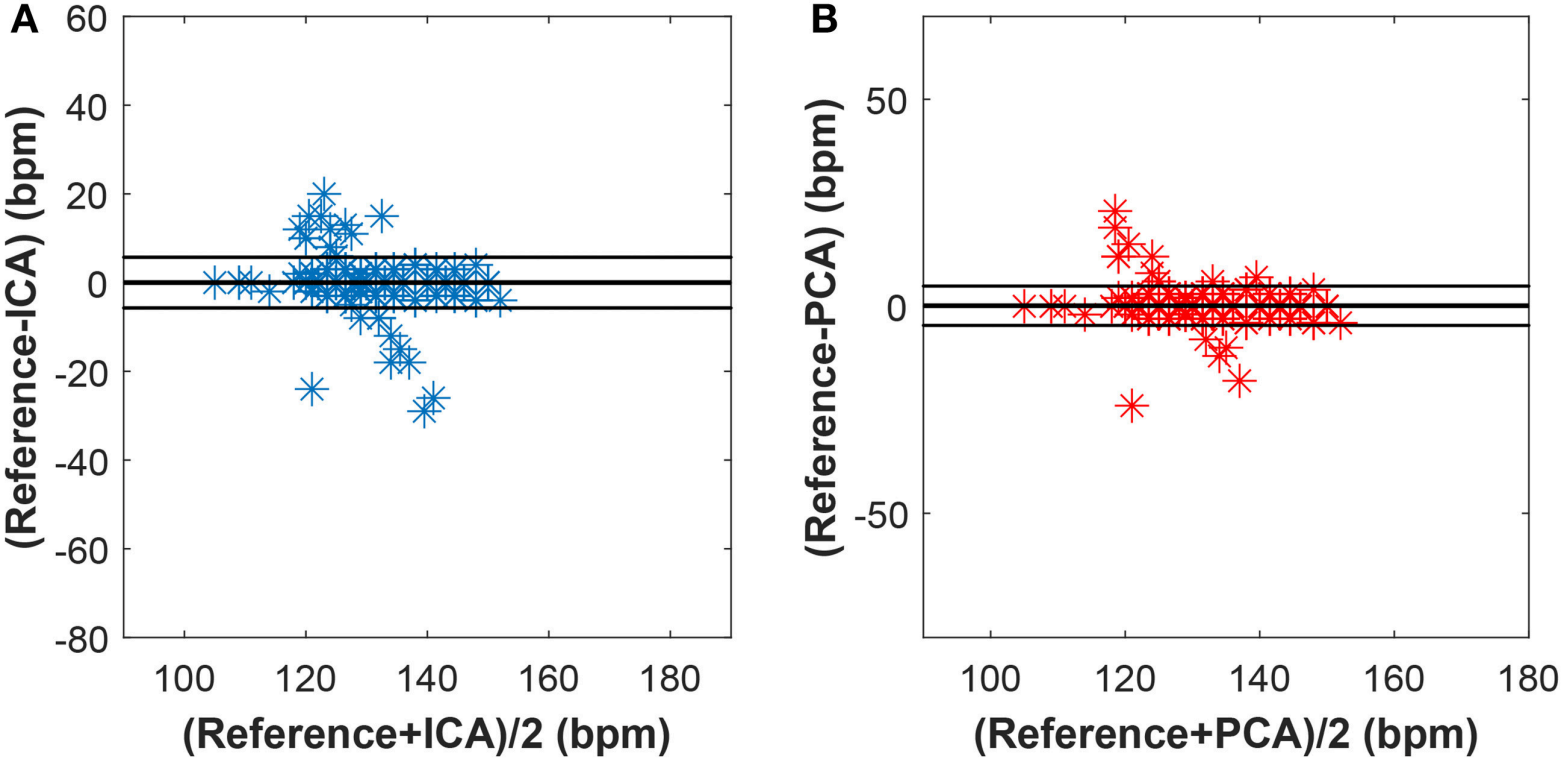
Bland-Altman plot for recordings real01, real05, and real08 using **(A)** the ICA and **(B)** the PCA methods.

**Figure 17:**
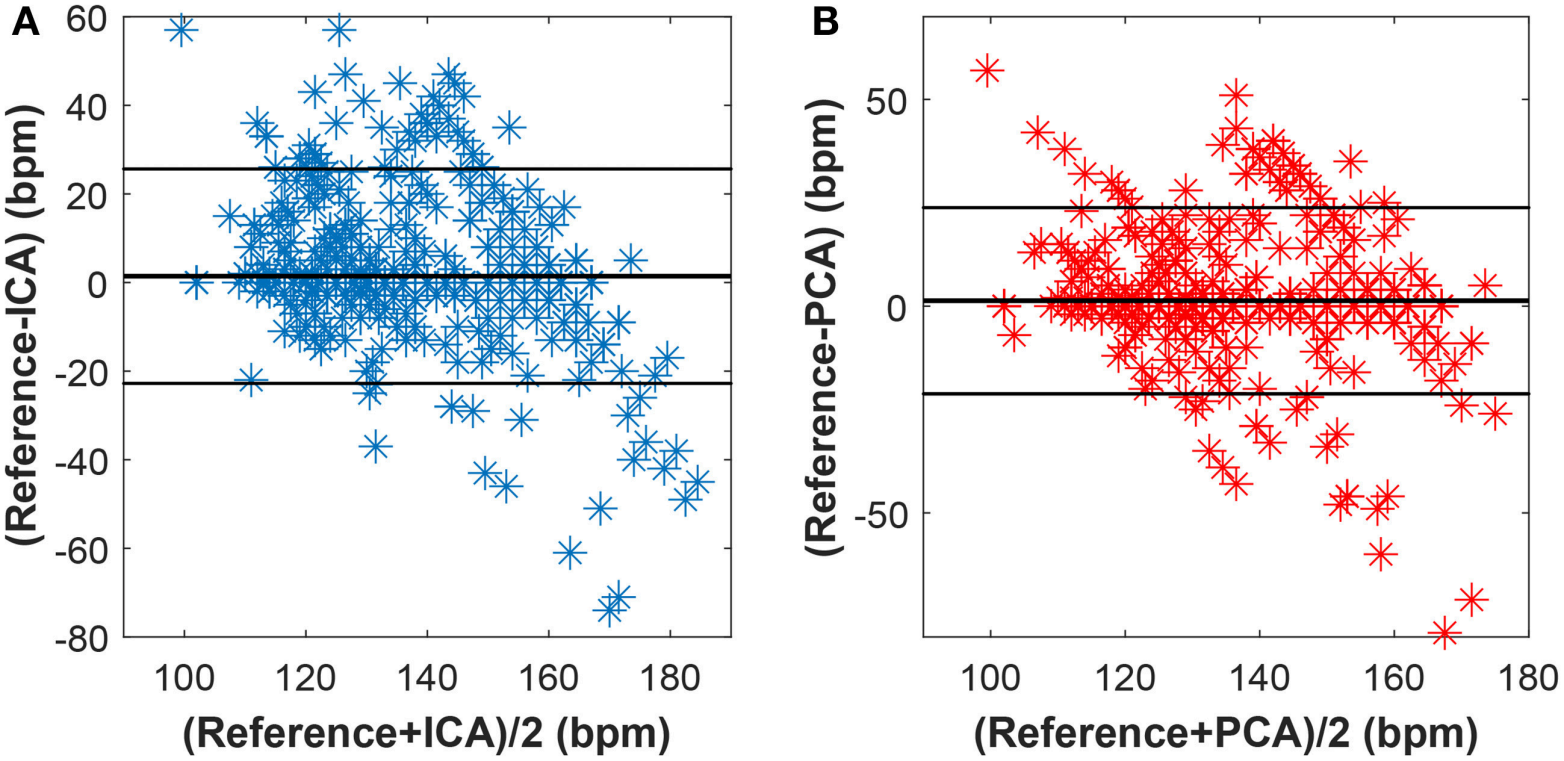
Bland-Altman plot for recordings real09, real10, and real11 using **(A)** the ICA and **(B)** the PCA methods.

Figures 15–17 show the plots based on the Bland-Altman statistical analysis technique for both methods with 12 different data recordings. In these plots, there are 3 lines: 2 of them indicate the chosen limit of 95%, whereas the 3rd one is bold and denotes the state when the signals match. The closer to zero the results are, the better correlation between the fHR determined from the reference signal and the one determined from the signals extracted by the PCA or ICA methods.

Figure 15 shows the Bland-Altman plot of whole dataset depicted in Figure 8. The results show that these methods are effective for recordings real01, real05, and real08 as only for these recordings the value of SD exceeded 95% (see Tables 1, 2). Most of the data are thus highly uncorrelated in the Bland-Altman plot. That is caused by the unsatisfactory results achieved by some of the recordings as discussed above.

The results in Figure 16 show a good correlation in case of recordings real01, real05, and real08, whereas recordings real09, real10, and real11 are highly uncorrelated (see Figure 17). This further confirms the results included in Tables 1, 2.

### 5.4. Challenge 2013 results comparison

To present a better implementation of the aforementioned techniques, we compared the performance of our approach to competitive algorithms available in literature. We tested the algorithms on the open source Fetal ECG database on Physionet.org (set A, see phy, 2014), and compared the performance in fECG extraction with the results that were presented at the Computing in Cardiology Conference in 2013, summarizing the competition “Noninvasive Fetal ECG Challenge.” Since HRMSE and RRRMSE Scoring parameters for the effectiveness evaluation proposed in the competition turned out to be too controversial, we primarily focused on the comparison of PPV, Se, ACC, and F1 index (see Table 3).

**Table 3:** Performance of the tested algorithms on set a.

**Method**	**± 1.96 SD**	**Se (%)**	**PPV (%)**	**ACC (%)**	**F1 (%)**
ICA	79.71	84.45	99.71	84.26	91.16
PCA	75.98	82.05	99.45	81.68	89.63

Behar et al. presented the possibility of using ICA and PCA (with various combinations of these methods) for the extraction of the fECG signal (Behar et al., 2013, 2014). The results showed that the algorithms used in combination with other algorithms perform significantly better than if used separately. Our adaptive algorithm was able to enhance the quality of the fECG extraction compared to the results of ICA and PCA being used separately (improvement of approximately 27 and 38% in terms of F1 for ICA and PCA, respectively).

### 5.5. Graphical interpretation of the results

**Figure 18:**
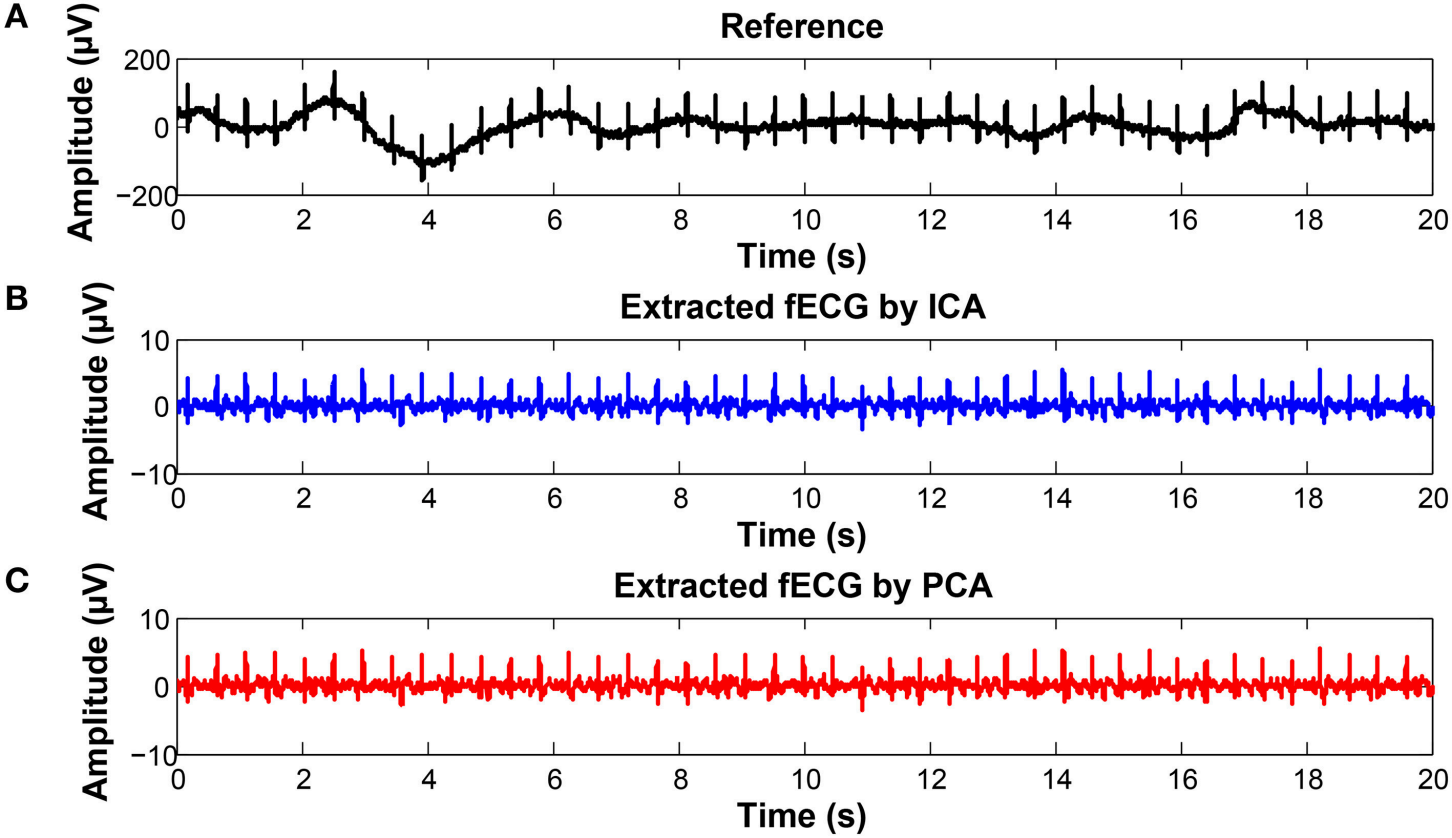
Examples of the signals from recording real10 used for comparison: **(A)** Reference (ideal) fECG signal recorded by means of the FSE, **(B)** fECG signal extracted by the ICA method,**(C)**fECGsignal extracted by the PCA method.

**Figure 19:**
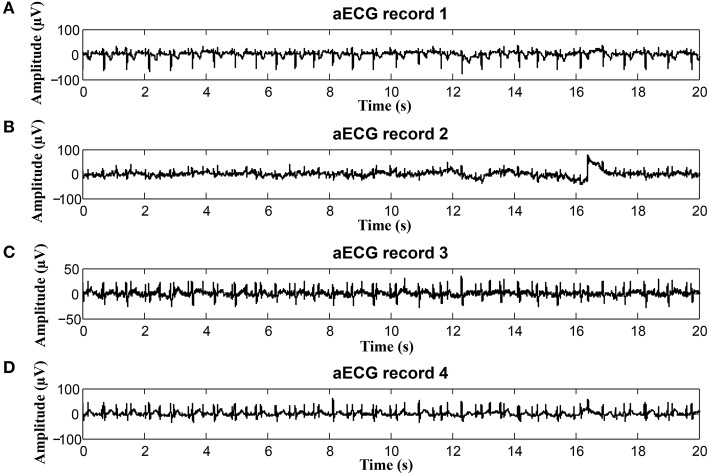
Example of a high quality input recordings (real01) **(A)** abdominal record1; **(B)** abdominal record2; **(C)** abdominal record3; **(D)** abdominal record4.

To keep our paper to a reasonable length, in this part, we provide the graphical interpretation of the some of the output signals as examples to facilitate their visual evaluation and discuss the unsatisfactory results achieved for some of the recordings. Figure 18 includes the reference signal recorded by means of the FSE (Figure 18A), and the fECG signals extracted by the ICA (Figure 18B) and PCA (Figure 18C) methods. These results show that the maternal R waves were successfully suppressed while fetal R waves remained unchanged. This is a vital requirement for accurate fHR detection. In terms of the estimated fECG signal morphology, the shape and the duration of P waves were unchanged. However, S and Q waves were deformed (lowered) by the applied signal processing methods. It is important to note that these satisfactory results were mainly achieved due to the high quality of the input ECG signal recordings (see Figure 19).

**Figure 20:**
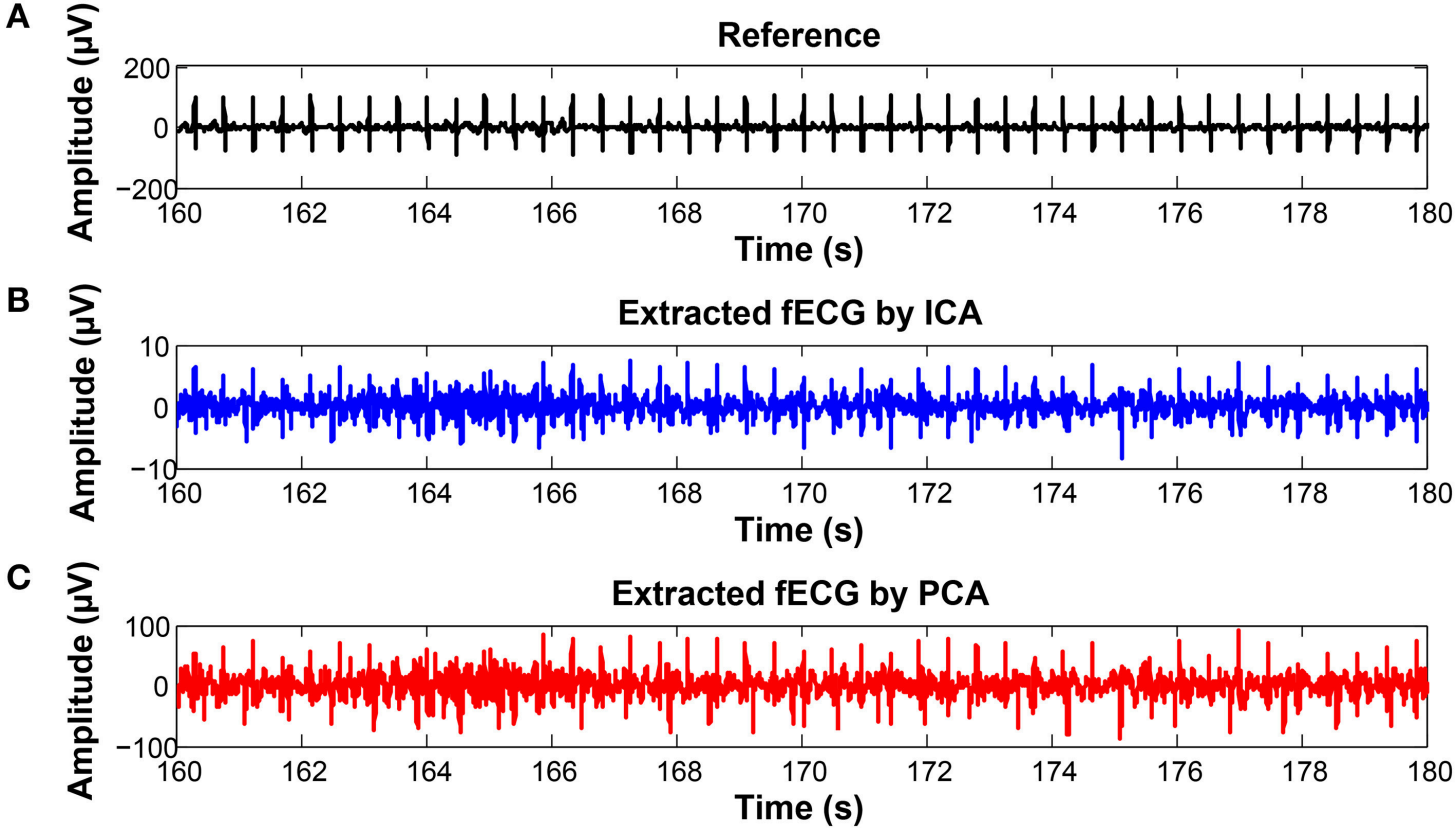
Examples of the signals producing unsatisfactory results in fHR determinantion from recording realX: **(A)** Reference (ideal) fECG signal recorded by means of the FSE, **(B)** fECG signal extracted by the ICA method, **(C)** fECG signal extracted by the PCA method.

**Figure 21:**
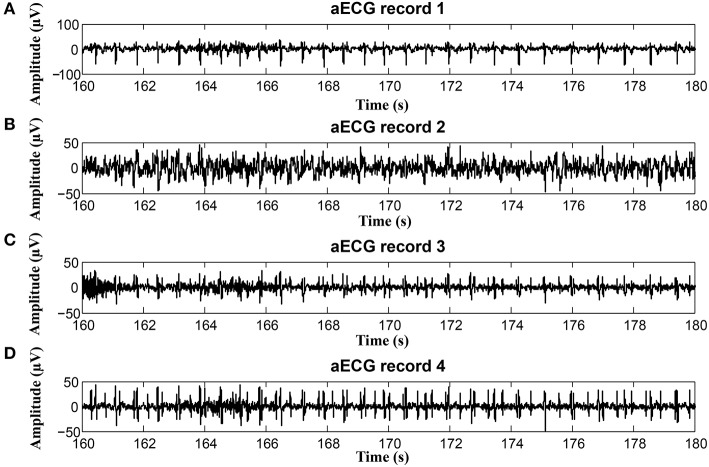
Examples of input signals for the fHR waveform interval with values lower than the reference: **(A)** abdominal record1; **(B)** abdominal record2; **(C)** abdominal record3; **(D)** abdominal record4.

On the contrary, in some records, the determined fHR was lower than the fHR calculated from the reference signal. Figure 20 (input data on Figure 21) presents some examples of the signals that produced unsatisfactory results. It is noticeable that some of the fetal R peaks were suppressed, which could subsequently lead into missed R-peak detection and thus a decreased value of the fHR.

**Figure 22:**
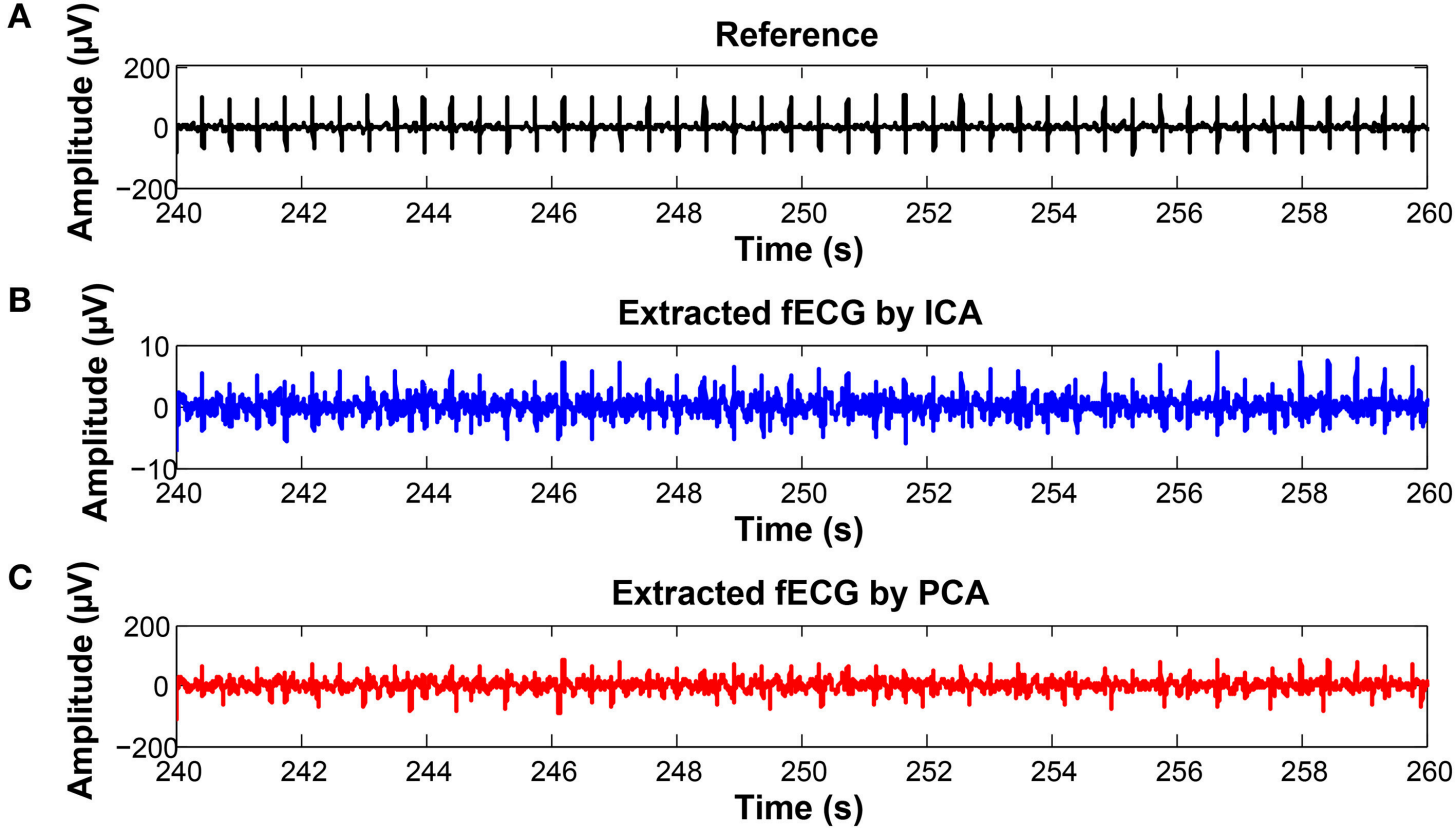
Examples of the signals producing unsatisfactory results in fHR determinantion from recording realX: **(A)** Reference (ideal) fECG signal recorded by means of the FSE, **(B)** fECG signal extracted by the ICA method, **(C)** fECG signal extracted by the PCA method.

**Figure 23:**
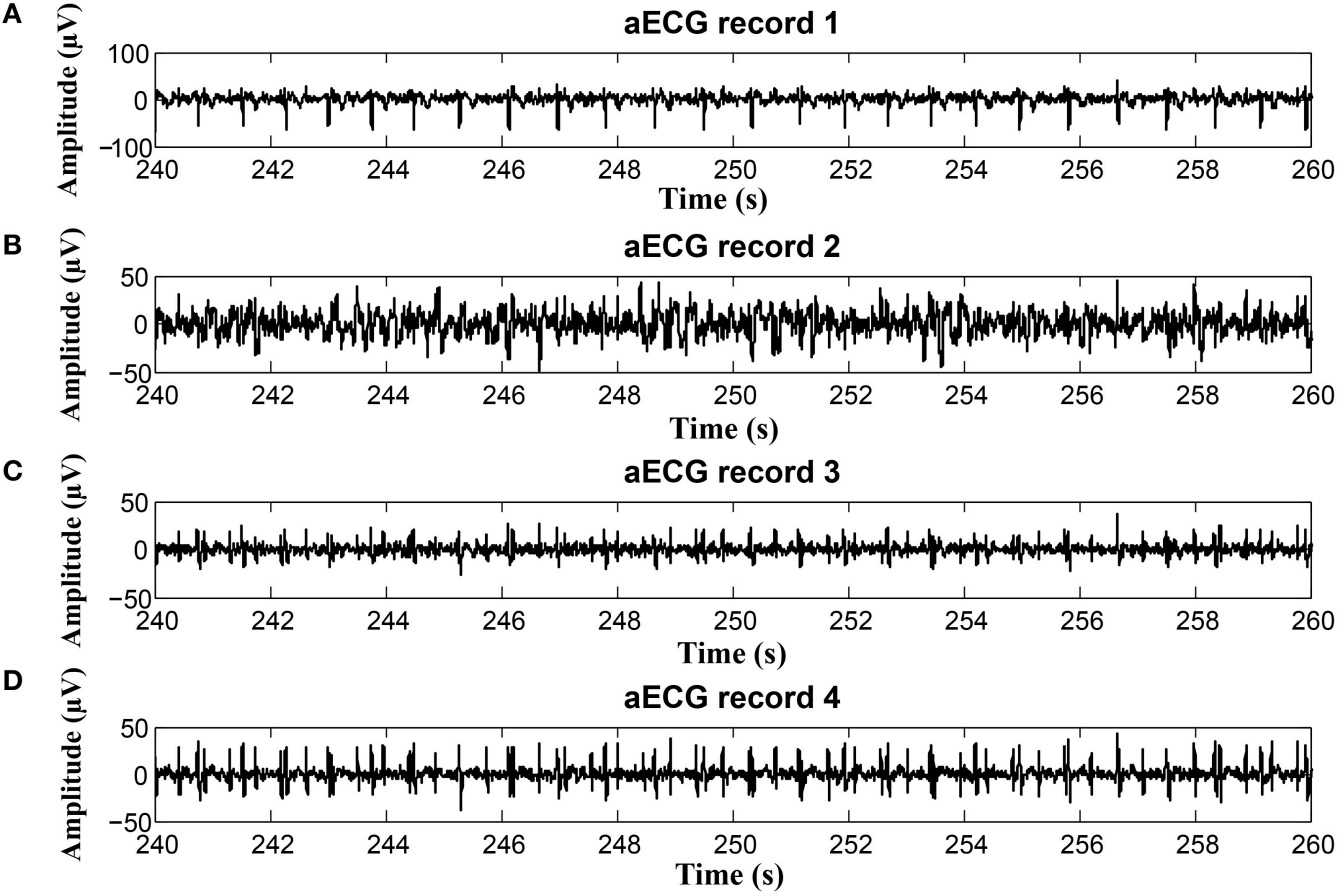
Examples of input signals for the fHR waveform interval with values exceeding the reference: **(A)** abdominal record1; **(B)** abdominal record2; **(C)** abdominal record3; **(D)** abdominal record4.

In some time intervals, the fHR waveforms determined from the signals estimated by PCA and ICA methods exceeded the waveform of the fHR determined from the reference signal recorded by means of FSE (see Figure 12). An analysis of the estimated signals in these particular fragments reveals maternal residues (see Figure 22). These artifacts produce false positively detected fetal peaks and thus increase the mean fHR value. One of the reasons that the PCA and ICA methods performed worst can be noticed in Figure 23, which includes the input data in the same time intervals. The quality of the recordings is significantly lower than in the case of the signals depicted in Figure 19, especially in the case of abdominal signals called record 2 where the motion artifacts distorted the signal.

### 5.6. Non-invasive ST-analysis

**Figure 24:**
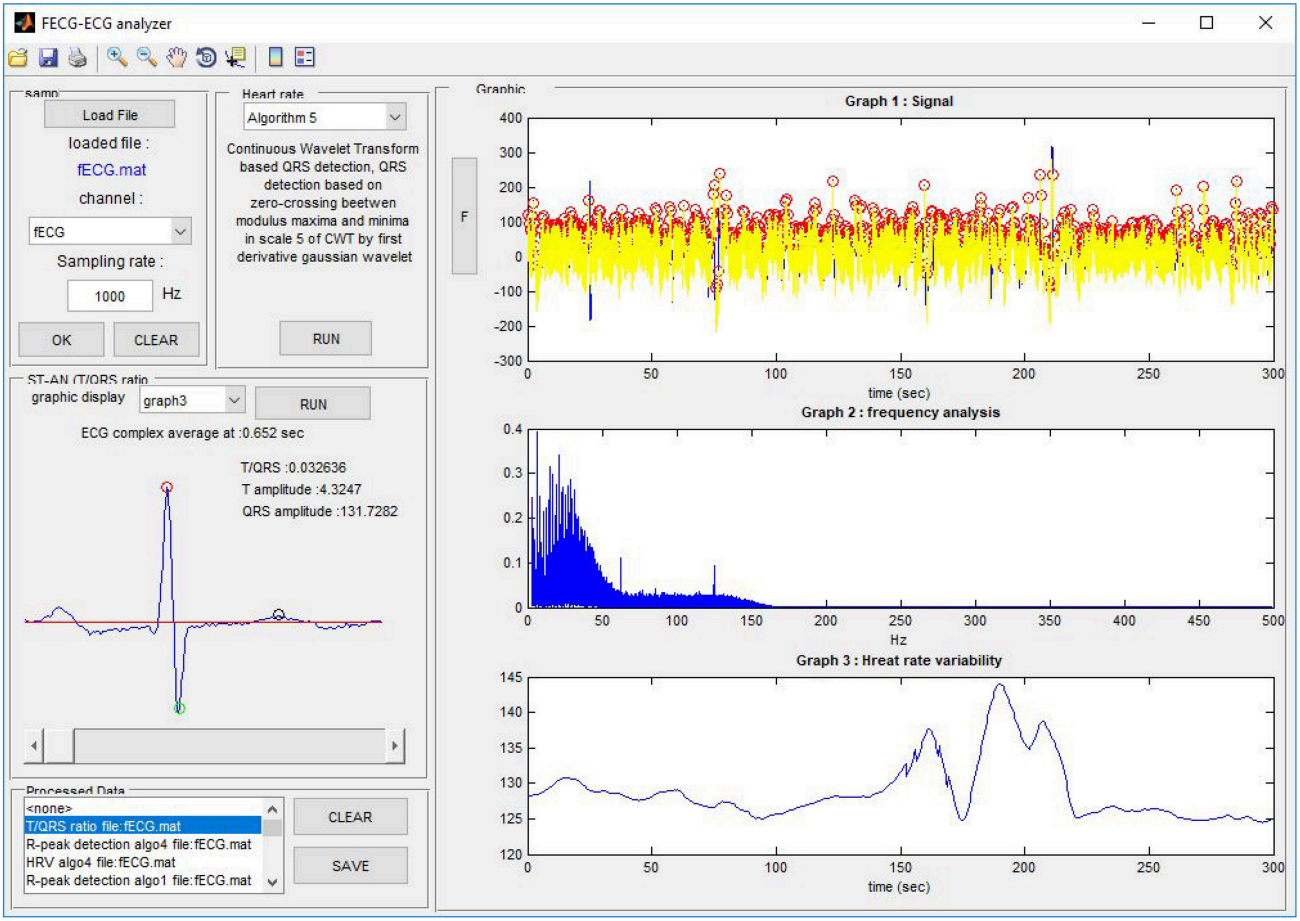
The Graphical User Interface (GUI) of our FECG-ECG Analyzer.

In addition to fHR determination and its variability (HRV), we focused on the morphological analysis of the estimated fECG signals extracted from the abdominal records, with primary emphasis on ST segment Analysis (STAN) and T/QRS determination. These capabilities have the potential to improve the diagnosis of fetal hypoxia (sensitivity, specificity) and decrease the number of unnecessary surgical terminations of pregnancy (cesarean sections). Besides, as currently there are no gold standards available for STAN, we are unfortunately unable to perform the quantitative verification and evaluation of the efficacy of our results. Consequently, our initial tests in this article include only the results for the T/QRS determination without deeper statistical analysis. The ST segment is considered to be the most variable part of the fECG signal. The changes in its duration and morphology may indicate pathological states. In particular, the elevation of the ST segment and the T-wave amplitude increase occur when the cardiovascular adaptation to hypoxia is no longer sufficient. STAN automatically detects and alerts changes that are related to the risk of fetal hypoxia (Rosen et al., 1992). In most cases, the method is used in combination with Cardiotocography (CTG) during labor and the interpretations follow the FIGO guidelines and recommendations (Ayres-De-Campos et al., 2015). It is based on calculating the ratio between T-waves and QRS complexes (T/QRS). The analysis of T/QRS in this paper was carried out in the fECG diagnostic tool that we have developed in our laboratory. Figure 24 shows the Graphical User Interface (GUI) of the application with some examples of the fECG signal analysis results. In our system, the STAN involved three different steps:

1. Localization of the R and S waves in the fECG signals followed by QRS complex detection and amplitude determination;
2. Detection of the R-peaks in the entire fECG signals by using the Continuous Wavelet Transform (CWT) based method;
3. Detection of the T-waves and their amplitude calculations. It is important to emphasize that due to the very low T-wave amplitudes, the mean of 30 Heartbeats (Cardiac Cycles) was used to calculate the T/QRS ratios.

Figure 24 shows the Graphical User Interface (GUI) of our application. It includes the part to load the File (data channel) to be analyzed as well as the sampling frequency. The user is able to load any file in the ^*^.edf or ^*^.mat formats. The panel Heart rate detection allows the user the R-peak detection and HRV calculation on previously loaded fECG data. The user can select between 6 available algorithms based on, for example, the Non-adaptive and Adaptive threshold for heartbeat detection, Discrete and Continuous WT, Neural network, and so on. There is a special panel to visualize the T/QRS analysis since it is a more advanced method for ECG feature analysis. To run a T/QRS ratio analysis (or STAN) the user must select one of the graphs to be analyzed. The details for samples from different recordings used for the experiments carried out in this paper are shown in Figure **26**.

**Figure 25:**
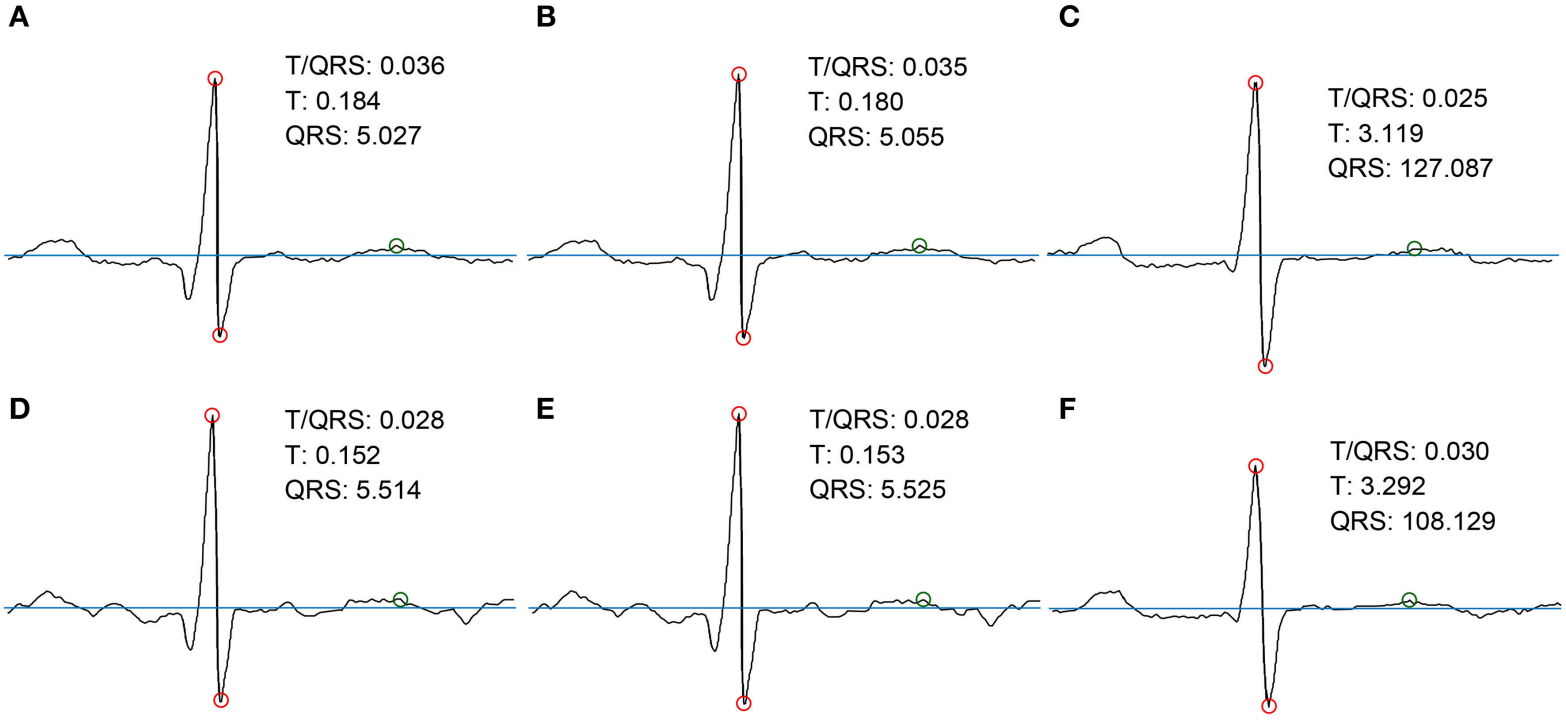
Examples of the morphological analysis of estimated and the reference fECG signals. **(A,D)** estimated signals using PCA method; **(B,E)** estimated signals using ICA method; **(C,F)** reference signals recorded by means of FSE.

The graphs in Figure 25 include 3 types of graphs for 2 recordings (real01 and real09): the fECG estimated by the ICA and PCA methods and the reference signal sensed by FSE. All of the graphs include details needed for STAN: the amplitudes of the T wave and QRS complex and the corresponding T/QRS ratio. The graphs prove that the morphological analysis is achievable in the case of high quality input data. Although the amplitudes of detected T waves and QRS complexes are lower than in the case of the signal from FSE (since the fECG signal is decreased while spreading toward the maternal abdomen) the T/QRS ratios remained unchanged. That means that improving the quality of fECG extraction techniques would allow a unique chance to provide STAN non-invasively in the future.

## 6. Discussion

**Figure 26:**
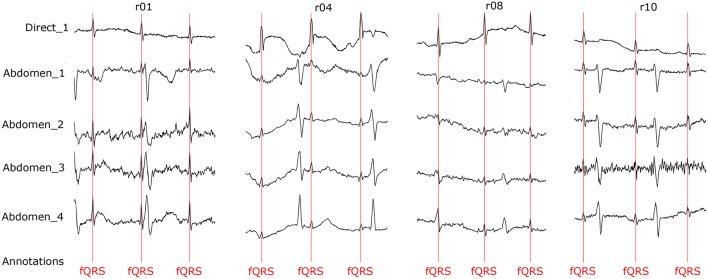
Quality assessment of the recordings used for our comparisons.

The results presented in section 6 prove that the extracted fECG signals fom aECG signals could serve as a valuable source of information and that using advanced signal processing methods, such as ICA and PCA, could enable the utility of this type of monitoring in clinical practice as an alternative to the conventional Doppler-based technique. Our statistical analysis results revealed that these non-adaptive methods did not perform very well for some of the recordings (real04, real06, real07, real10, and real11). The results of some unsatisfactory examples (recordings real04 and real10) are depicted in Figure 26. A possible explanation for such inferior results is that the magnitude of the fetal component in the abdominal signals is very low in comparison with the maternal one, especially in the case of the abdominal signal denoted as Abdomen_4, shown in the bottom of the figure. Here we are of the opinion that the gestation age was too low for accurate fHR determination. On the contrary, the results for recordings real01 and real08 were satisfactory. Note that the ratio between maternal and fetal components was significantly lower than those in other recordings. An interesting fact that can be observed in recording real10 is that the polarity of the maternal component is the inverse of the polarity in the rest of the recordings. This may be due to a different electrode placement, which could in turn negatively influence the performance of the tested algorithms. Nevertheless, the quality of the estimated signals is high enough to follow the trend of the fHR waveform, which is a key factor for accessing the fetal condition according to FIGO guidelines (Ayres-De-Campos et al., 2015).

We also need to emphasize that for reliable Morphological Analysis (for example, STAN), the quality of the analyzed fECG signal is of paramount importance and must be adequately high. For this reason STAN is currently performed exclusively on the fECG signals acquired invasively. Such recordings have higher Signal-to-Noise Ratios and therefore the amplitude and shape of the essential components of the fECG signals (T-waves and QRS complexes) are not distorted by contaminating signals. Considering these facts, the only records that could be used for NI-STAN in our work were real01, real02, real05, real08, and real09.

There are two main options for achieving sufficient signal quality of fECGs that is sufficient for NI-STAN: (1) by improving the quality of the input recordings (as discussed in chapter 6.4), or (2) utilizing more robust techniques, such as the aforementioned hybrid methods leveraging the combined capabilities and advantages of non-adaptive and adaptive methods. For NI-STAN, the key to success is the standardization (optimization) of the electrode placements that has to accommodate varying circumstances, especially gestation age and fetal position in the uterus.

This article is focused on two popular non-adaptive signal processing methods, even though the adaptive techniques have proven to be effective tools for fECG signal extraction from aECG signals. The adaptive methods are theoretically more suitable but have their drawbacks, mainly the need for the additional thoracic electrodes and leads (which restrict the pregnant woman's movements) and thus limit the utility of such methods in clinical practice. Furthermore, the efficiency of the adaptive methods is closely dependent on the adaptive filter's settings. The optimal filter settings vary with gestational age, fetal position in the uterus, etc. (Kahankova et al., 2017c). Adaptive methods have already been tested by our research group (Martinek and Zidek, 2012; Martinek et al., 2016a, 2017a,b; Fajkus et al., 2017) and others (Kam and Cohen, 1999; Reaz and Wei, 2004; Al-Zaben and Al-Smadi, 2006; Poularikas and Zayed, 2006; Assaleh, 2007; Czabanski et al., 2008; Shadaydeh et al., 2008; Jia et al., 2010; Swarnalatha and Prasad, 2010; Talha et al., 2010; Amin et al., 2011; Niknazar et al., 2013; Wei et al., 2013; Martinek et al., 2016c; Kahankova et al., 2017d, 2018). We need to emphasize that to achieve Morphological Analysis of fECG signals, adaptive methods appear to be more suitable. However, for clinical applications, non-adaptive methods offer the advantage of solely using abdominal electrodes without the need for thoracic electrodes and leads. In addition, there is a trend in SMART technologies in medicine to use a multisource system, where the electrodes are embedded within a garment (one electrode grid/strip for the abdominal area). Such a system seems to be very promising for the future of fetal monitoring. In this kind of system, the ICA and PCA methods applied and tested in our research could prove to be the most suitable for implementation. Nevertheless, our aim here was not only to determine fHR values but also to achieve Morphological Analysis of the fECG waveforms. Our experimental results proved that using advanced signal processing techniques could enable further Morphological Analysis (for example, STAN), see section 6.5.

In our future research, we will focus on leveraging the utility of the combined adaptive and non-adaptive (hybrid) methods. With this approach we would benefit from the advantages offered by both methods. The hybrid methods would be more accurate for detailed Morphological Analysis while making use of the abdominal recordings alone. Our approach would be based on extracting the reference fECG signals from the aECG signals using the ICA and PCA methods. However, we needed to test adaptive and non-adaptive methods separately at first before reaching that goal.

The evaluation of our results was based on comparing the estimated signals with the reference fECG signals recorded by means of the FSE. Five of the recordings used in our experimentation are currently available from the Abdominal and Direct Fetal Electrocardiogram Database Jezewski et al. (2012). The other 7 are new and are not publicly available yet. It is important to emphasize that for the acquisition of data recorded in the entire Database, the type of measurements, sampling frequency, and other details changed. For this reason, we thought it would be beneficial to our readers to describe the details of these measurements. Professor Jezewski, a member of our team and our co-author, intends to make the rest of the Database acquired at ITAM in Poland available to the public by means of PhysioNet.

We obtained the reference fECG signals from raw data in the Database and subsequently processed and annotated them in cooperation with a team of experts including both engineers and clinicians. The R-wave peak detection was semi-automatic. First, we used the Continuous Wavelength Transform to detect the R-waves. Subsequently, we went through all the recordings and visually inspected the detection of the RR intervals to eliminate any automatic errors, see Figure 26. This visual quality-assurance step was important for the initial phase of our research not only to ensure the accurate detection of any errors (due to fetal movements in the uterus, incorrect electrode placement, and others); but to find solutions for eliminating them (see Supplementary Material for all figures and tables).

## 7. Conclusion and future work

In this paper, we compared the effectiveness of two popular non-adaptive signal separation techniques, the ICA and PCA methods, to extract fECG signals from aECG signals noninvasively. The performance of these conventionally used methods was improved by an adaptive algorithm, compensating amplitude difference and time shift between the estimated components. Currently, the non-adaptive methods are successfully used for fHR determination. However, there are still great research opportunities and scope for improvements, especially in terms of further Morphological Analysis. We provided the performance evaluation results on real data based on comparing the extracted signals with the reference fECG signals recorded by means of the FSE. The novelty in our work is centered around obtaining the reference signals from raw data that were subsequently processed and annotated by a team of experts. Thus, in contrast to the other results reported in the field (see Kahankova et al., 2017c; Martinek et al., 2017a), we were not limited by the lack of gold standards (fQRS annotations in the abdominal signals) as we could use our own reference data for further investigation. We have developed a software tool for Morphological Analysis (for example STAN) of the fECG waveforms. Since we have both reference and estimated fECG signals available, we are able to apply more sophisticated analysis by means of our software tool. For this reason, we could evaluate the robustness of the extraction algorithms for fHR determination and further Morphological Analysis.

The results demonstrated the effectiveness of the improved conventional methods, ICA and PCA, for fHR determination and further Morphological Analysis. The performance of the methods was influenced by several factors. The inferior performance of the methods may be attributable to the impact of the fetal position in the uterus or fetal and maternal movements. Moreover, the electrode placement varies during pregnancy and it is influenced by the Gestation Age (GA). The ICA and PCA methods have the potential to be utilized as early as the 20th week of pregnancy since the signals are too weak for the extraction before that period. As a fundamental limitation in the utility of the ICA and PCA methods for extracting fECG signals, the case of multiple pregnancy must be mentioned since these non-adaptive methods may have problems in extracting the components with same frequencies (fHRs of each fetus in the uterus). Similarly, problems could arise in pathological cases when the maternal and fetal heart rates increase/decrease by the same amount. Generally, the fetal beating frequency is about 2 times greater than the maternal rate. However, in case of fetal bradycardia or maternal tachycardia, these frequencies might have almost the same value.

At present, a great deal of attention is being paid to single-channel methods. It is obvious that for more non-invasive STAN, multichannel recordings are needed. A Single-channel signal is likely to be of low quality due to several factors such as changes in the fetal position and others. Single-channel methods are sufficient to determine the fHR, but for deeper Morphological Analysis they are rather inadequate.

We envision that a multichannel system is essential for the advanced analysis of non-invasively acquired fECG signals. In our future research, we aim to explore the combined utility of non-adaptive and adaptive signal processing methods in order to achieve better results. This approach could lead to the development of a new diagnostic method: non-invasive STAN (NI-STAN).

## Ethics statement

The study protocol was approved by the Ethical Committee of the Silesian Medical University, Katowice, Poland (NN-013-345/02). Subjects read the approved consent form and gave written informed consent to participate in the study.

## Author contributions

RM, RK, JJ, RJ, JM, MF, JN, PJ, and HN proposed the system idea, wrote, and edited the manuscript. RM, MF, and JN performed the experiments, developed, tested, and validated the software. HN, JJ, and PJ critically evaluated the scientific validity of the proposed system and the acquired vital data, wrote the manuscript, and performed its final edits.

### Conflict of interest statement

The authors declare that the research was conducted in the absence of any commercial or financial relationships that could be construed as a potential conflict of interest. The reviewer AF and handling Editor declared their shared affiliation.
