# Supplementary figures and images for: Comparative Effectiveness of ICA and PCA in Extraction of Fetal ECG From Abdominal Signals: Toward Non-invasive Fetal Monitoring
Source: Front Physiol. 2018 May 30;9:648. doi: 10.3389/fphys.2018.00648 (PMC5988877; doi:10.3389/fphys.2018.00648)

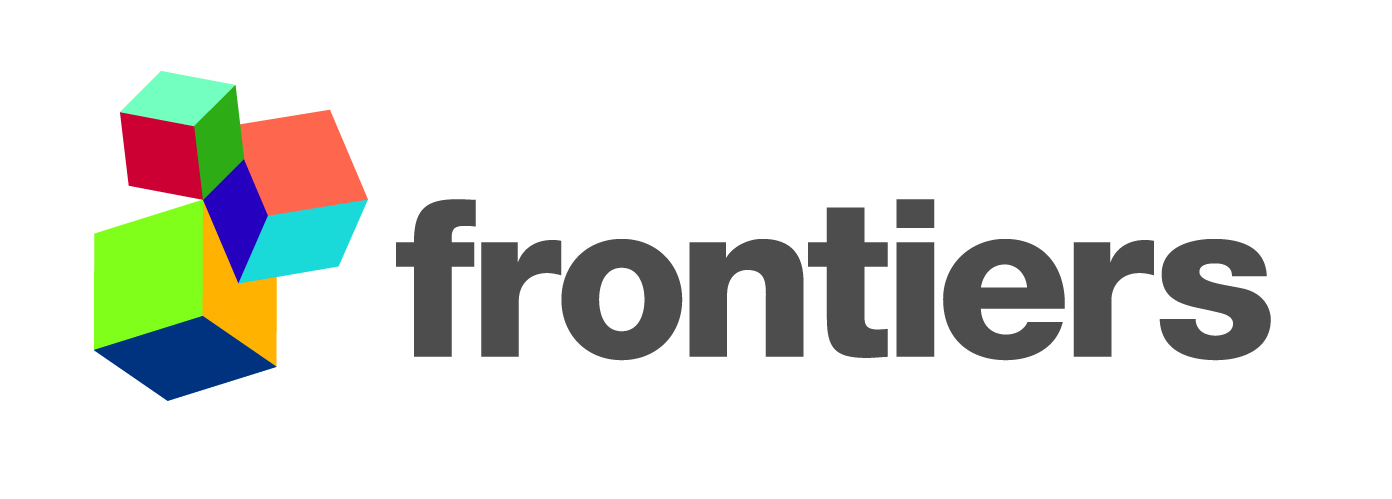

Supplement: Supplementary file 1 [file Data_Sheet_1.ZIP › logo1.jpg]

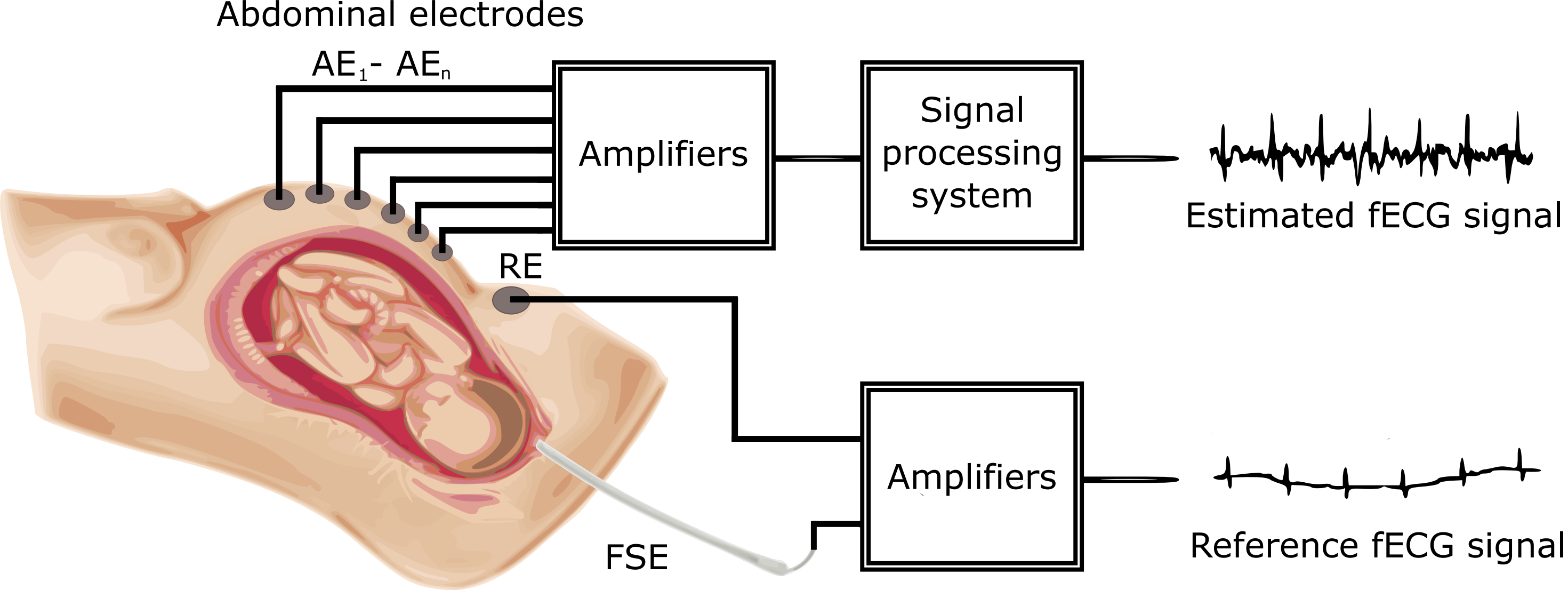

Supplement: Supplementary file 2 [file Data_Sheet_2.ZIP › fig/fig-01.pdf]

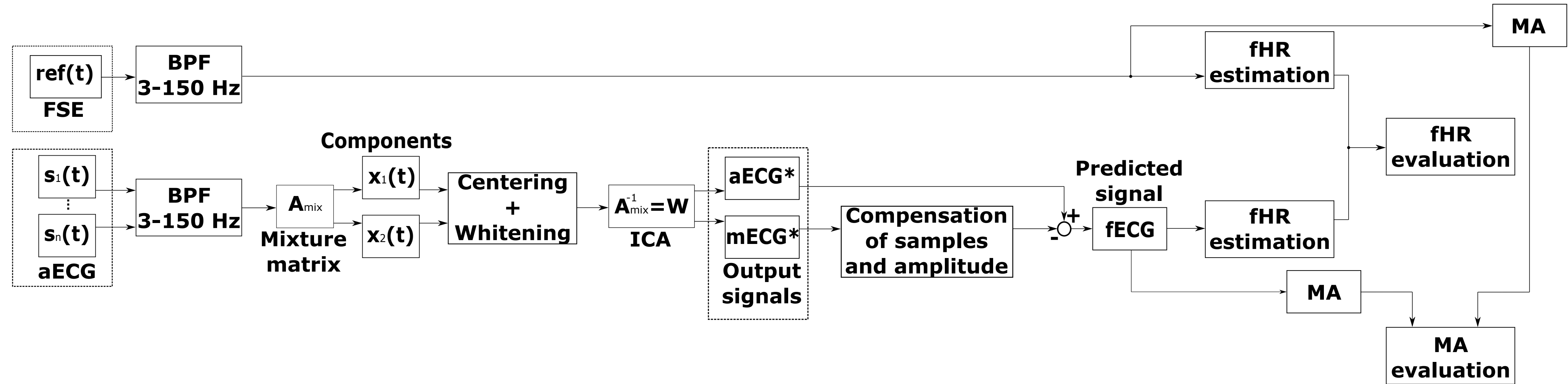

Supplement: Supplementary file 2 [file Data_Sheet_2.ZIP › fig/fig-02.pdf]

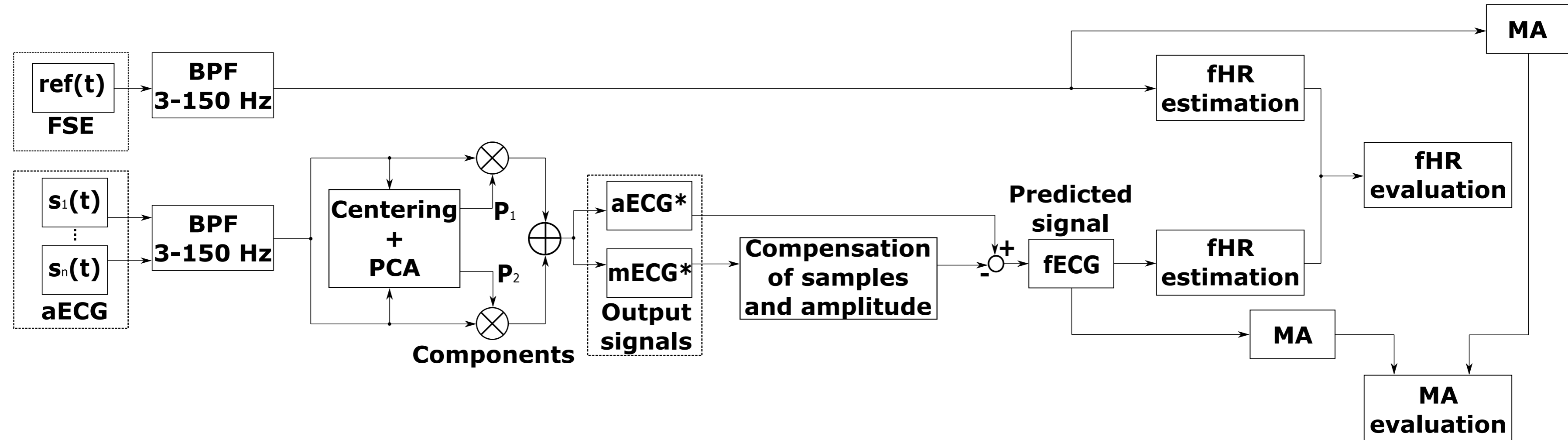

Supplement: Supplementary file 2 [file Data_Sheet_2.ZIP › fig/fig-04.pdf]

$$U = [u_1, u_2]$$

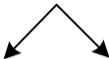

A

$x_2$

B

$S_2$

$S_1$

C

$x_1$

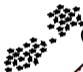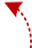

Supplement: Supplementary file 2 [file Data_Sheet_2.ZIP › fig/fig-05.pdf]

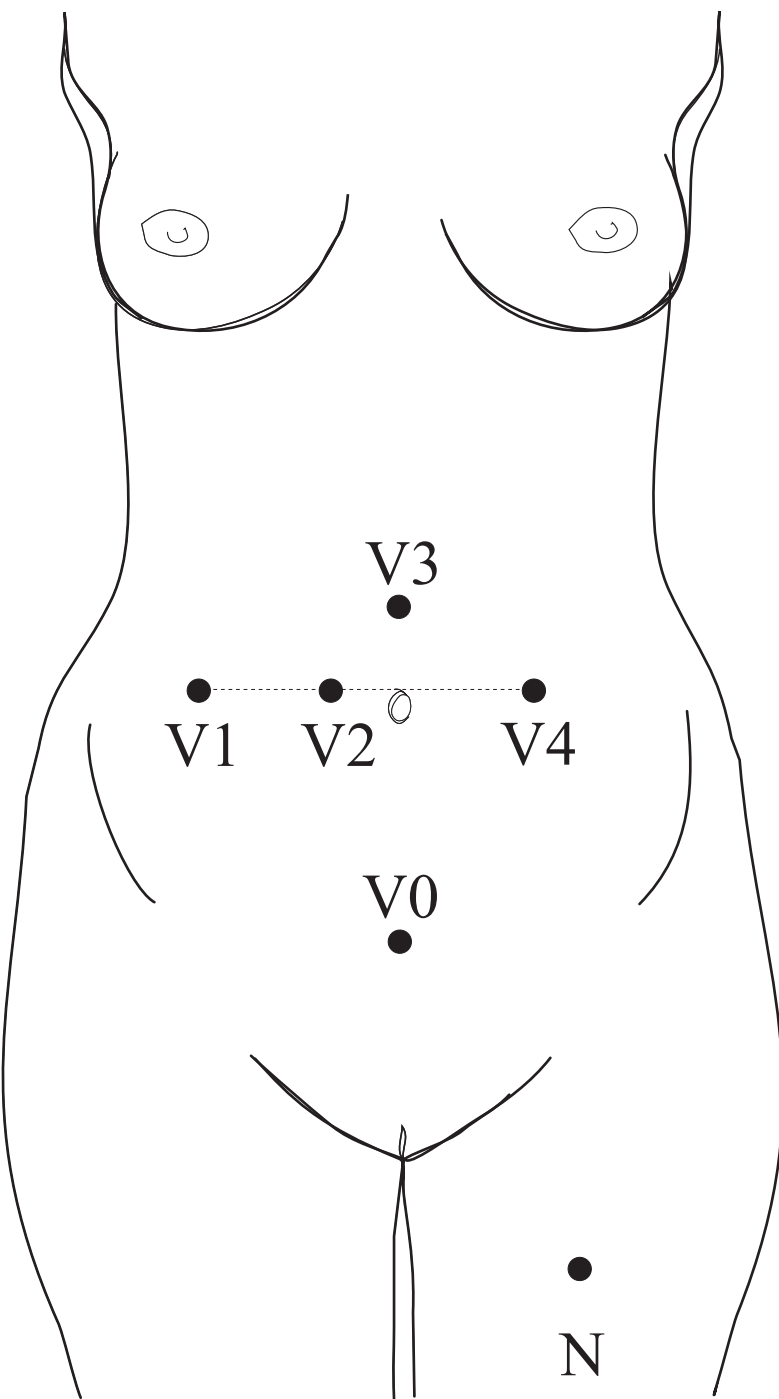

V0 - reference electrode  
N - active ground

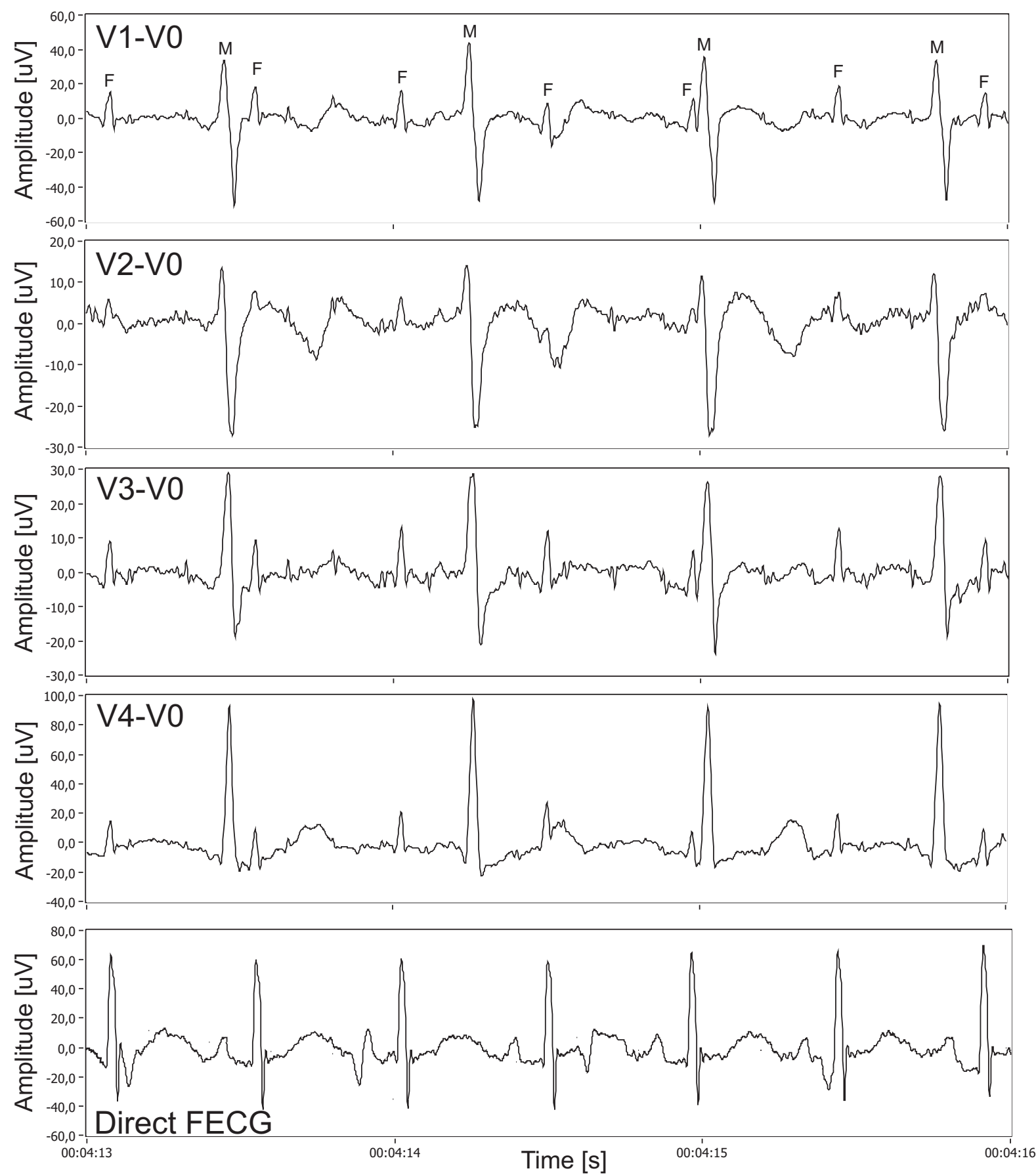

Supplement: Supplementary file 2 [file Data_Sheet_2.ZIP › fig/fig-06-eps-converted-to.pdf]

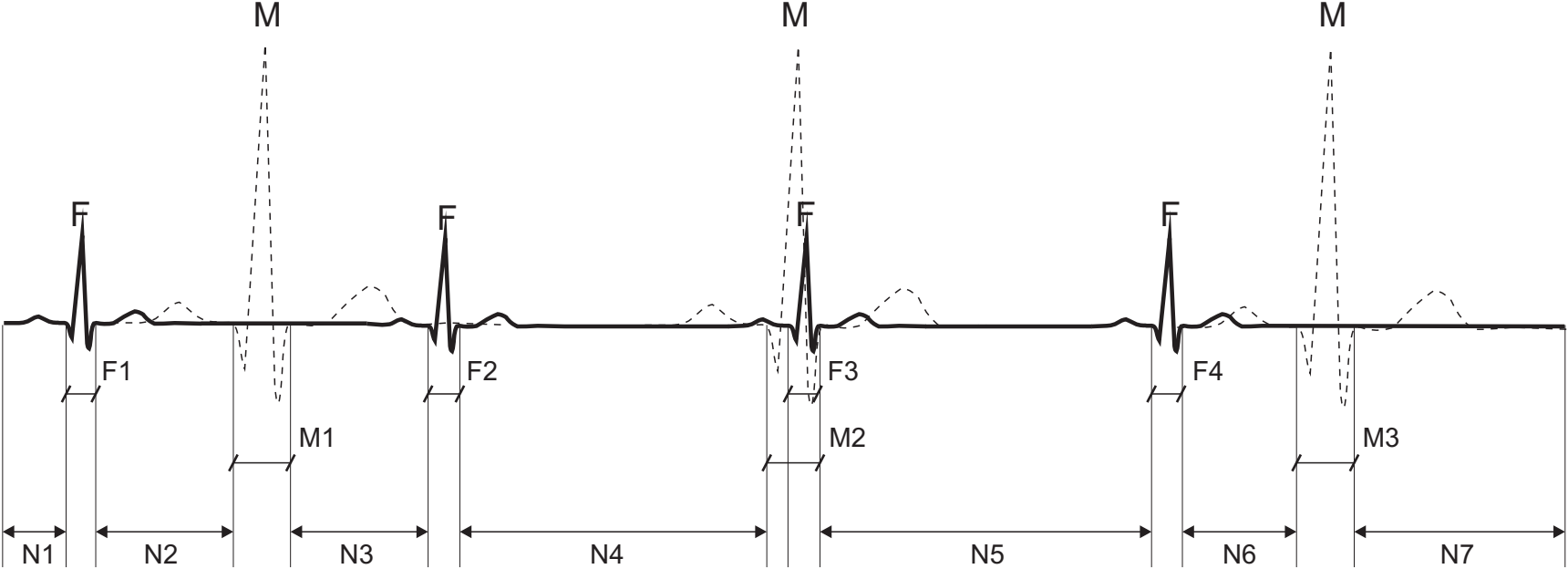

Supplement: Supplementary file 2 [file Data_Sheet_2.ZIP › fig/fig-07-eps-converted-to.pdf]

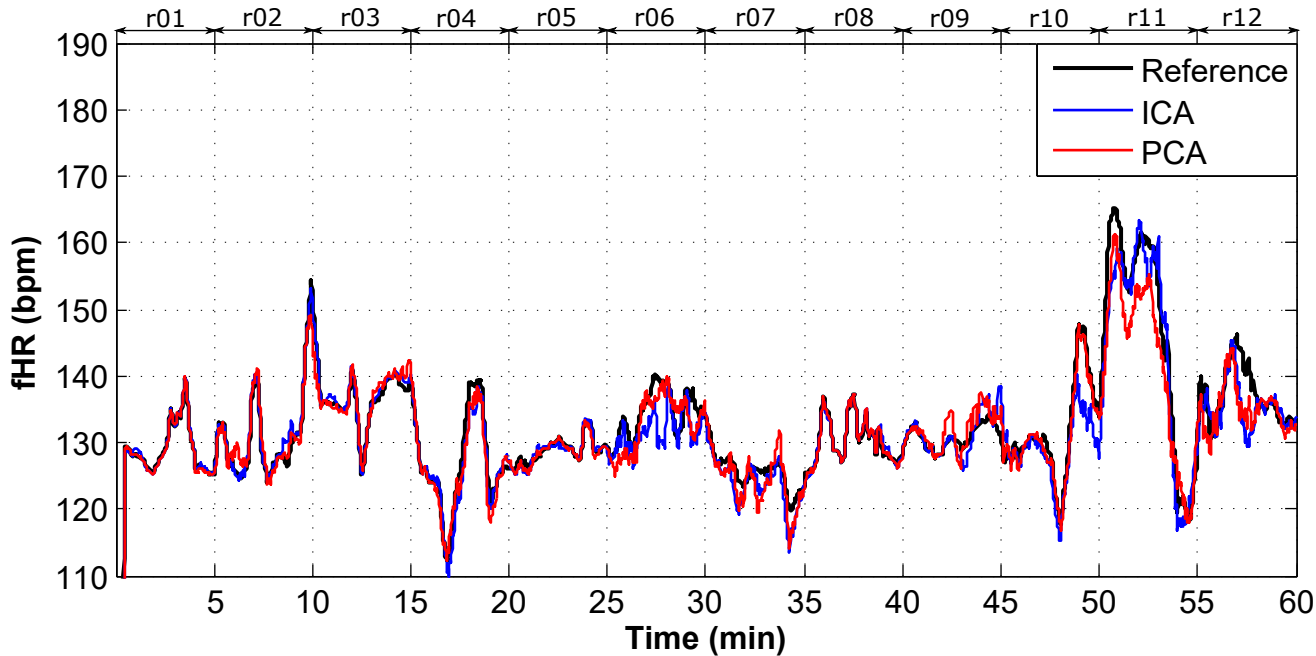

Supplement: Supplementary file 2 [file Data_Sheet_2.ZIP › fig/fig-08.pdf]

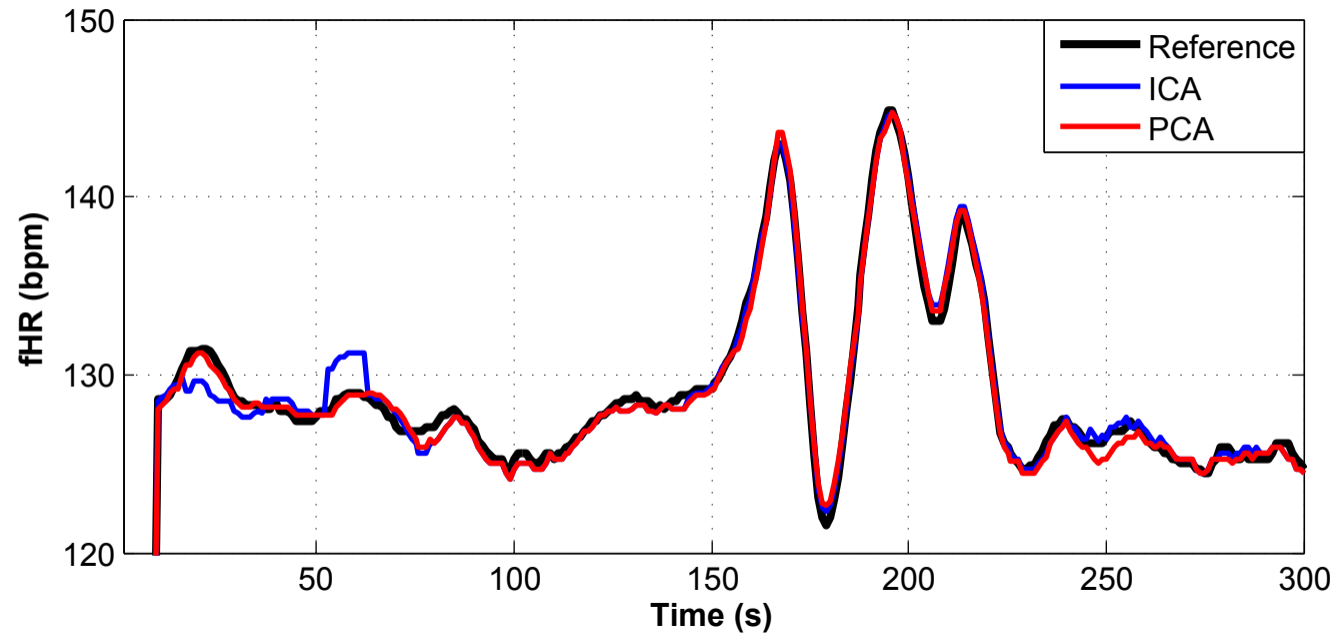

Supplement: Supplementary file 2 [file Data_Sheet_2.ZIP › fig/fig-09.pdf]

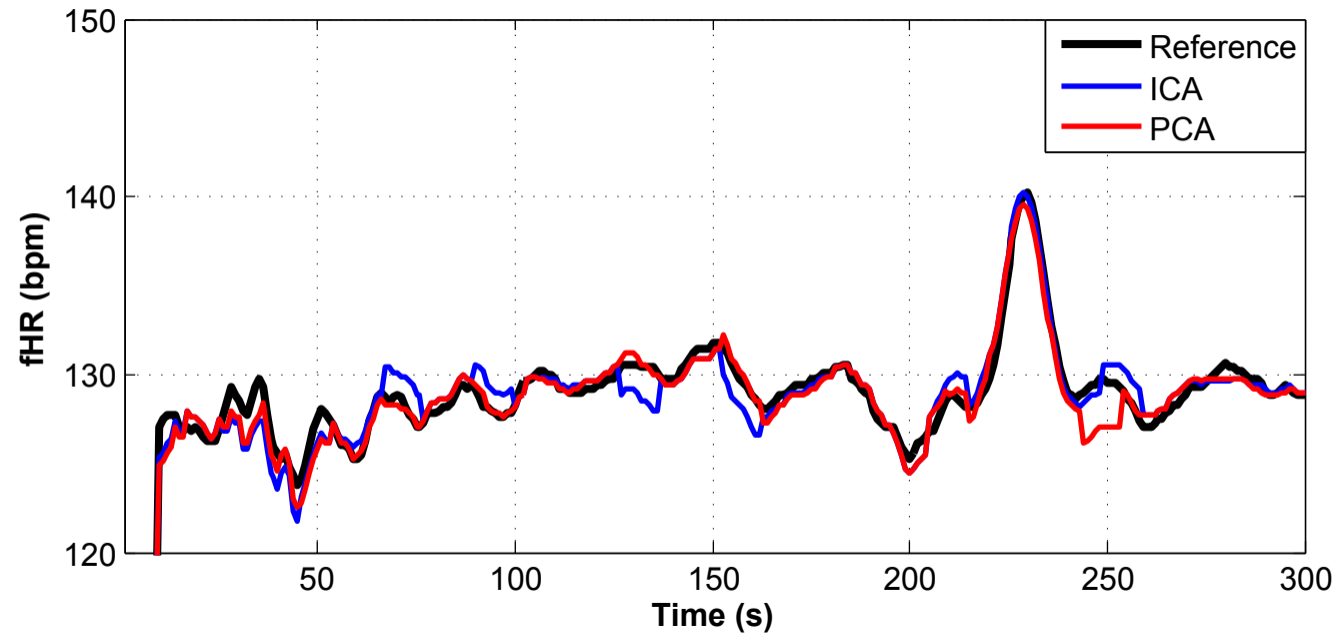

Supplement: Supplementary file 2 [file Data_Sheet_2.ZIP › fig/fig-10.pdf]

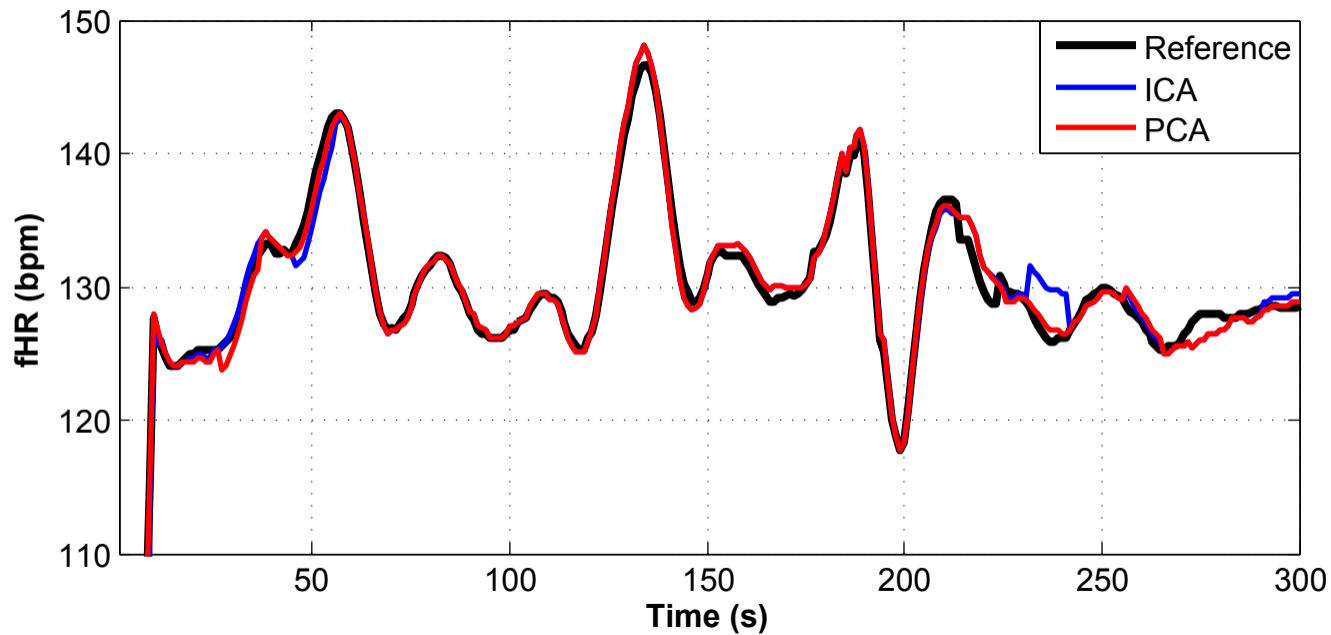

Supplement: Supplementary file 2 [file Data_Sheet_2.ZIP › fig/fig-11.pdf]

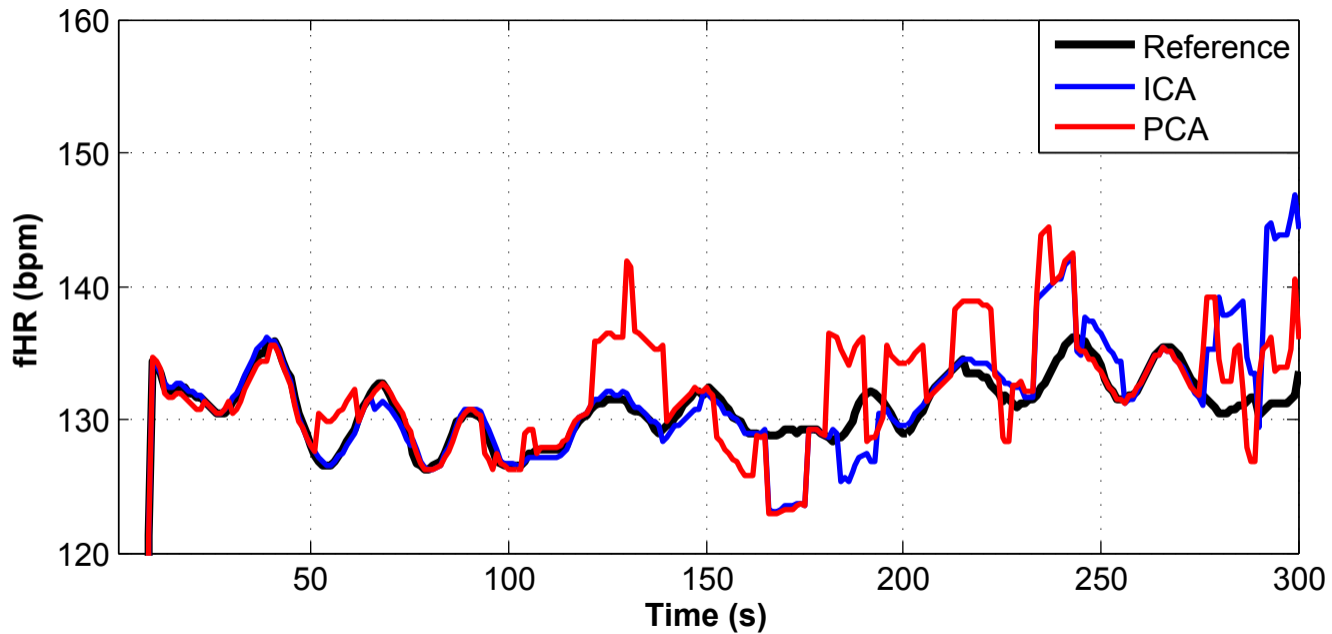

Supplement: Supplementary file 2 [file Data_Sheet_2.ZIP › fig/fig-12.pdf]

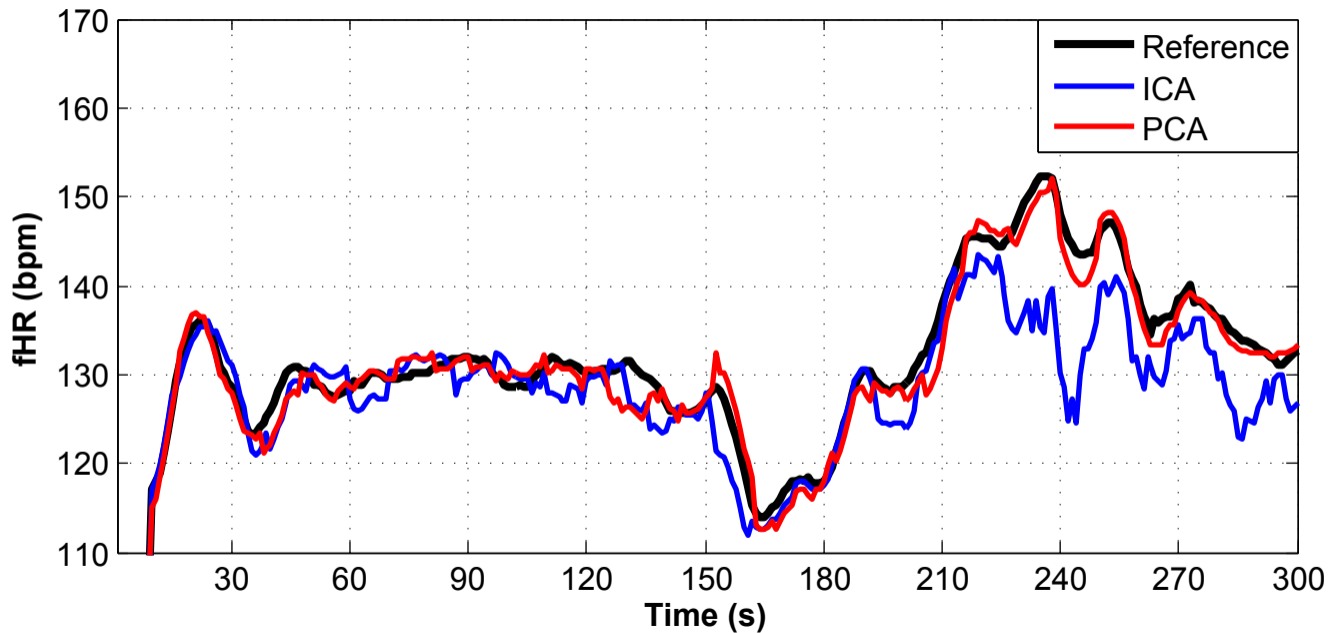

Supplement: Supplementary file 2 [file Data_Sheet_2.ZIP › fig/fig-13.pdf]

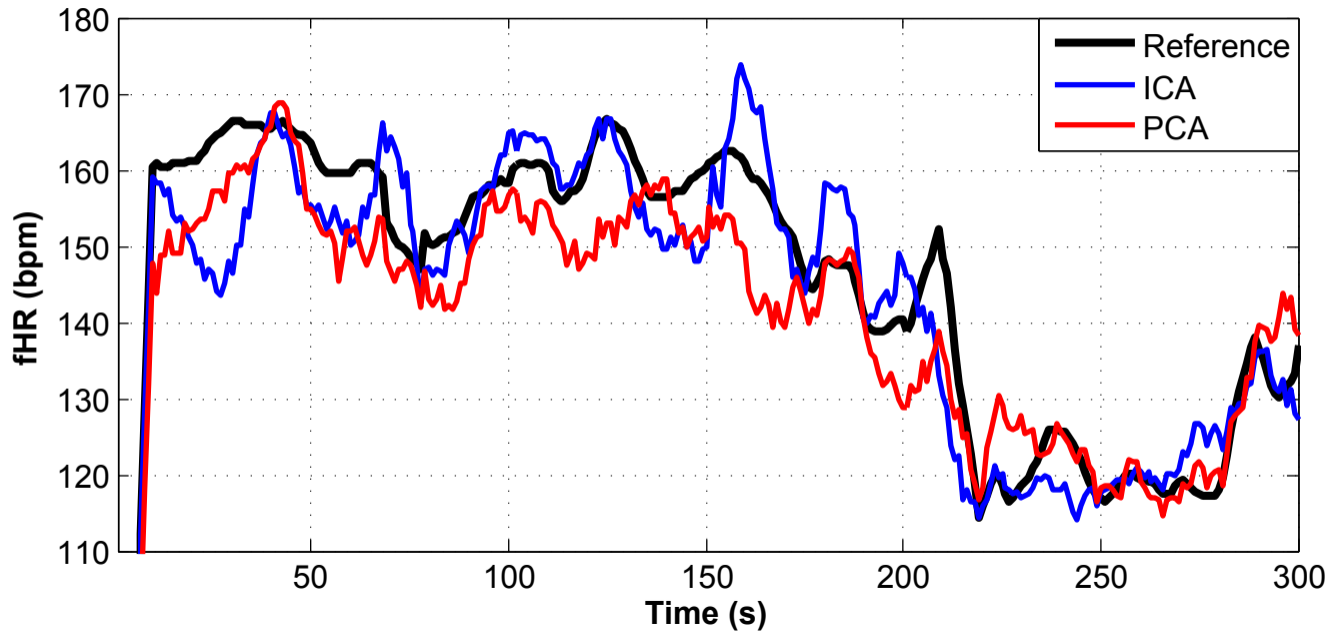

Supplement: Supplementary file 2 [file Data_Sheet_2.ZIP › fig/fig-14.pdf]

a)

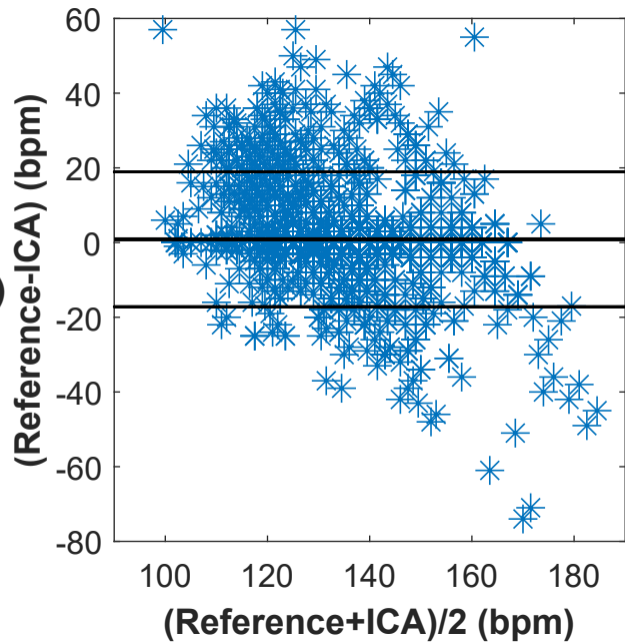

b)

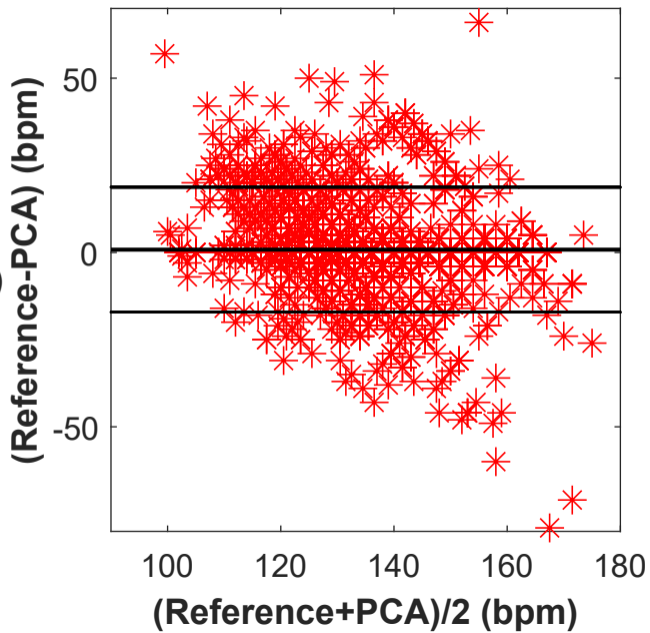

Supplement: Supplementary file 2 [file Data_Sheet_2.ZIP › fig/fig-15.pdf]

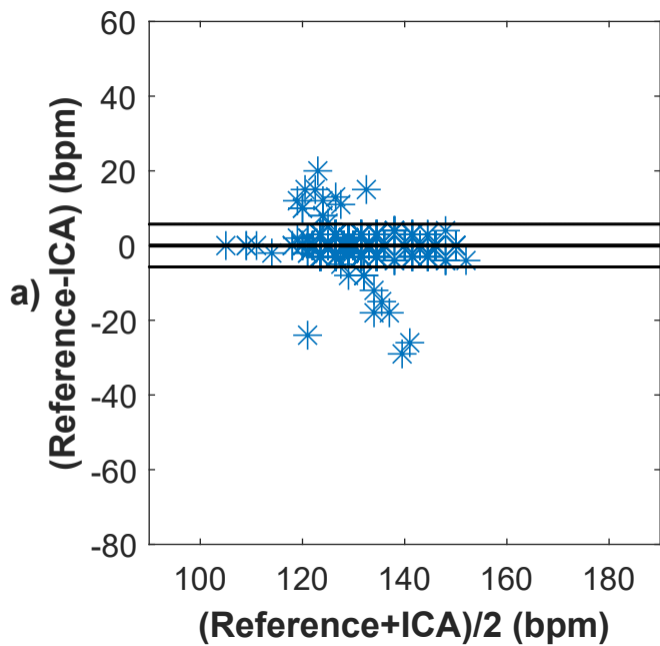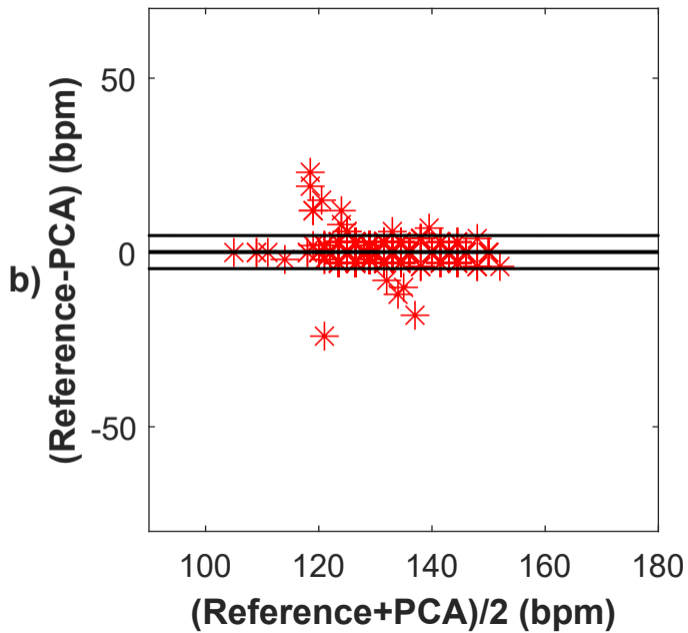

Supplement: Supplementary file 2 [file Data_Sheet_2.ZIP › fig/fig-16.pdf]

a)

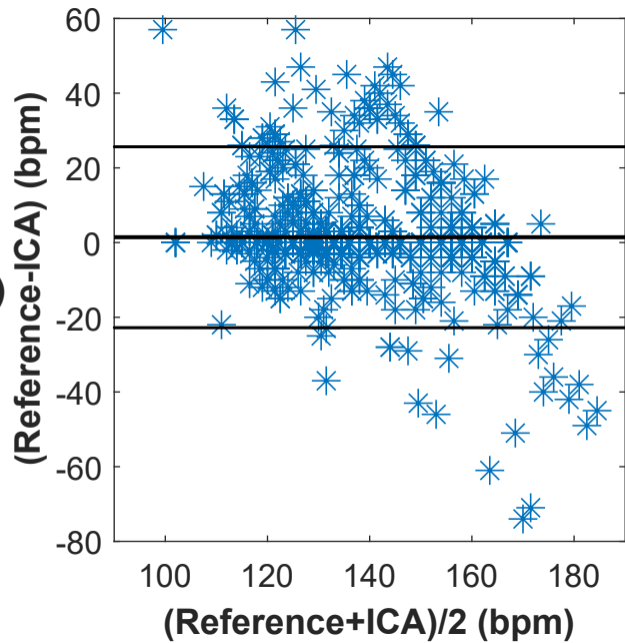

b)

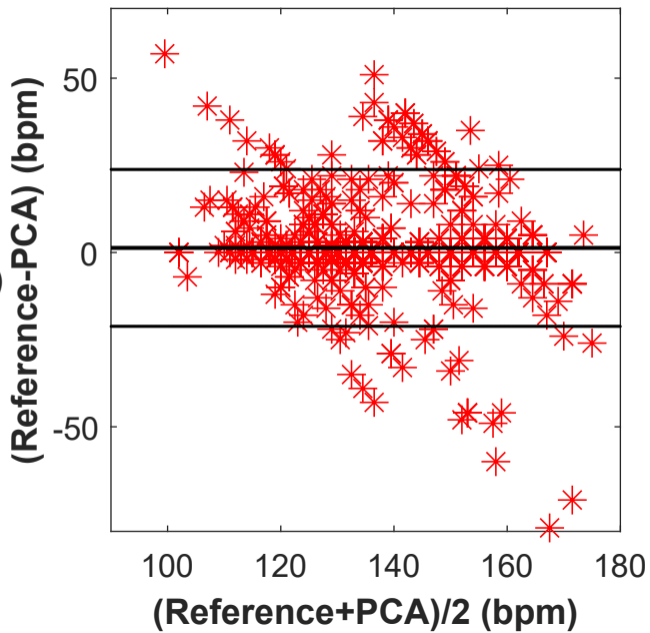

Supplement: Supplementary file 2 [file Data_Sheet_2.ZIP › fig/fig-17.pdf]

a)

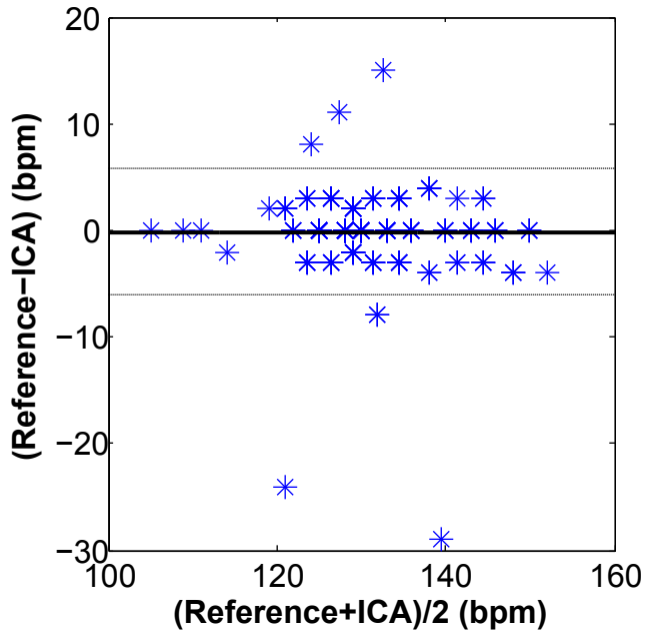

b)

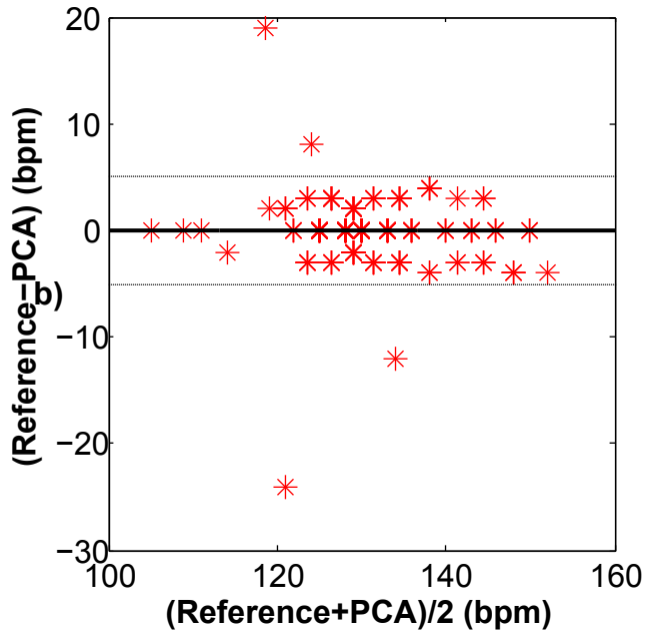

Supplement: Supplementary file 2 [file Data_Sheet_2.ZIP › fig/fig-18.pdf]

a)

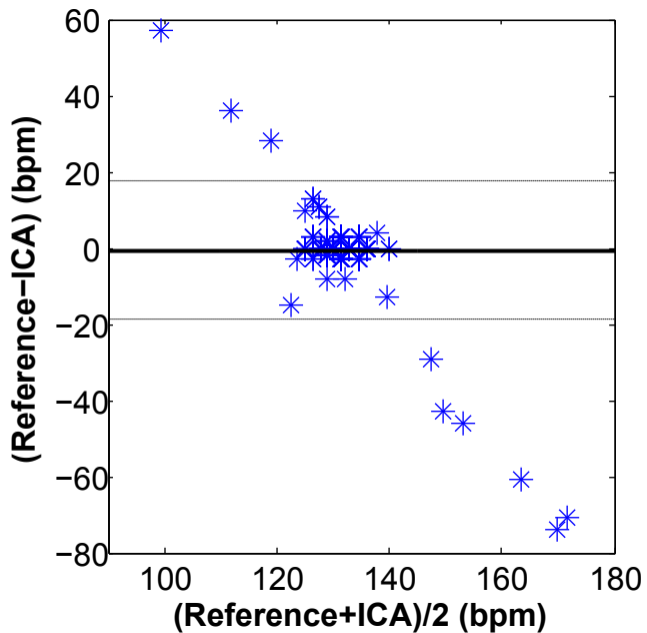

b)

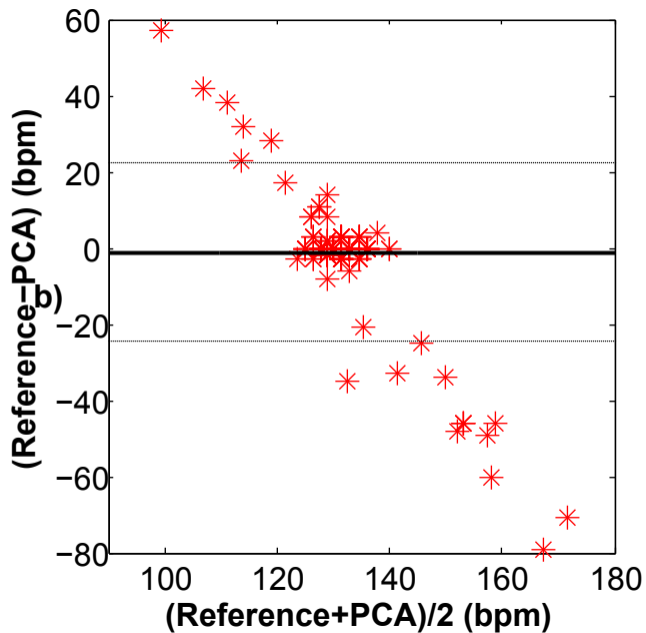

Supplement: Supplementary file 2 [file Data_Sheet_2.ZIP › fig/fig-19.pdf]

a)

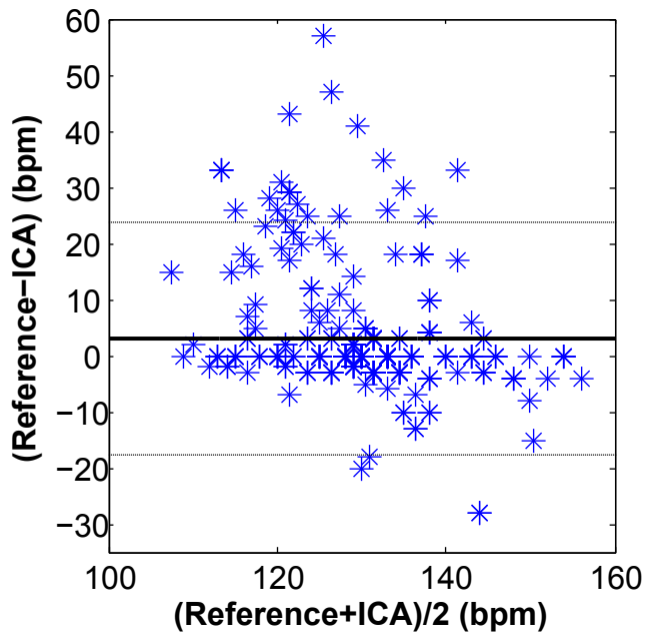

b)

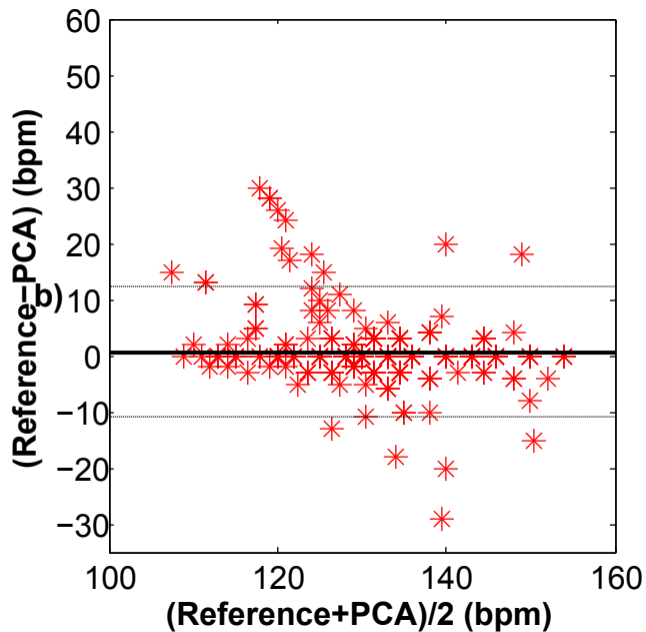

Supplement: Supplementary file 2 [file Data_Sheet_2.ZIP › fig/fig-20.pdf]

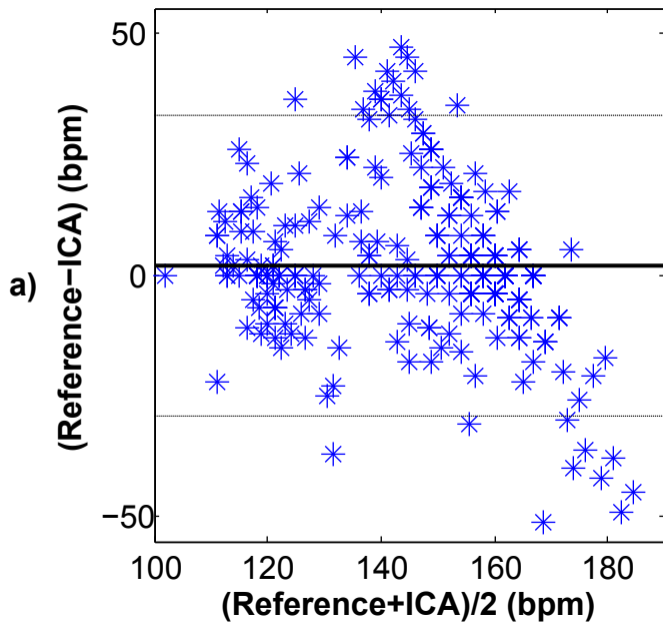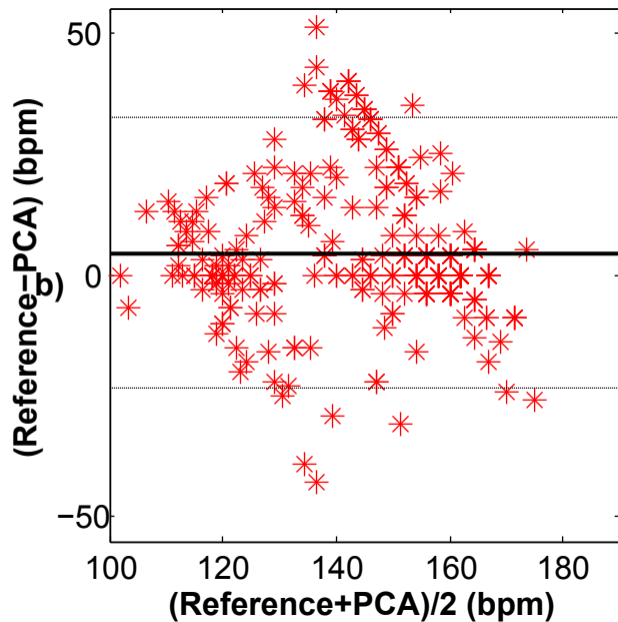

Supplement: Supplementary file 2 [file Data_Sheet_2.ZIP › fig/fig-21.pdf]

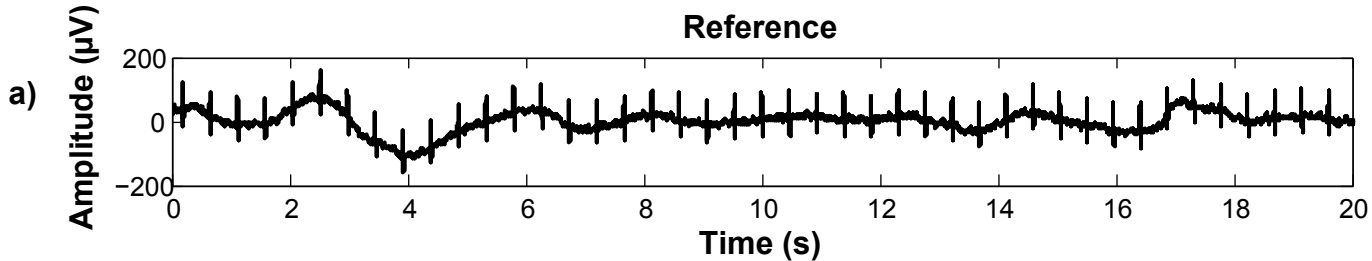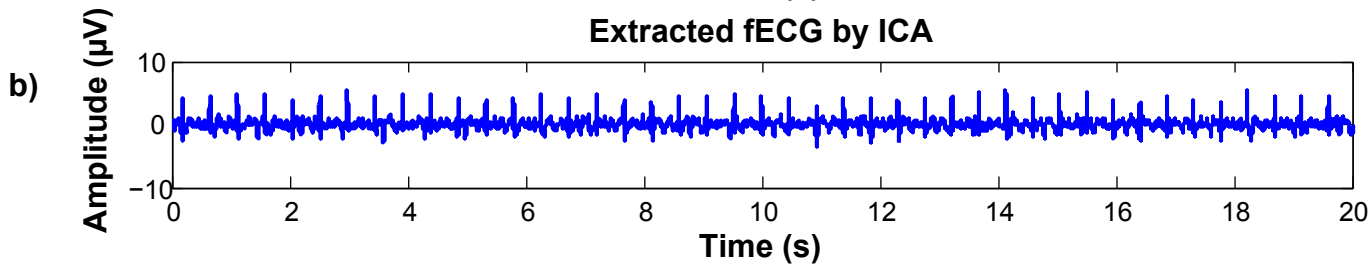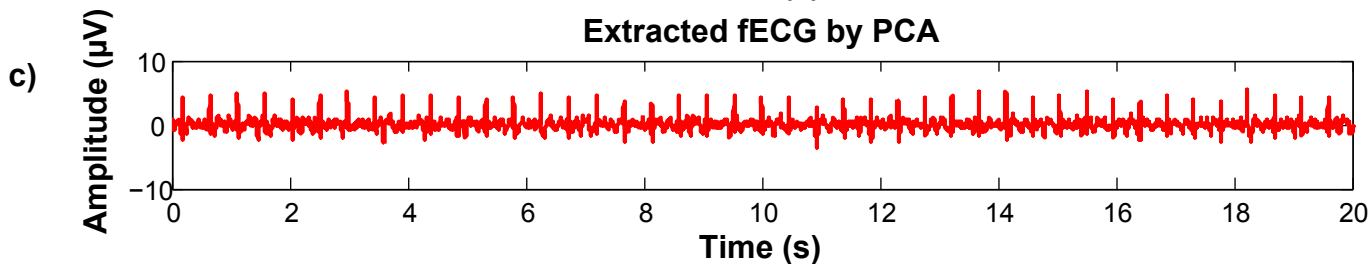

Supplement: Supplementary file 2 [file Data_Sheet_2.ZIP › fig/fig-22.pdf]

**a)**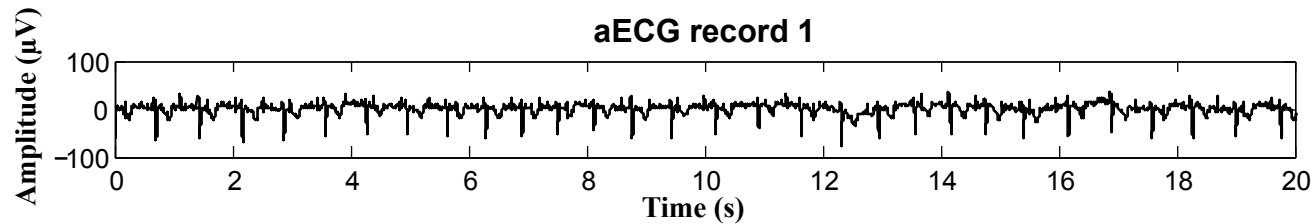**b)**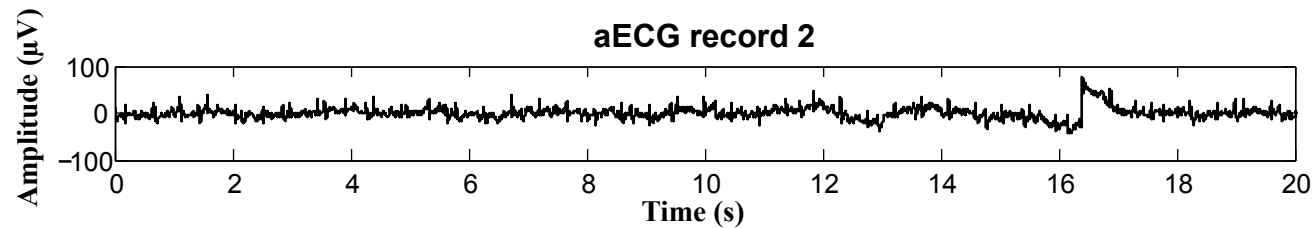**c)**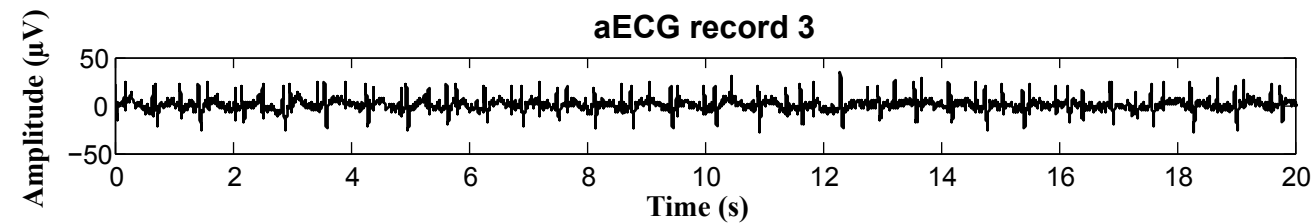**d)**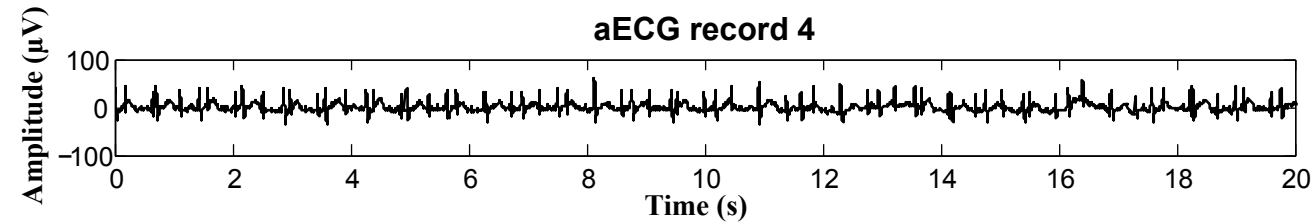

Supplement: Supplementary file 2 [file Data_Sheet_2.ZIP › fig/fig-23.pdf]

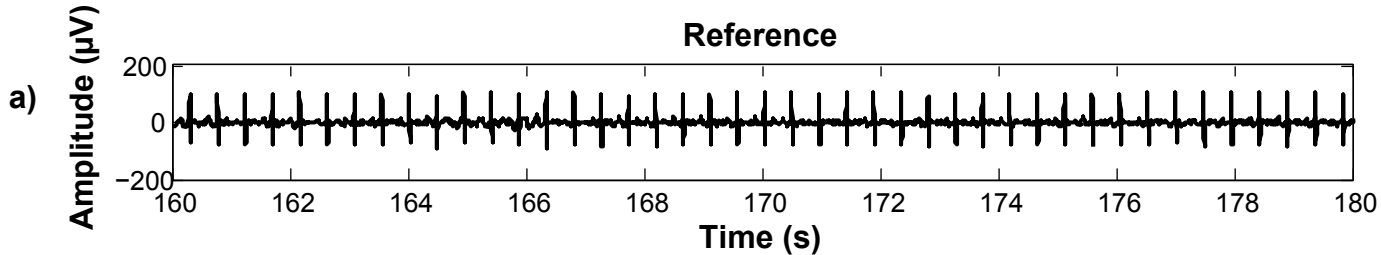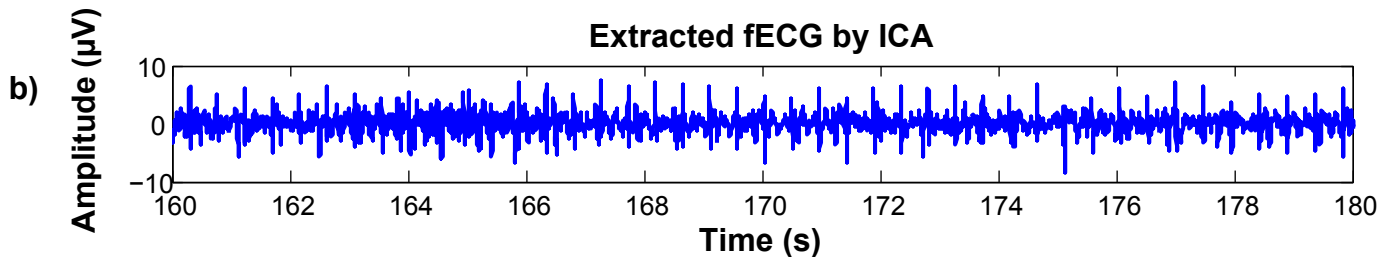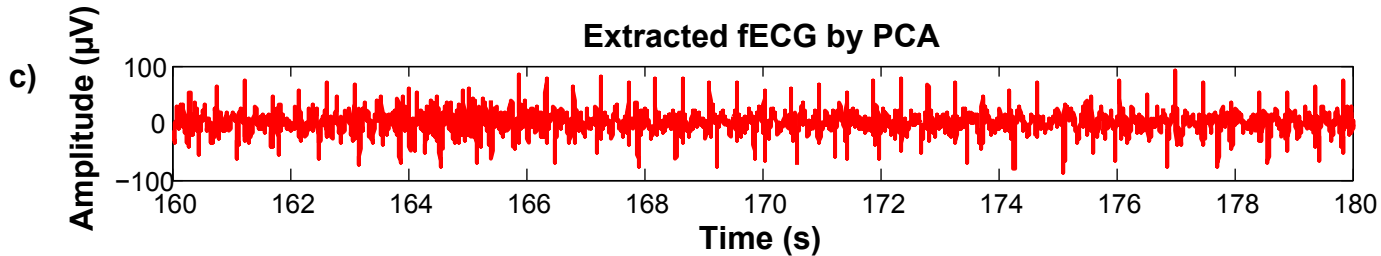

Supplement: Supplementary file 2 [file Data_Sheet_2.ZIP › fig/fig-24.pdf]

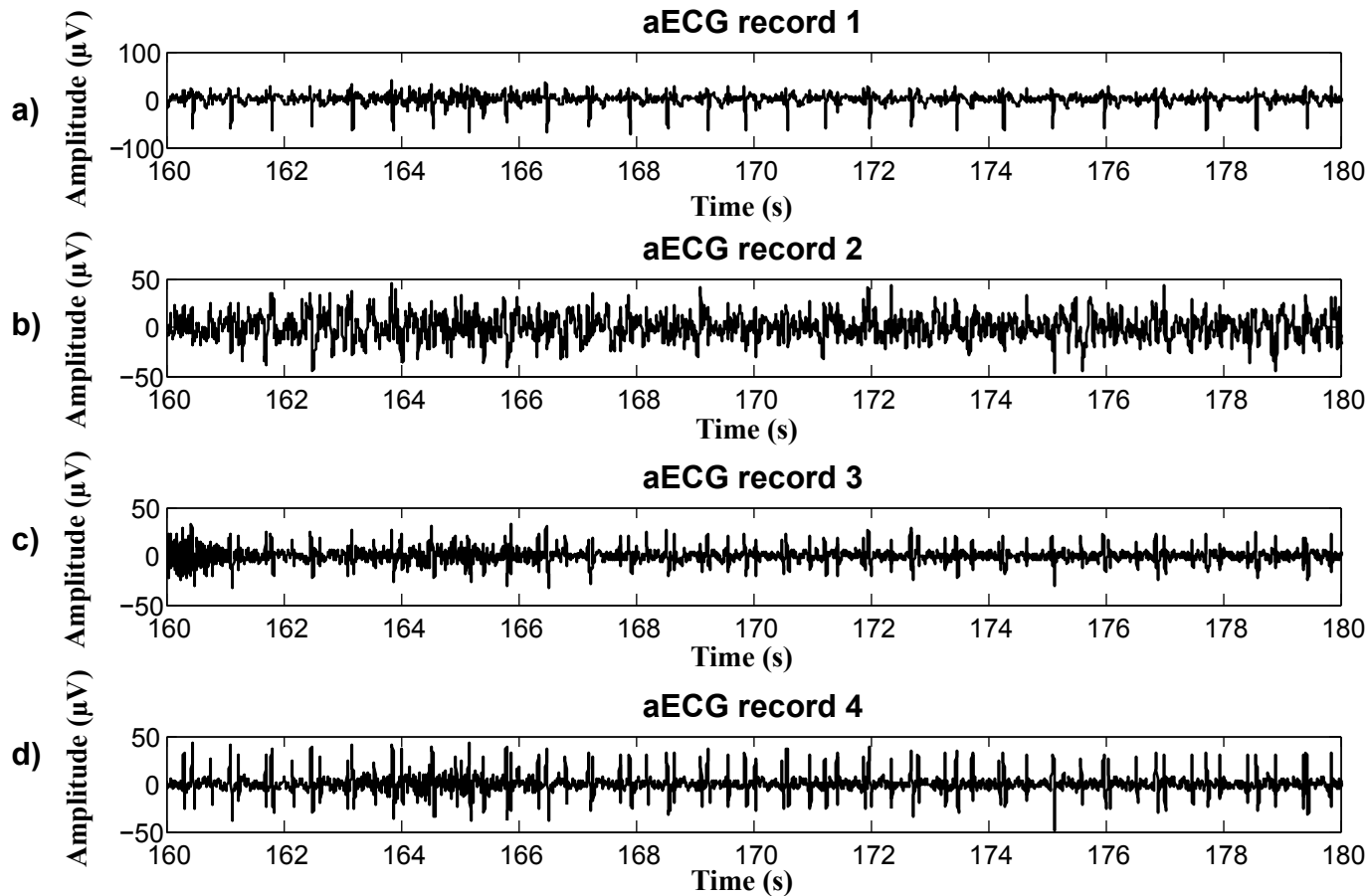

Supplement: Supplementary file 2 [file Data_Sheet_2.ZIP › fig/fig-25.pdf]

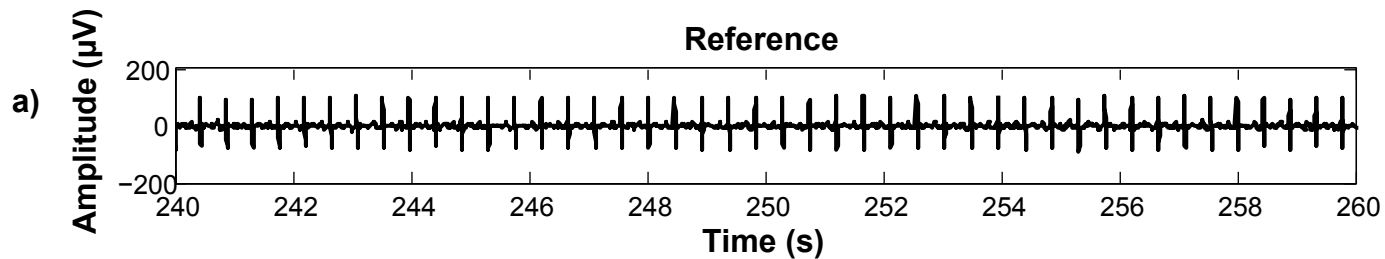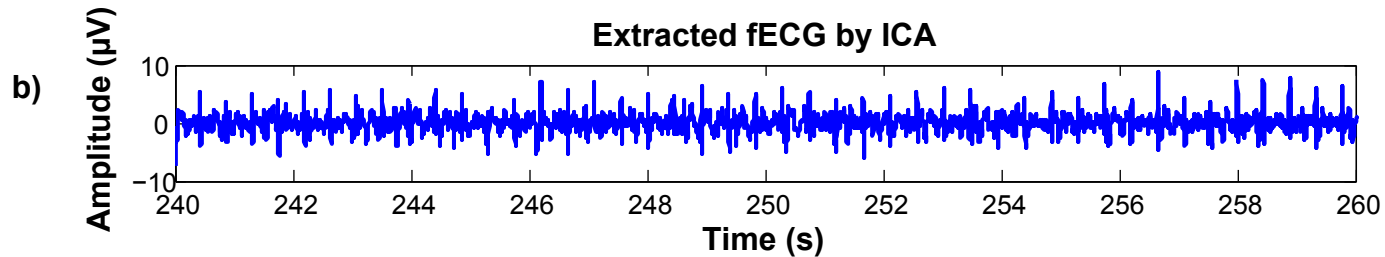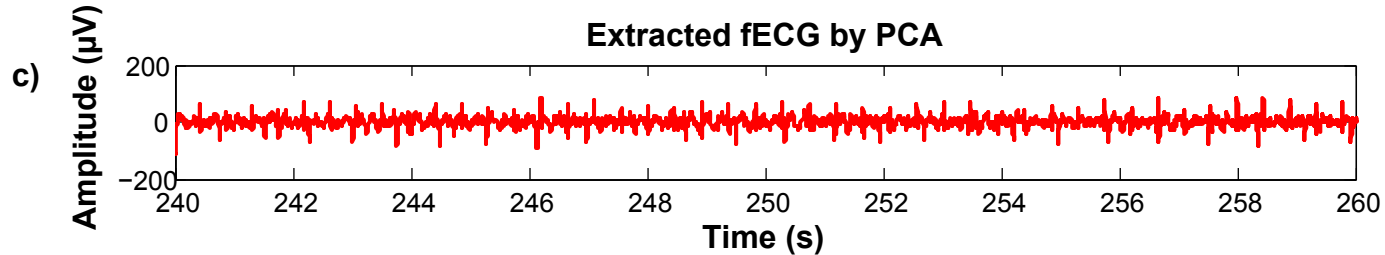

Supplement: Supplementary file 2 [file Data_Sheet_2.ZIP › fig/fig-26.pdf]

**a)**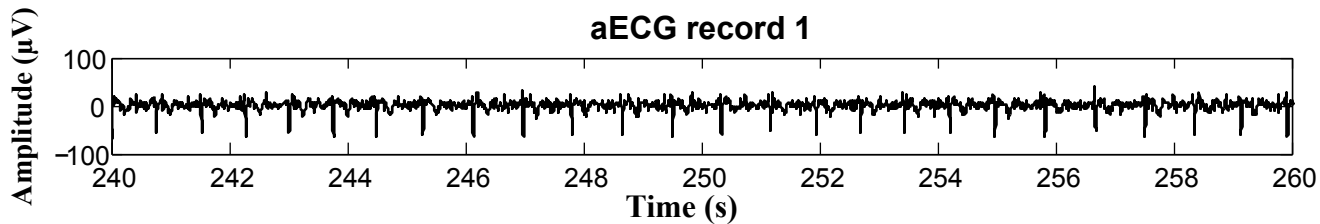**b)**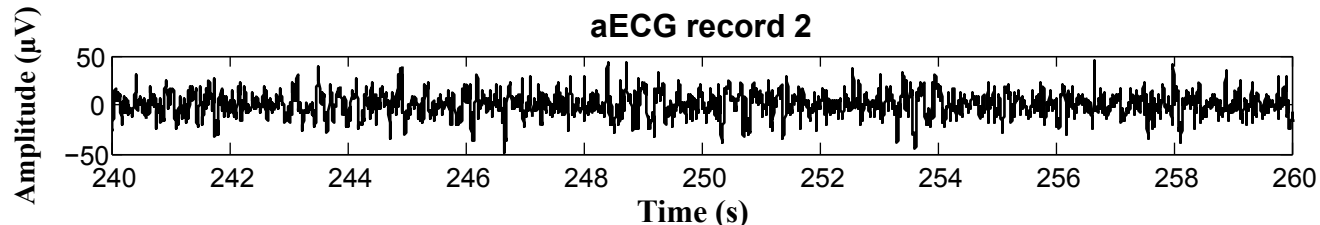**c)**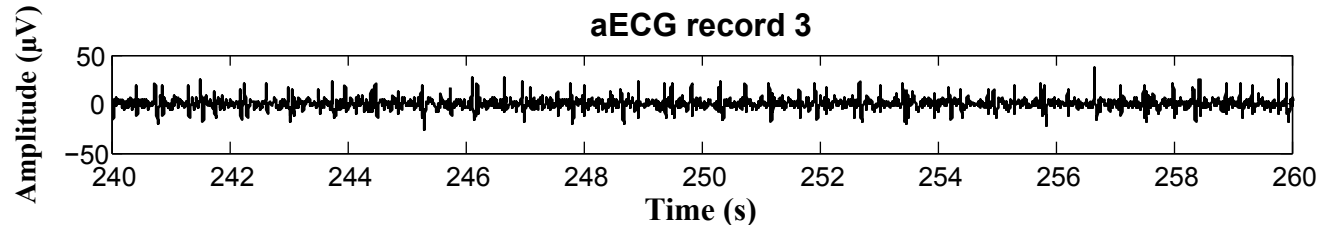**d)**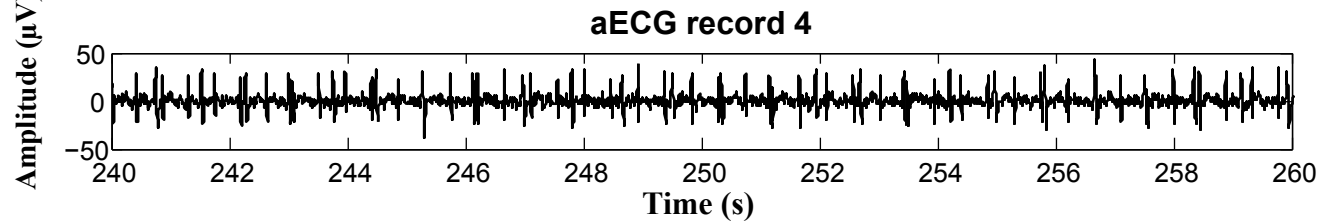

Supplement: Supplementary file 2 [file Data_Sheet_2.ZIP › fig/fig-27.pdf]

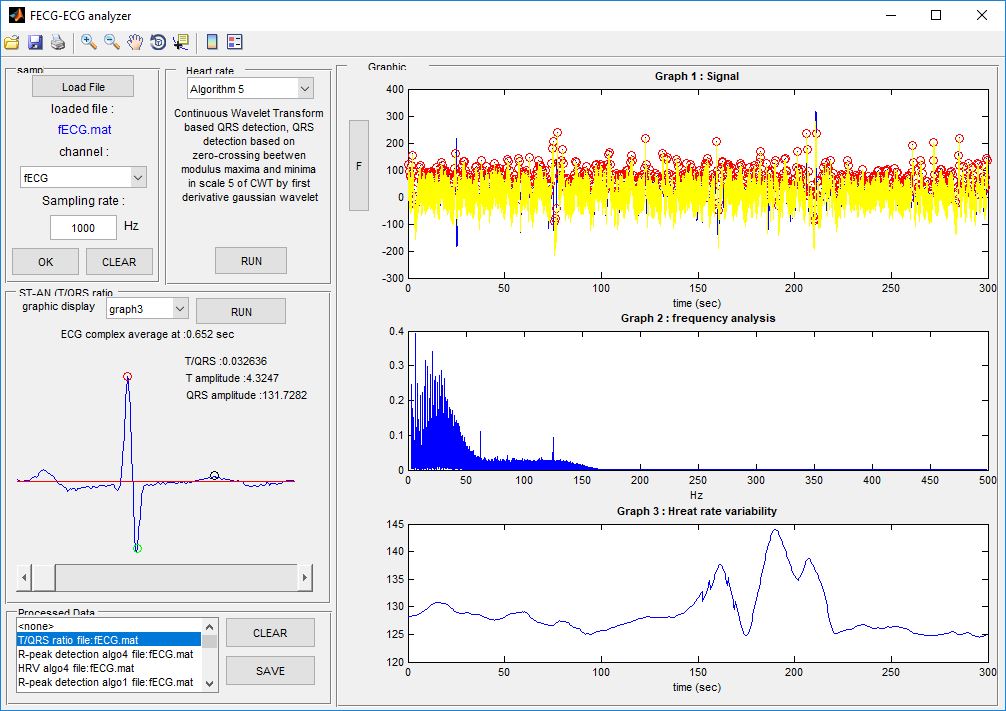

Supplement: Supplementary file 2 [file Data_Sheet_2.ZIP › fig/fig-28.JPG]

T/QRS: 0.036

T: 0.184

QRS: 5.027

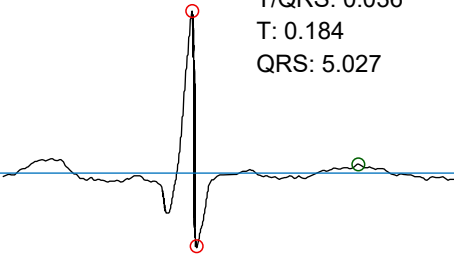

Supplement: Supplementary file 2 [file Data_Sheet_2.ZIP › fig/fig-29a.pdf]

T/QRS: 0.035

T: 0.180

QRS: 5.055

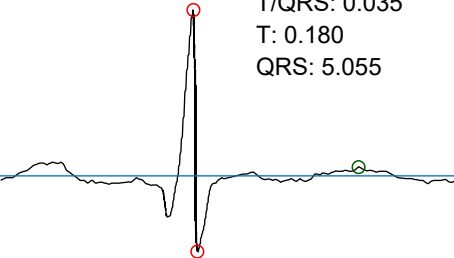

Supplement: Supplementary file 2 [file Data_Sheet_2.ZIP › fig/fig-29b.pdf]

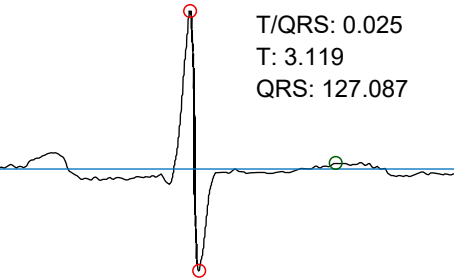

Supplement: Supplementary file 2 [file Data_Sheet_2.ZIP › fig/fig-29c.pdf]

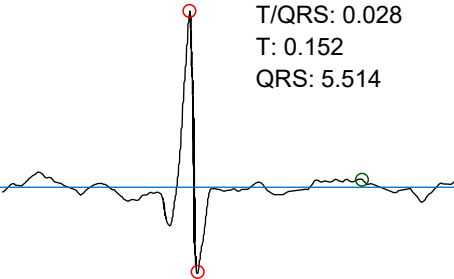

T/QRS: 0.028

T: 0.152

QRS: 5.514

Supplement: Supplementary file 2 [file Data_Sheet_2.ZIP › fig/fig-29d.pdf]

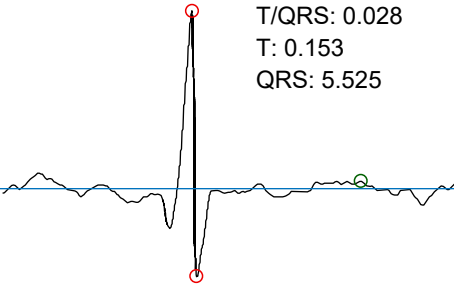

Supplement: Supplementary file 2 [file Data_Sheet_2.ZIP › fig/fig-29e.pdf]

T/QRS: 0.030

T: 3.292

QRS: 108.129

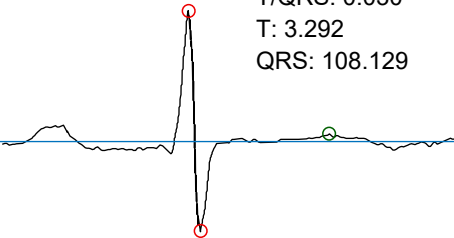

Supplement: Supplementary file 2 [file Data_Sheet_2.ZIP › fig/fig-29f.pdf]

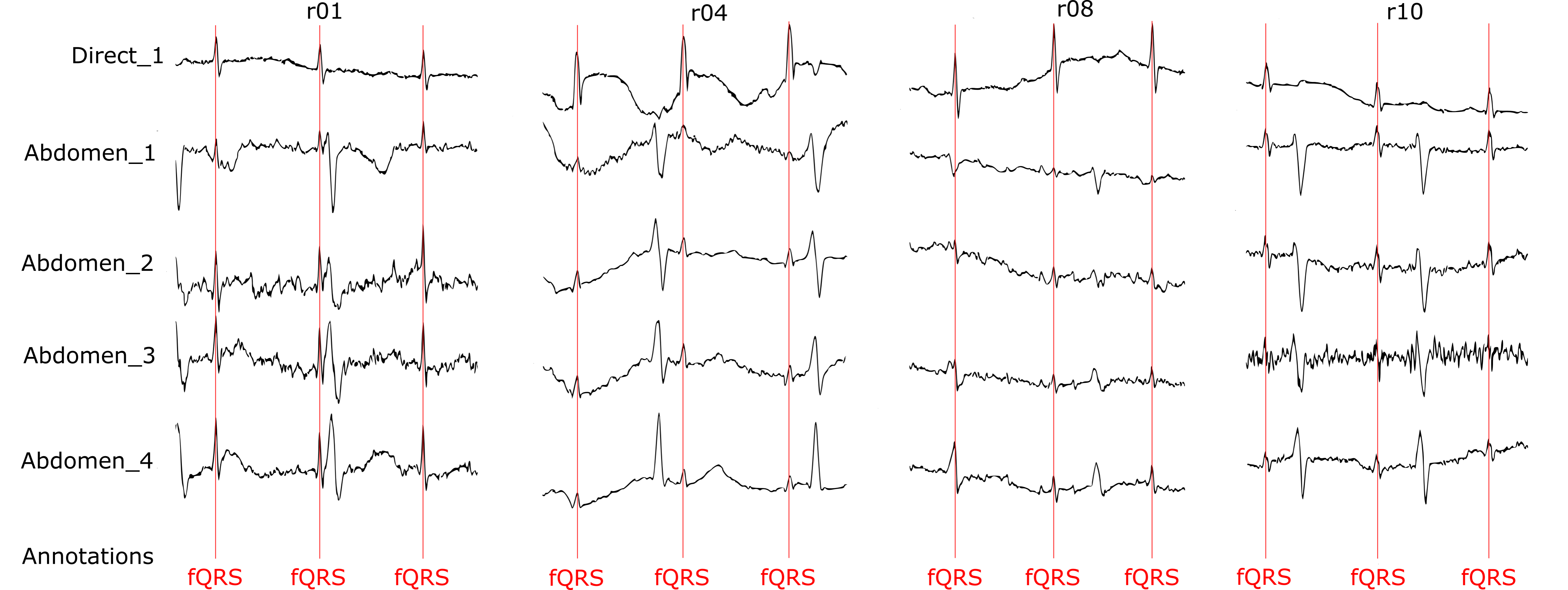

Supplement: Supplementary file 2 [file Data_Sheet_2.ZIP › fig/fig-30.pdf]
