# Supplementary material for: Comparative Effectiveness of ICA and PCA in Extraction of Fetal ECG From Abdominal Signals: Toward Non-invasive Fetal Monitoring
Source: Front Physiol. 2018 May 30;9:648. doi: 10.3389/fphys.2018.00648 (PMC5988877; doi:10.3389/fphys.2018.00648)
Supplement: Supplementary file 2 [file Data_Sheet_2.ZIP › fig/fig-03.pdf]

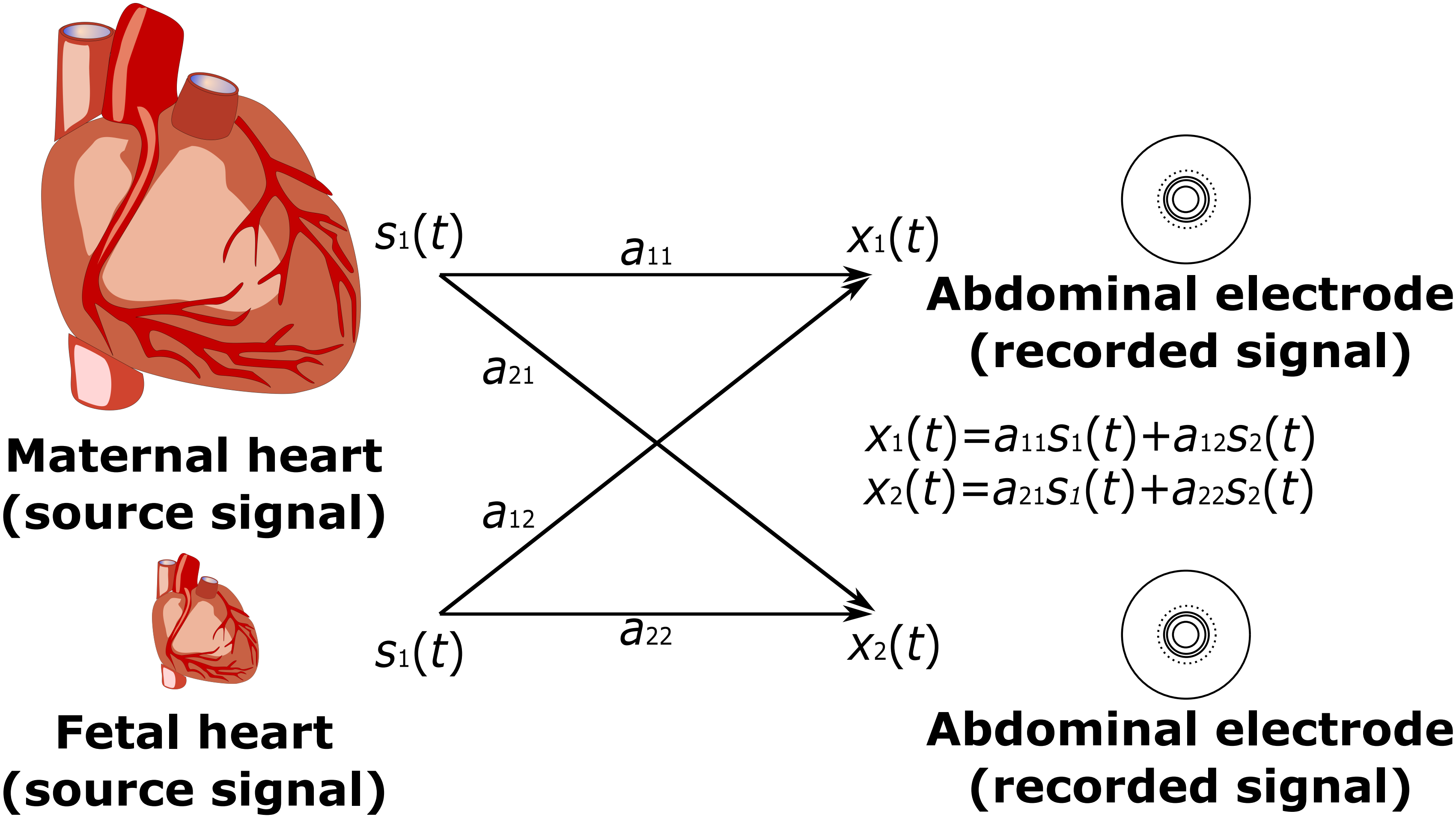

Maternal heart
(source signal)
Fetal heart
(source signal)
$s_1(t)$
$s_1(t)$
a_{11}
a_{21}
a_{12}
a_{22}
$x_1(t)$
$x_2(t)$
Abdominal electrode
(recorded signal)
$x_1(t)=a_{11}s_1(t)+a_{12}s_2(t)$
$x_2(t)=a_{21}s_1(t)+a_{22}s_2(t)$
Abdominal electrode
(recorded signal)
