# Supplementary material for: Comparative Effectiveness of ICA and PCA in Extraction of Fetal ECG From Abdominal Signals: Toward Non-invasive Fetal Monitoring
Source: Front Physiol. 2018 May 30;9:648. doi: 10.3389/fphys.2018.00648 (PMC5988877; doi:10.3389/fphys.2018.00648)
Supplement: Supplementary file 2 [file Data_Sheet_2.ZIP › fig/fig-29.pdf]

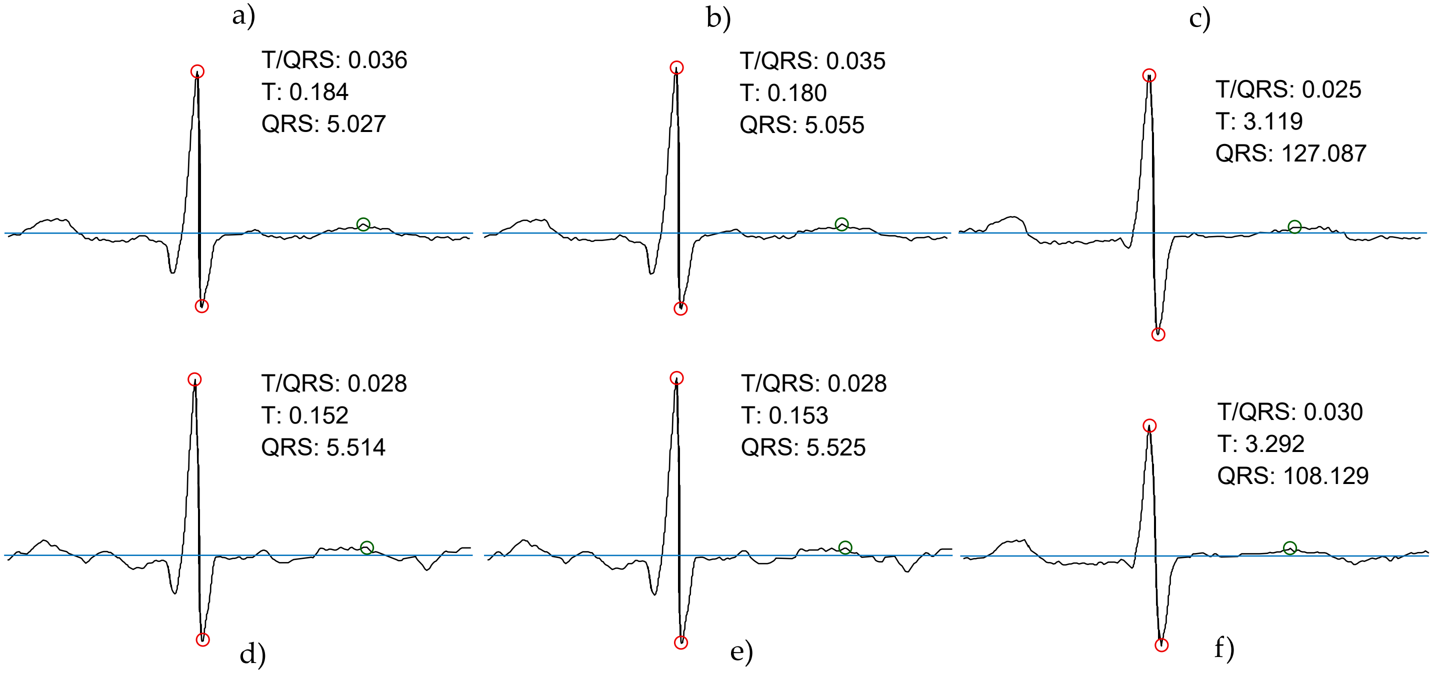

a)
T/QRS: 0.036
T: 0.184
QRS: 5.027
b)
T/QRS: 0.035
T: 0.180
QRS: 5.055
c)
T/QRS: 0.025
T: 3.119
QRS: 127.087
T/QRS: 0.028
T: 0.152
QRS: 5.514
d)
T/QRS: 0.028
T: 0.153
QRS: 5.525
e)
T/QRS: 0.030
T: 3.292
QRS: 108.129
f)
